# Effectiveness of community-based condom distribution interventions to prevent HIV in the United States: A systematic review and meta-analysis

**DOI:** 10.1371/journal.pone.0180718

**Published:** 2017-08-03

**Authors:** Mohsen Malekinejad, Andrea Parriott, Janet C. Blodgett, Hacsi Horvath, Ram K. Shrestha, Angela B. Hutchinson, Paul Volberding, James G. Kahn

**Affiliations:** 1 Phillip R. Lee Institute for Health Policy Studies, University of California, San Francisco, San Francisco, CA, United States of America; 2 Global Health Sciences, University of California, San Francisco, San Francisco, CA, United States of America; 3 The Consortium for the Assessment of Prevention Economics (CAPE), University of California, San Francisco, San Francisco, CA, United States of America; 4 Centers for Disease Control and Prevention, Division of HIV/AIDS Prevention, Atlanta, GA, United States of America; 5 AIDS Research Institute, University of California, San Francisco, San Francisco, CA, United States of America; University of Cyprus, CYPRUS

## Abstract

**Background:**

Despite significant public health implications, the extent to which community-based condom distribution interventions (CDI) prevent HIV infection in the United States is not well understood.

**Methods:**

We systematically reviewed research evidence applying Cochrane Collaboration methods. We used a comprehensive search strategy to search multiple bibliographic databases for relevant randomized controlled trials (RCTs) and non-RCTs published from 1986–2017. We focused on CDI that made condoms widely available or accessible in community settings. Eligible outcomes were HIV infection (primary), sexually transmitted infections, condom use, and multiple sexual partnership. Two reviewers independently screened citations to assess their eligibility, extracted study data, and assessed risk of bias. We calculated risk ratios (RR) with 95% confidence intervals (CI) and pooled them using random-effects models. We assessed evidence quality using GRADE.

**Results:**

We reviewed 5,110 unique records. Nine studies (including one RCT) met eligibility criteria. Studies were conducted in 10 US states between 1989 and 2011. All studies were at high risk of bias. Interventions were categorized into three groups: “Ongoing” (unlimited access to condoms), “Ongoing-plus” (unlimited access to condoms, with co-interventions), and “Coupon-based” (coupons redeemed for condoms). No studies reported incident HIV. Ongoing CDI (four non-RCTs) modestly reduced condomless sex (RR 0.88, 95% CI 0.78 to 0.99). Ongoing-plus CDI (two non-RCTs) significantly reduced multiple sexual partnership (RR 0.37, 95% CI 0.16 to 0.87). Of two coupon-based studies, one (non-RCT) showed reduction in condomless sex in female participants (Odds Ratio 0.67, 95% CI 0.47 to 0.96), while the other one (RCT) showed no effect on STI incidence (RR 0.91, 95% CI 0.63 to 1.31). Evidence quality was “very low” for all outcomes.

**Conclusions:**

CDI may reduce some risky sexual behaviors, but the evidence for any reduction is limited and of low-quality. Lack of biological outcomes precludes assessing the link between CDI and HIV incidence.

## Introduction

Transmission of human immunodeficiency virus (HIV) remains a serious problem in the United States (US). There were over 44,000 new HIV diagnoses in the US in 2014, primarily (93%) acquired through sexual transmission [1]. Most new infections occur in people who do not use condoms or use them inconsistently or incorrectly [2].

Although an estimated 86% of the 1.2 million people living with HIV in the US have been diagnosed and 40% are in care [3], the remainder do not yet know they have the infection and may transmit it to others. Similarly, many HIV-uninfected people do not know the HIV status of their sexual partners [4]. Most HIV-diagnosed patients who are in care in the US receive antiretroviral therapy (ART), which is highly effective in suppressing HIV viral load and thus helps to prevent HIV transmission [5, 6]. However, many patients do not maintain the high level of medication adherence necessary to keep viral load suppressed [7, 8], and may still transmit HIV to their sexual partners.

Regardless of a person’s HIV status awareness or ART adherence level, it is estimated that the correct and consistent use of condoms reduces the risk of sexual transmission of HIV infection by 70% in men who have sex with men (MSM) [9] and 70% in heterosexual couples [10]. Therefore, condom distribution interventions (CDI) have for many years been a mainstay of public health HIV prevention efforts at the federal, state, and local levels. CDI often aim to make condoms widely available, acceptable, and/or accessible to target populations, and they are often implemented in conjunction with other interventions (“co-interventions”) to directly or indirectly enhance the effect of condom distribution by addressing knowledge, attitudes, behaviors, and the social and economic contexts influencing condom use. However, the degree to which such programs actually have an impact on HIV incidence, particularly in various risk groups, and the effect of co-interventions on HIV outcomes are poorly understood.

There are two systematic reviews [11, 12] that focused on the effect of condom distribution in community settings for preventing HIV infection internationally. However, each review defined “community” differently. While Charania [11] considered the term to reflect organizations and institutions serving non-geographically defined sub-populations (e.g., “the gay community”), Moreno [12] understood “community” in terms of a city, district, or other geographic demarcation. While neither sense of the term is incorrect, as a practical matter it is likely better for systematic reviews to include studies using both concepts. Further, Charania [11] included both randomized controlled trials (RCTs) and non-RCTs comprising several US-based studies, while Moreno (2014) included only RCTs, but none from the US. The reviews had no overlap among included studies, neither found evidence that CDI reduced the risk of HIV incidence, and both found evidence for improvement in sexual risk behavioral outcomes.

### Rationale for this review

Charania’s review [11] is now out of date, as its searches were conducted in 2007. Moreno’s review [12] was too narrowly framed in terms of eligible study designs and conceptualization of “community” to capture studies from the US. Given the overall limitations of this evidence-base, we conducted a systematic review to provide an up-to-date assessment of the impact of community-based CDI on HIV incidence among general and high-risk populations in the US, and we estimate added value of co-interventions.

## Methods

Throughout the review, we developed and applied methods based on those of the Cochrane Collaboration [13] and reported findings according to Preferred Reporting Items for Systematic Reviews and Meta-Analyses recommendations (PRISMA; S1 File) [14]. Our protocol (S2 File) was reviewed and approved by the CDC’s Division of HIV and AIDS Prevention on July 18, 2015.

### Eligibility criteria

We used the population, intervention, comparator, and outcome (PICO) schema to detail our study eligibility criteria.

#### Population

Eligible studies included the sexually active general population (per definitions by study investigators) and populations at high risk of acquiring or transmitting HIV infection (adolescents age 10–19; heterosexual adults who have multiple sex partners, who frequently change sex partners, or whose sexual behaviors otherwise could be considered to increase HIV risk; homeless people; MSM; people who inject drugs, and sex workers). We excluded studies targeting populations in closed institutional settings (e.g., jails, hospitals). We provisionally included studies conducted in school settings, but subsequently decided to report school-based studies separately [15].

#### Intervention

Eligible studies included any intervention that aimed to increase the availability and accessibility of condoms (male or female) through provision of free (or financially subsidized) condoms. Studies providing coupons for redemption of condoms were eligible for inclusion. We only included studies where condom distribution was an integral component of the intervention: condoms had to be provided with the clear goal of increasing accessibility and/or affordability and not used only as an incentive for participation or provided as samples. Condoms must have been available throughout the intervention period. We excluded interventions described as “brief” and studies in which condoms were distributed along with other forms of contraceptives and with a strong focus on preventing pregnancy rather than preventing STIs.

CDI could be integrated with or supplemented by any co-interventions that directly or indirectly enhance the effect of condom distribution by addressing knowledge, attitudes, behaviors, and the social and economic contexts influencing condom use including: a) social marketing/mass-media campaigns to promote condom use; b) risk-reduction or other prevention interventions directly or indirectly promoting condom usage acceptance; c) community-wide mobilization efforts to support condom use; d) changes in policies or laws to promote condom use; or e) individual-, couple-, or group-level behavioral interventions (e.g., counseling, motivational interviewing). Evaluations of programs with co-interventions outside of these five categories (e.g., HIV testing and/or needle exchange programs) were not eligible, unless condom distribution outcome data were stratified and reported separately from those of the co-interventions. We also excluded theory-based behavioral interventions (e.g., cognitive-behavioral group interventions [16]) that aim to reduce sexual risk behavior and/or promote condom use, without making condoms widely available and accessible to participants.

#### Comparator

Comparators could have included no intervention (e.g., a pre-intervention condition), or a component of the intervention apart from provision of condoms (e.g., HIV prevention education only).

#### Outcomes

Our primary outcomes of interest were 1) change in HIV incidence or prevalence attributable to the intervention, and 2) laboratory-confirmed HIV diagnosis. As proxy (indirect) measures for these outcomes, studies reporting sexually transmitted infection (STI) incidence or prevalence, any variation of self-reported condom use (e.g., “at last sex,” “always vs. sometimes,” “always vs. never,” “mean percentage of episodes using condoms,” etc.), and self-reported number of sex partners were eligible. As condom-use outcomes are reported in many different ways, with no single generic metric [17], we developed two standardized metrics as described under “Data analysis plan.” Number of sex partners was included in order to capture the potential risk compensation effect of CDI (i.e., lower perceived risk through condom use could result in an increase in number of sexual partners or other types of risky behavior) [18]. We excluded studies that reported only female condom use outcomes without providing a measure for overall condomless sex.

We had no restrictions on study eligibility based on language or publication status. We also included all types of observational and experimental designs as long as reported data would allow a comparison between intervention and control conditions.

### Search methods

We attempted to minimize the potential for publication bias by comprehensively searching multiple sources of studies including bibliographic databases, archives of relevant conference abstracts, bibliographies of previous systematic reviews, bibliographies of our included studies, and registries of clinical trials. We also contacted authors of included studies to be sure we had not missed any of their ongoing or unpublished research.

Using a range of relevant keywords and Medical Subject Heading (MeSH) terms, we developed a comprehensive search strategy (S3 File). The search range was from January 1, 1986 (the year in which CDC first recommended consistent condom use for HIV prevention [19]) to the search date (June 23, 2015; searches updated April 17, 2017). We searched several bibliographic databases including the Cochrane Central Register of Controlled Trials (CENTRAL), PubMed, PsycINFO, and Scopus (including EMBASE records from 1996 to the present). We improved the sensitivity of our search strategies by iteratively updating them with keywords from relevant studies not detected in initial searches.

To augment these database searches, we also searched “grey literature” to obtain data reported outside peer-reviewed journals. Grey literature sources included the New York Academy of Medicine’s Grey Literature Report; abstract archives of International AIDS Conference, the International AIDS Society Conference on HIV Pathogenesis, Treatment and Prevention (IAS), and the Conference on Retroviruses and Opportunistic Infections (CROI); and doctoral dissertations through ProQuest Dissertations. We also used advanced search syntax in Google and Google Scholar to conduct targeted searches of web sites of relevant non-government organizations and of US federal, state, and local government HIV prevention programs. We searched ClinicalTrials.gov at the US National Institutes of Health to identify any ongoing RCTs.

### Methods for selection of studies

We examined studies for relevance based on geographic settings, intervention, design, types of participants, and outcome measures, and in a step-wise fashion determined which studies met inclusion criteria. First, using the Endnote X7.4 software [20], we excluded duplicate records from various sources. Two study authors (AP and JB) then read the titles, abstracts, and descriptor terms of the remaining citations to identify potentially eligible studies. After reconciling their respective selections, the two reviewers independently examined the full text of each article and determined which met our review’s inclusion criteria. Any differences arising were resolved by discussion with a third reviewer (MM).

### Data extraction and management

From studies meeting inclusion criteria, two reviewers working independently extracted data into a pre-piloted data extraction form. They then cross-checked each other’s extracted data, corrected errors, reconciled any disagreements as they arose, and contacted study authors to obtain key data missing from reports.

To inform our analyses, study data was extracted by domain: a) complete citation; b) geographical setting; c) details of interventions and comparators (e.g., intervention content, duration of exposure to the intervention); d) details of participants (e.g., age, sex); e) outcomes (e.g., definitions and descriptions of outcomes; details of how outcomes were assessed); f) detail of study implementation (e.g., study inclusion and exclusion criteria, length of follow-up); and g) risk of bias assessment data (i.e., details necessary to perform a bias risk assessment using the Cochrane tool, described below).

### Risk of bias assessment

We adapted and used the Cochrane Collaboration instrument for assessing risk of bias [13] that examines individual studies across seven domains for RCTs: sequence generation, allocation concealment, blinding of participants and personnel, blinding of outcome assessment, incomplete outcome data, selective outcome reporting, and other potential biases. For non-RCTs, we additionally assessed risk of bias by using criteria recommended by the GRADE Working Group (S1 File) [21].

### Statistical analysis and data synthesis

#### Effect size calculation

For each outcome extracted from primary studies, we calculated risk ratios (RR) and their associated 95% confidence intervals (CI). When studies reported odds ratios (OR) for non-rare outcomes and there were insufficient data to calculate the RR, we used the Zhang and Yu [22] method to obtain an estimate of the RR. When studies did not report 95% CI, we calculated 95% CI from *p*-values or from sample sizes as necessary. When data were insufficient to estimate CIs and assumptions could not be made about sample sizes, we excluded the outcome from meta-analyses.

#### Data analysis plan

In preparation for conducting meta-analyses, we grouped data points according to the characteristics of three domains: 1) intervention type, 2) outcome type, and 3) population (see Table 1 for details). This paper focuses on CDI types that made condoms more widely available per CDC’s recommendations [23]. We distinguished three types of CDI based on the presence of co-interventions and use of coupons to provide access to condoms: Ongoing, Ongoing-Plus, and Coupon-based. We separately analyzed data on a group of CDI (Limited) that were implemented at the individual context level and were limited in terms of frequency and/or duration of access to condoms.

**Table 1:** Meta-analysis plan by type of community-based condom distribution intervention, outcome, population.

Type of condom distribution intervention*	Outcome	Population
**Ongoing (without co-intervention)**: Participants had free access to unlimited number of condoms at specific locations; and were able to get more at essentially any time. Without co-intervention means condom distribution (CD) itself was the only intervention component being tested in the study (i.e., the comparison group received the same co-interventions, apart from the CD component; or the comparison group received no intervention). **Ongoing-plus (with co-intervention):** Similar to above but CD was a significant component of a multicomponent intervention (e.g., a community level intervention including media, outreach, and CD). **Coupon-based (with co-intervention)**: Participants received coupons or cards and could exchange these for condoms at specific locations. CD was a significant component of a multicomponent intervention	1. **Condomless sex likelihood**: Self-report of having unprotected sex at last episode, or the proportion of a set of episodes that were unprotected. 2. **Not always using condoms**: Self-report of having at least one episode of condomless sex in the recall period. 3. **Multiple sexual partnership**: Self-report of having at least two (cut off as defined in studies and can be larger) sexual partners in the recall period. 4. **HIV**: Incident case of HIV infection based on any data source such as lab reports, medical record, surveillance reports, and self-report. 5. **Other STI**: Incident case of non-HIV STIs based on any data source (same as above). **Sub-analysis by short (< 1 year of follow-up) vs. long term follow-up:** Short term includes studies in which we know the length of time since the baseline measure (or have clear information about how long the intervention was going on at the time of the assessment), and does not include, for example, cross-sectional studies measuring an effect based on current presence/absence of an intervention where we cannot measure how long the intervention had been going on.	**Overall analysis:** Combining across multiple populations (and follow-up time points if applicable) within a specific intervention and outcome type. **Sub-analysis by:** a. **Sex** (Males/Females) b. **HIV high risk group** (Drug users, MSM)

* We separately analyzed and reported a group of studies (Limited) that initially met our broad inclusion criteria, but that were implemented at the individual context level and were limited in terms of frequency and/or duration of access to condoms (e.g., participants could take as many condoms as they wanted, but only at motivational sessions or when they made contact with a street outreach worker). See S5 File for details.

We observed a wide variation in measuring and reporting of sexual risk behavior outcomes in the literature. Given there is no universally-accepted standard metric for measuring condom use [17], we created two standardized outcomes: a) condomless sex likelihood, at last episode (most commonly reported, k = 5) or over a recall period (k = 3), and b) not always using condoms. For studies reporting condomless sex at episodes of sex other than last, we considered those outcomes similar enough and included as condomless sex likelihood. Additionally, sexual partnership outcomes were reported in two ways: mean counts of partners in the recall period and proportion reporting more than a certain number of partners in the recall period. In order to combine these two types, we created a “multiple sexual partnership” outcome, assumed a Poisson distribution of the number of partners, and converted mean numbers of partners to the proportion of participants with two or more partners.

#### Meta-analysis

For statistical analysis, we used the Review Manager 5 software [24]. When we identified two or more data points for the same combination of intervention-outcome pair, we used a random-effects meta-analytic model to calculate pooled RR and 95% CI, weighting by inverse of variance. To calculate an overall estimate within a study based on sub-group data (e.g., condom use by males and females), we used fixed-effect models [25]. We also assessed statistical heterogeneity for pooled data using the I^2^ statistic, which estimates the proportion of variation in effect sizes that is explained by non-random differences in intervention effect [26]. We explored subgroup analyses for each outcome if they were available.

### Assessment of evidence quality

We used the GRADE approach [27] to assess the quality of evidence for each outcome across the literature. The GRADE methodology defines “quality of evidence” as “the extent of our confidence that the estimates of effect are correct" [13]. The quality rating across studies has four levels: high, moderate, low, or very low. Data from randomized trials are considered to be of high quality but can be downgraded for any of five reasons: risk of bias, indirectness of evidence, unexplained heterogeneity or inconsistency of results, imprecision of results, high probability of publication bias. In contrast, data from non-RCTs are considered to be of low-quality, but can be upgraded for any of three reasons: large magnitude of effect, plausible confounding that would increase confidence in an estimated effect, the presence of a dose-response gradient. We used GRADEpro software [28] to generate GRADE evidence profiles.

## Results

**Fig 1:**
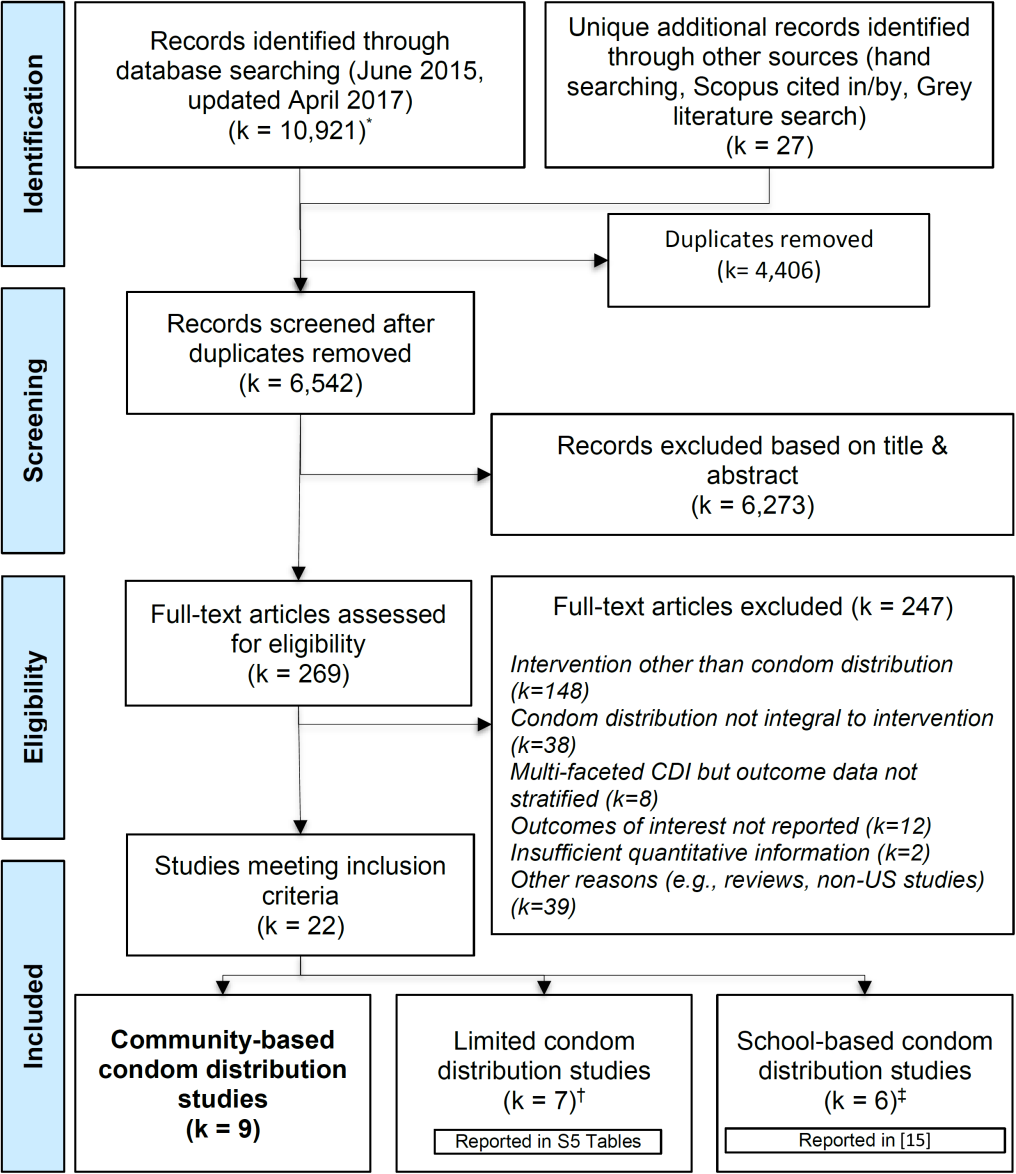
Searching and screening of scientific records for systematic review of community-based condom distribution interventions in the United States (search January 1, 1986 to April 17, 2017). *Databases searched: SCOPUS, PubMed, PsycINFO, Cochrane Central Register of Controlled Trials. †We separately analyzed and reported this group of studies. They initially met our broad inclusion criteria, but were implemented at the individual context level and were limited in terms of frequency and/or duration of access to condoms (e.g., participants could take as many condoms as they wanted, but only at motivational sessions or when they made contact with a street outreach worker). See S5 File for details. ‡Given the differences between youth populations in school and high-risk adult populations in community settings, we decided while screening of studies was in progress to disseminate findings of school-based studies separately.

Our searches identified 10,947 records: 10,921 from database searches and 27 from other sources (Fig 1). After removing 4,406 duplicates, two authors reviewed titles, abstracts, and keywords for the remaining 6,542 records and excluded an additional 6,273 citations for failing to meet inclusion criteria. They then examined the full text of 269 articles for further assessment, of which 247 were excluded for not meeting at least one of our inclusion criteria (see S4 File for citations): intervention content not including condom distribution (k = 148); condom distribution not an integral part of the intervention (k = 38); multifaceted intervention without stratified outcome data for the effect of CDI (k = 8); not reporting outcomes of interest (k = 12); not reporting sufficient quantitative information to calculate a point estimate and/or CI (k = 2); and other reasons such as being conducted outside of the US or not being a primary program evaluation (k = 39).

We corresponded with 20 study authors and other experts seeking information on ongoing or unpublished research and received responses from 10, but this process did not yield any new studies. In the end, we analyzed data for 22 studies that met our general definition of CDI, of which 16 studies were conducted in community settings and six in school settings (separately reported; [15]). Of 16 community-based studies, seven were considered “Limited” in terms of provision of condoms and are only presented in the appendix (see S5 File), while the rest of this manuscript focuses on the remaining nine community-based CDI studies.

### Characteristics of included studies

Table 2 presents characteristics of the nine included studies. All were published in peer-reviewed journals between 1992 and 2013 and carried out within a 22-year time span (1989 to 2011) in 10 US states: California (k = 2), Connecticut, Louisiana, Massachusetts, Minnesota, Nevada, Oregon, Pennsylvania, Texas, and Washington (k = 2). All studies but one were non-RCT in design. Eight reported only behavioral outcomes (e.g., condom-use related), and one reported only incident non-HIV STIs. Follow up ranged from five to 36 months.

**Table 2:** Characteristics of community-based condom distribution programs in the United States included in systematic review, by intervention category^a^.

Author & Year	Study location (Setting)	Data Collection Year	Target population (inclusion/exclusion criteria)	Demographic information	Co-interventions (Provider / delivery modality)^b^	Study design^c^ (Number of participants)	Reported outcomes	Follow-up period
**Ongoing (without co-interventions)**								
**Calsyn 1992 [29]**	Seattle, WA (Urban)	Dec 1989—May 1990	Male drug users (all men receiving outpatient drug abuse treatment at the VA Med. Center)	Age: 8.7% <35 yrs.; 66.1% 35–44; 17.4% 45–55; 7.8% >55 Sex (% F): 0% Race/Ethnicity: 76.7% White; 22.3% Black; 1.0% Other	None (N/A)	Experimental^d^ Pre-Post Single-Arm Cluster (103)	Mean use of condoms for vaginal intercourse events (past 2 mo.)	5 mo.
**Cohen 1999 (Area B) [30]**	New Orleans, LA (Urban)	1994–1996	African-American men (age 15–45 yrs., approached in high risk neighborhoods of New Orleans)	Age: mean 29.3 yrs. Sex (% F): 0% Race/Ethnicity: 100% Afr. Amer.	None (N/A)	Single-Arm Pre-Post Cross-Sectional (≥940)^e^	Condom use at last sex; At least 2 sex partners (past yr.)	1 yr; 2 yrs.
**Eisenberg 2013 [31]**	Statewide, MN (Urban/rural)	2010–2011	Young adults (undergraduate student recruited from selected colleges/ universities, age 18–24 yrs., sex. active in the past yr, and not married)	Age: range 18–24 yrs. Sex (% F): 63% Race/Ethnicity: 83.6% White; 3.0% Black; 2.8% Hisp.; 5.5% Asian/Pacific Islander; 2.3% Amer. Indian/Alaskan Native; 2.9% mixed/other	None (N/A)	Ecologic (6,318)	No condom at last intercourse	N/A
**Ross 2004 (Comparison arm) [32]**	Houston/ Harris County, TX (Urban)	1998–2000	Gen. population (age 18+ yrs. and living in zip codes with relatively high rates of syphilis)	Age: NR Sex (% F): 41.6% Race/Ethnicity: 92.3% Afr. Amer.; 2.2% White	None (N/A)	Experimental Single-Arm Pre-Post Cross-Sectional (789 across 2 cross-sect. waves)	Proportion of times used condoms of times had sex (past 4 weeks); Number of sex partners (past 4 weeks)	2 yrs.
**Ongoing-plus (with co-interventions)**								
**Alstead 1999 [33]**	King County, Seattle area, WA (Urban)	1995	Youth (age 15–17 yrs.)	Age: mean(SD) 16.0 (.8) yrs. Sex (% F): 48% Race/Ethnicity: 38% White; 30% Afr. Amer.; 16% Asian/Pacific Islander; 16% other (incl. Hisp., Native Amer., and multi-ethnic)	Behavioral & skill building (Media)	Pre-Post Single-Arm Cross-Sectional (1,425 across 3 cross-sectional waves)	Condom use at last intercourse	1–7 mo.
**Lauby 2000 [34]**	Pittsburgh, PA; West Philadelphia, PA; Portland, OR (Urban)	1993–1996	High risk women (age 15–34 yrs., approached in a high risk community, sexually active in the past 30 days)	Age: mean 25 yrs.Sex (% F): 100%Race/Ethnicity: 73.1% Afr. Amer.	Behavioral & skill building + Street outreach (Peers + Media)	Experimental Double-Arm Pre-Post Cross-Sectional (3,723)	Condom use during most recent sex; Consistent condom use (past 30 days)	36 mo.
**Ross 2004 (Intv. arm) [32]**	Houston/ Harris County, TX (Urban)	1998–2000	General population (age 18+ yrs., living in zip codes with relatively high rates of syphilis)	Age: NR Sex (% F): 37.6% Race/Ethnicity: 91.0% Afr. Amer.; 4.2% White	Behavioral and skill building + Street outreach (Peers + Media)	Experimental Single-Arm Pre-Post Cross-Sectional (841 across 2 cross-sect. waves)	Proportion of times used condoms of times had sex (past 4 weeks); Number of sex partners (past 4 weeks)	2 yrs.
**Sellers 1994 [35]**	Boston, MA; Hartford, CT (Urban)	Sept 1989-Dec 1991	Latino youth (age 14–20 yrs.)	Age: range 14–20 yrs. Sex (% F): NR Race/Ethnicity: 100% Hisp. (94% Puerto Rican)	Gen. HIV & sex health edu + Formal CUT + Street outreach (Professional staff + Peers + Media)	Experimental Non-RCT Double-Arm Cluster (586)	Multiple (2+) sexual partners (past 6 mo.)	18 mo.
**Coupon-based (with co-interventions)**								
**Bull 2008 [36]**	CA: Oakland, San Francisco, Los Angeles, San Diego; NV: Las Vegas (Urban)	2004–2005	Young adult women (15–25 yrs.)	Age: 41.9% 15–17 yrs.; 18.5% 18–19; 39.5% 20–25; 0.1% missing Sex (% F): 100%Race/Ethnicity: 32.3% Afr. Amer.; 35.4% Latina; 30.1% other; 2.2% missing	Gen. HIV & sex health edu + Behavioral & skill building (Media)	Cross-Sectional (3,003 interviewed at follow-up)	Used a condom at last sex	7–10 mo.
**Cohen 1992 [37]**	Los Angeles, CA (Urban)	NR	Gen. pop. (approached at a public health STD clinic)	Age: median 27.9 yrs. (men in study); 26.6 (women in study) Sex (% F): 40.2% Race/Ethnicity: 20.6% Hisp.; 71.5% Black; 4.5% White; 3% Asian; 3.5% Other or unknown	Formal CUT + Behavioral & skill building (Professional staff)	Randomized Controlled Trial (analyzed condom distr. and control groups only; 503)	STD reinfection	6–9 mo.

**Legend:** CD, Condom distribution; CUT, Condom use training; MSM, Men who have sex with men; N/A, Not applicable; NR, Not reported; PWID, People who inject drugs; STD, Sexually transmitted disease.

^a^ We separately analyzed and reported a group of studies (Limited) that initially met our broad inclusion criteria, but that were implemented at the individual context level and were limited in terms of frequency and/or duration of access to condoms (e.g., participants could take as many condoms as they wanted, but only at motivational sessions or when they made contact with a street outreach worker). See S5 File for details.

^b^ Intervention category and co-interventions listed are those that comprise the unique elements tested in the study (i.e., common elements provided to both the intervention and control group are not listed).

^c^ Study design reflects the way reported data were analyzed in this review in order to extract an effect of condom distribution. It does not always match the design of the study as originally implemented.

^d^ Studies are considered experimental if investigators controlled the intervention allocation.

^e^ Total number of respondents not reported. Maximum item-level N reported in the publication was 941 for Area B across 3 waves.

Studies were diverse in respect to target populations as follows: general population, residing in or approached in a high HIV prevalence area (k = 2, 22% of total) [30, 32]; youth or young adults (k = 6, 67%) [31, 33–37]; drug users (k = 1, 11% of total) [29].

One of the nine studies [32] reported two distinct intervention-control comparisons, resulting in a total of 10 unique comparisons for our data analysis. Of the 10 unique comparisons, four (40%) assessed the effect of Ongoing CDI, without co-interventions [29–32]; four (40%) assessed Ongoing CDI, with co-interventions [32–35], and two (20%) assessed condom coupon or card distribution, with co-interventions [36, 37].

### Risk of bias in included studies

Of the nine included studies, only one [37] was an RCT. It was unclear from the report whether the trial’s methods for randomization and to control for attrition bias were adequate. The measures undertaken for blinding of participants, personnel, and outcome assessors were adequate.

The remaining studies, all non-RCTs, were subject to additional high risk of bias. This was partly due to the fact that the vast majority of outcomes (condom use and of number of sexual partners) were self-reported by respondents (i.e., potentially biased due to social desirability norms, especially in a study’s intervention arm). Thus, we determined that measurement of outcome was significantly flawed in all reported behavioral outcomes. Further, several studies used single-arm pre-post design to assess the effect of condom use [29, 30, 32, 33] and therefore were at high risk of bias due to secular trends (i.e., in the absence of a distinct control condition, the observed effect can be partially or entirely due to other existing interventions or unknown sources). Finally, there was a risk of intervention contamination in studies where an intervention was provided to a selected neighborhood and a similar neighborhood in the same geographic area was used as comparison [34].

### Results of data synthesis

Table 3 summarizes the evidence for the effectiveness of community-based CDI on risk of HIV transmission by intervention type, outcome, and population. Comprehensive and detailed results of our meta-analyses, as well as risk of bias assessments and GRADE evidence profiles are presented in S6 and S7 Files, respectively.

**Table 3:** Summary of evidence for the effectiveness of community-based condom distribution interventions by intervention type, outcome, and population type in the United States^a^.

Outcome	Population (N)	Risk Ratio (95% CI)	Quality of Evidence^b^	Citations
**Ongoing (without co-interventions)**				
Condomless sex likelihood	Overall (8,091)	**0.88 (0.78–0.99)**	⊕⊖⊖⊖	[29–32]
	Male (984)	**0.83 (0.75–0.91)**	⊕⊖⊖⊖	[29, 30]
	Drug users (51)	0.83 (0.69–1.00)	⊕⊖⊖⊖	[29]
Multiple sexual partnership	Overall (1,696)	0.59 (0.19–1.86)	⊕⊖⊖⊖	[30, 32]
	Male (907)	1.06 (0.98–1.15)	⊕⊖⊖⊖	[30]
**Ongoing-plus (with co-interventions)**				
Condomless sex likelihood	Overall (>4,494)	0.98 (0.88–1.09)	⊕⊖⊖⊖	[32–34]
	Female (>3,229)	0.93 (0.81–1.07)	⊕⊖⊖⊖	[34]
Not always using condoms	Female (>3,229)	0.91 (0.71–1.17)	⊕⊖⊖⊖	[34]
Multiple sexual partnership	Overall (1,243)	**0.37 (0.16–0.87)**	⊕⊖⊖⊖	[32, 35]
	Male (NR)	0.90 (0.43–1.88)	⊕⊖⊖⊖	[35]
	Female (NR)	**0.06 (0.01–0.36)**	⊕⊖⊖⊖	[35]
**Coupon-based (with co-interventions)**				
Incident STI	Overall (503)	0.91 (0.63–1.31)	⊕⊖⊖⊖	[37]
	Male (301)	0.85 (0.56–1.29)	⊕⊖⊖⊖	[37]
	Female (202)	1.18 (0.52–2.68)	⊕⊖⊖⊖	[37]
Condomless sex likelihood	Female (2,005)	**0.67 (0.47–0.96)**^**c**^	⊕⊖⊖⊖	[36]

**Legend:** GRADE Working Group grades of evidence:

⊕⊕⊕⊕, HIGH: We are very confident that the true effect lies close to that of the estimate of the effect. Further research is unlikely to substantially change the estimate

⊕⊕⊕⊖, MODERATE: We are moderately confident in the effect estimate: The true effect is likely to be close to the estimate of the effect, but there is a possibility that it is substantially different

⊕⊕⊖⊖, LOW: Our confidence in the effect estimate is limited: The true effect may be substantially different from the estimate of the effect

⊕⊖⊖⊖, VERY LOW: We have very little confidence in the effect estimate: The true effect is likely to be substantially different from the estimate of effect.

^a^ We separately analyzed and reported a group of studies (Limited) that initially met our broad inclusion criteria, but that were implemented at the individual context level and were limited in terms of frequency and/or duration of access to condoms (e.g., participants could take as many condoms as they wanted, but only at motivational sessions or when they made contact with a street outreach worker). See S5 File for details.

^b^ See S7 for further details on quality of evidence ratings.

^c^ Effect measure is odds ratio.

**Fig 2:**
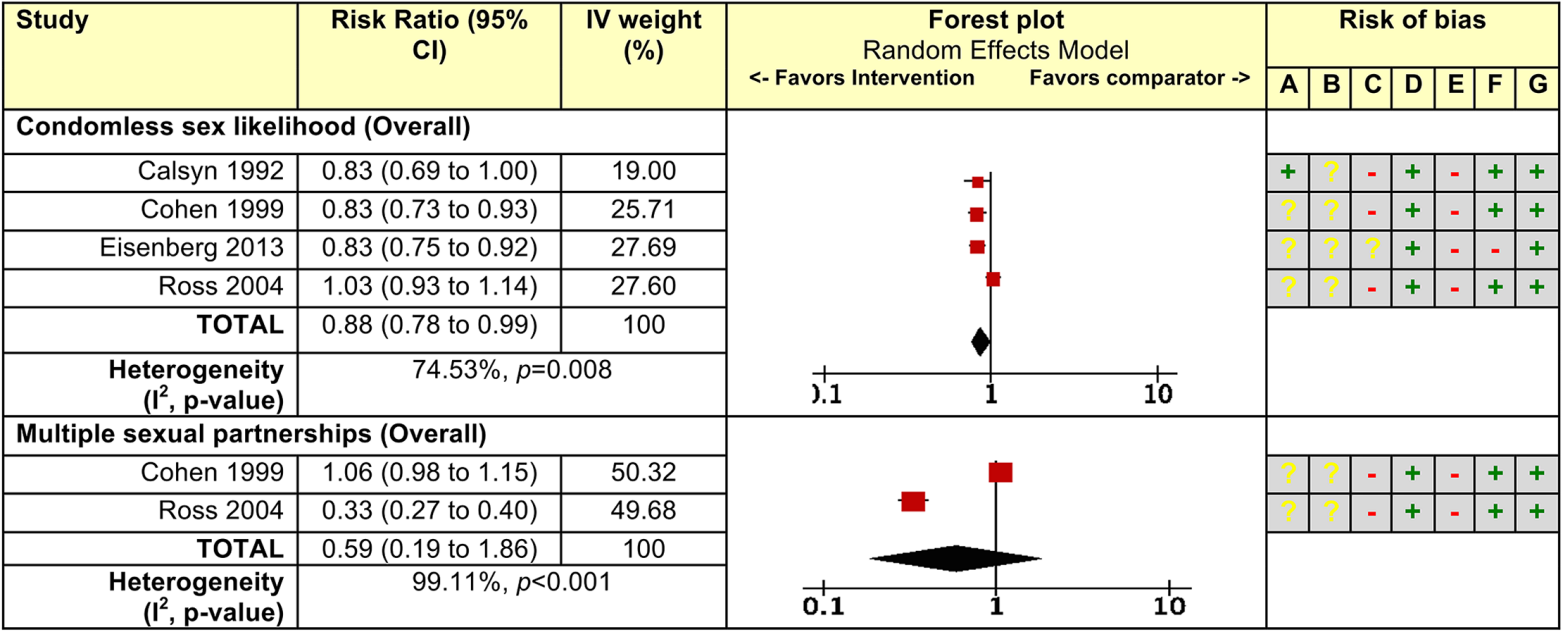
Pooled effect measures and risk of bias for the effect of “Ongoing” community-based condom distribution interventions (compared to no condom distribution) for sexual risk behaviors in the United States. **Legend:** CI, Confidence interval; IV, Inverse variance. Size of red square on the forest plots represents IV weights. See S6 for further details on risk ratio calculations. **Risk of bias legend:** (A) Incomplete outcome data (attrition bias); (B) Selective reporting (reporting bias); (C) Other bias; (D) Failure to develop and apply appropriate eligibility criteria; (E) Flawed measurement of exposure and/or outcome; (F) Failure to control for confounders; (G) Too-short or incomplete length of follow-up;— = high risk of bias; **+** = low risk of bias;**?** = unclear risk of bias.

Our analysis estimated that Ongoing CDI (k: number of comparisons = 4, n: number of participants = 8,091) with pooled RR of 0.88 (95% CI 0.78 to 0.99) significantly reduced the risk of condomless sex likelihood. For multiple sexual partnership (k = 2, n = 1,696) the RR was 0.59 (95% CI 0.19 to 1.86). We observed significant statistical heterogeneity for each outcome (I^2^ = 74.5%, *p* = 0.008; and I^2^ = 99. 1%, *p*<0.001; respectively). All study outcomes were at high risk of bias (Fig 2). All studies were at high risk of bias for the flaws in measurement of exposure and outcome (domain E), and all except one due to other risk of bias (domain C, mainly secular trend and contamination; Fig 2).

**Fig 3:**
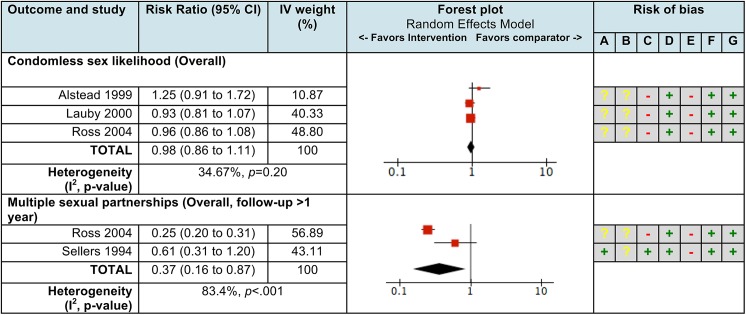
Pooled effect measures and risk of bias for the effect of “Ongoing-plus” community-based condom distribution interventions (compared to no condom distribution) for sexual risk behaviors in the United States. **Legend:** CI, Confidence interval; IV, Inverse variance. Size of red square on the forest plots represents IV weights. See S6 for further details on risk ratio calculations. **Risk of bias legend:** (A) Incomplete outcome data (attrition bias); (B) Selective reporting (reporting bias); (C) Other bias; (D) Failure to develop and apply appropriate eligibility criteria; (E) Flawed measurement of exposure and/or outcome; (F) Failure to control for confounders; (G) Too-short or incomplete length of follow-up;— = high risk of bias; **+** = low risk of bias;**?** = unclear risk of bias.

For Ongoing-plus CDI, the pooled RR for condomless sex likelihood (k = 3, n = 4,494) and multiple sexual partnership (k = 2, n = 1,243) were 0.98 (95% CI 0.88 to 1.09) and 0.37 (95% CI 0.16 to 0.87), respectively. Heterogeneity in the effects for condomless sex likelihood was moderate (I^2^ = 35%, *p* = 0.20), but was high across the two estimates of multiple sexual partnership (I^2^ = 83%, *p*<0.001). One study measured not always using condoms (n = 3,229), with a RR of 0.91 (95% CI 0.71 to 1.17). All analyses were affected by high risk of bias in the same domains as those in Ongoing CDI (Fig 3).

The effect of Coupon-based CDI was assessed in two studies [36, 37] (Table 3). In the single RCT [37], the RR for incident STI infection was as follows: 0.91 (95% CI 0.63 to 1.31) in the overall samples; 0.85 (95% CI 0.56 to 1.29) among men; and 1.18 (95% CI 0.52 to 2.68) among women. In a non-RCT [36], the odds ratio for condomless sex likelihood was 0.67 (95% CI 0.47 to 0.96; insufficient data to transform odds ratio to RR).

## Discussion

Our systematic review found that community-based CDI in the US may reduce risky sexual behaviors such as condomless sex and multiple sexual partnership. However, quality of evidence was very low, reflecting high risk of bias and a lack of direct biological evidence for reduced HIV incidence.

Only one of nine included studies assessed a biologic outcome (non-HIV STI incidence), with non-significant results. Self-reported behavioral outcomes included condom use (k = 8) and change in the number of sexual partners (k = 4). Results reached statistical significance in only 6 out of 21 outcomes (see S7 File), including main analyses, subgroup analyses, and single-study estimates. Significant findings were all related to outcomes of condom use (k = 4) and number of sexual partners (k = 2). These included three condom use outcomes in studies of Ongoing distribution, two multiple sexual partnership outcomes in Ongoing-plus studies, and one condom use outcome in a study using the Coupon-based approach.

As outlined below, the overall body of evidence across all intervention types and for all main and subgroup analyses was of very low quality.

In pooled data from four non-RCTs using the Ongoing approach, which is closely aligned with CDC’s definition of CDI, we found very low-quality evidence of 12% reduction in condomless sex likelihood. The effect was slightly more pronounced in subgroup analyses of two studies reporting male participant outcomes and two with follow-up ≤12 months. Very low-quality evidence from two studies showed no difference in self-reported multiple sexual partnership. All other outcomes examined for this approach were not statistically significant, with evidence quality rated very low.

In the Ongoing-plus model, we found very low-quality evidence from a pooled analysis of three non-RCTs that condomless sex likelihood did not differ between groups. However, across two of these studies, both at high risk of bias, there was very low-quality evidence showing a 63% reduced risk of multiple sexual partnership. An apparent 94% risk reduction was shown in one study reporting data from female participants. All other outcomes examined for this approach were not statistically significant, with evidence quality rated very low.

In the Coupon-based model, the quality of evidence was again very low. The single included RCT provided very low-quality evidence for no statistically significant effect on incident STIs. One non-RCT in women provided very low-quality evidence associating the intervention with reduced condomless sex likelihood.

Overall, while some evidence suggests CDI may help to reduce HIV risk behavior by about 12–15%, with larger estimated reductions in multiple sexual partnership, it is very uncertain that such programs have any impact at all on HIV incidence. Evidence quality for all outcomes was very low, and was graded down for serious risk of bias and serious indirectness (i.e., the use of behavioral outcome data as a proxy for HIV incidence is very indirect evidence for any change in HIV incidence). Serious inconsistency (i.e., conflicting study results) was a problem in the analyses reporting change in multiple sexual partnerships.

### Limitations of the review

Our review is a comprehensive assessment of the US scientific literature on the effectiveness of CDI. We used comprehensive search strategies in four important bibliographic databases, searched grey literature, examined bibliographies of relevant studies and used rigorous methods throughout our review process. As with any systematic review, however, our review has limitations. While the bibliographic databases we searched had most likely, taken together, indexed all relevant peer-reviewed studies, it is possible that a study was indexed only in a database we did not search. However, this is unlikely. Although we searched a wide range of grey literature sources with the hope to find documents reporting CDI program data from community-based organizations, local public health agencies and other potential CDI implementers, the grey literature itself is nearly inexhaustible and it is conceivable that we could have missed some reports. Similarly, we do not think it is likely that is likely that we missed any such studies.

More serious review limitations arise in the nature of the studies that we found. Very low-quality evidence severely limits our ability to be certain of intervention effects. With most studies relatively old, important social changes and breakthroughs in HIV prevention and treatment since they were conducted could mean that their effects would now be different. Across all included studies, there was little consistency in terms of populations, interventions and outcomes examined. Studies do not examine CDI in all high-risk U.S. populations. Use of composite outcomes by some investigators forced us to exclude some studies.

Most (eight of nine) studies included in this review were non-RCTs and were at high risk of several kinds of biases (S6 File). High risk of bias was an important consideration in our GRADE analyses. With very low quality evidence, an intervention’s true effects may be substantially different from those we have calculated. This applies not only to the size but also to the direction of effect. Critically, nearly all reported outcomes were very indirect, reflecting only sexual risk behaviors. Thus, our confidence in how well these effect estimates translate to actual reduction in risk of HIV transmission is very limited.

We also observed substantial statistical heterogeneity (I^2^ > 75%) in several of the pooled analyses, reflecting variations in study designs, settings, and populations. These also manifested in the form of wider 95% CIs around point estimates. Lastly, we identified substantial variation in the impact of various types of CDI, partially explained as we have shown, by differences in specific content of programs as well as other sources of heterogeneity.

In the process of this review, we also identified seven non-RCT community-based CDI studies that were considered Limited in terms of provision of condoms [38–44]. Overall, evidence from these seven studies was also very low quality and not substantially different from other CDI models in this review (see S5 File).

Our review did not identify any condom distribution programs explicitly targeting sex workers in the US. Only one study approached young and sexually active women in a high-risk community [33]. We offer two plausible explanations for this observation. First, despite its widespread presence, sex work has remained an illegal activity in nearly all of the US, making it an underground industry that is hard to study. Due to criminalization of sex work, there are also very few geographically definable areas or communities in the U.S that lend themselves to community-wide condom distribution programs for sex workers. Secondly, in contrast to the HIV epidemic in southern African and other settings where sex workers are among the most affected key populations, MSM and PWID are the two most affected populations in the US HIV epidemic and are those to which public health authorities pay the most attention.

We did not formally assess risk of publication bias as we did not pool ≥10 studies in any meta-analysis. We identified and extracted data from all comparative studies reporting outcomes in which the relative effect of CDI could be isolated or stratified from other interventions. However, we excluded eight studies from analysis because such data could not be analyzed separately. Had investigators of several studies actually stratified condom distribution outcome data in the presence of other interventions, we could potentially have included additional studies. This problem is not uncommon. O’Reilly and colleagues [45] describe a systematic review of free condom distribution programs that they started but abandoned, even after identifying 34 studies (from the global literature) that apparently met inclusion criteria. O’Reilly reported that it was “not possible to isolate the effect of free condom distribution from other co-occurring interventions” [45].

As we limited our review to US-based studies, our findings are highly applicable to populations residing in the US and reached through community settings. However, most of the included studies were rather old, with all but one [31] published before 2010; many were published in the 1990s, before triple ART regimens came into widespread use, and before pre-exposure prophylaxis (PrEP) became available. It is likely that today’s US populations at high HIV risk (e.g., MSM) might respond differently to CDI, although the direction of this response is yet to be examined. For instance, as a result of increased uptake of HIV testing in recent years, a substantial portion (86%) of HIV infected persons are now aware of their status [3]. Those who are aware of their HIV status may be more likely to adopt safer sexual practices such as serosorting or using condoms with partners whose HIV status is unknown [46, 47]. On the other hand, due to the availability of more effective and simpler ART regimens, people with HIV can now live an almost normal life. Population groups at risk of HIV infection may not perceive HIV as a fatal disease anymore and may engage in risky sexual behavior. Individuals who are on treatment but are not virally suppressed may still transmit the infection to their uninfected sexual or drug-injecting partners. This latter phenomenon may have contributed to the recent resurgence of HIV among certain HIV high-risk groups such as young African-American MSM [48]. Finally, as we report above, condom use at last episode of sex may not fully capture changes in condom use over time in response to an intervention, notwithstanding that it is one of the most frequently reported outcomes [49].

## Conclusions

Given the very low-quality evidence found in our review, we cannot draw firm conclusions about the relative effectiveness of any of the three CDI models (i.e., ongoing, ongoing-plus, and coupon-based) in reducing HIV incidence or risk of HIV infection. Community-based CDI may reduce some risky sexual behaviors, but the evidence for any reduction is limited and of low quality. Lack of biological outcomes precludes assessing the link between community-based CDI and HIV incidence. Rigorous assessment of CDI effectiveness through well-executed studies of appropriate design (e.g., community-based cluster RCTs with adequate duration of follow-up and measurement of HIV biological outcomes) in a range of high-risk populations would provide stronger evidence for nuanced assessments of CDI effectiveness.

## Supporting information

S1 File PRISMA checklist. (PDF)

S2 File Protocol. (PDF)

S3 File Core database search strategies. (PDF)

S4 File Articles screened at the full text level. (PDF)

S5 File Limited CDI studies. (PDF)

S6 File Pooled effect measures and risk of bias tables. (PDF)

S7 File GRADE tables. (PDF)
