# Supplementary material for: Effectiveness of community-based condom distribution interventions to prevent HIV in the United States: A systematic review and meta-analysis
Source: PLoS One. 2017 Aug 3;12(8):e0180718. doi: 10.1371/journal.pone.0180718 (PMC5542551; doi:10.1371/journal.pone.0180718)
Supplement: S2 File — (PDF) [file pone.0180718.s002.pdf]

Study Title: Condom distribution interventions for preventing HIV transmission in the United States: Protocol for a systematic review

Project: HIV-RAMP Model Input Assessment Project

Consortium for the Assessment of Prevention Economics

UCSF: *Mohsen Malekinejad (project lead)*, James G. Kahn (CAPE PI), George Rutherford (co-investigator)**,** Hacsi Horvath (methodologist), Janet Blodgett (data extraction), Andrea Parriott (data quality review & synthesis), Devon McCabe (project manger)*

Centers for Disease Control and Prevention (CDC), Division of HIV/AIDS Prevention (DHAP)
Stephanie Sansom, Paul G Farnham, Emine Yaylali, Timothy Green

***Correspondence:**

Mohsen Malekinejad, MD, DrPH, Institute for Health Policy Studies and Global Health Sciences, University of California San Francisco, 3333 California Street, Suite 265, San Francisco, CA 94118, USA. E-mail address: MMalekinejad@ucsf.edu. Phone: +1 (510) 8168555; Fax: +1 (415) 4760705

This project is supported by Cooperative Agreement (Grant No: U38PS004649) from the US Centers for Disease Control and Prevention (CDC)

University of California San Francisco

Berkeley UNIVERSITY OF CALIFORNIA

STANFORD SCHOOL OF MEDICINE Stanford University Medical Center

GHECon Global Health Economics Consortium Advancing precision healthpolicy

Table of Contents

ABBREVIATIONS **1**

BACKGROUND **2**

RATIONALE FOR SYSTEMATIC REVIEW 3

OBJECTIVES **4**

METHODS: INCLUSION AND EXCLUSION CRITERIA **4**

STUDY DESIGN 4

PICO FRAMEWORK 4

SEARCH METHODS FOR IDENTIFYING STUDIES 6

SCREENING AND DATA COLLECTION 7

DATA EXTRACTION AND MANAGEMENT 7

RISK OF BIAS ASSESSMENT 8

QUALITY OF EVIDENCE 9

ANALYSIS 10

REFERENCES **12**

APPENDIX A – REFINEMENT OF THE INCLUSION & EXCLUSION CRITERIA **14**

APPENDIX B – OUTCOME TABLE **17**

APPENDIX C – RISK OF BIAS ASSESSMENT **18**

ABBREVIATIONS

AIDS	International AIDS Conference
CI	Confidence interval
CROI	Conference on Retroviruses and Opportunistic Infections
IAS	International AIDS Society Conference on HIV Pathogenesis, Treatment and Prevention
ICC	Intra-cluster correlation coefficient
MeSH	National Library of Medicine Medical Subject Headings
MSM	Men who have sex with men
NGO	Non-governmental organization
Non-RCT	Non-randomized controlled trial
PICO	Population, intervention, comparator and outcome
RCT	Randomized controlled trial
RR	Risk ratio
PWID	Persons who inject drugs
US	United States

BACKGROUND

Despite significant progress in preventing HIV infection in the United States (US) and worldwide, far too many people become infected each year. In the US, nearly 50,000 people were newly infected in 2013 (CDC, 2015). High risk groups such as men who have sex with men (MSM) and persons who inject drugs (PWID) were dramatically over-represented in new HIV infections in the US, respectively accounting for 63% and 8% of new infections (CDC, 2015a). Framing the question differently, the risk is much higher for some people in the US population than it is for others. In 2010, 44% of new HIV infections were in people of African-American heritage, despite this population representing only 12% of the overall US population (CDC, 2015a). People of Hispanic or Latino heritage accounted for 21% of new infections in 2010, despite representing 16% of the US population (CDC, 2015a). Because women are much more likely to acquire HIV infection through heterosexual sex than are men, 84% of heterosexually-transmitted new infections in 2010 were in women (CDC, 2015a), though women represented just 20% of new infections overall. The vast majority of new HIV infections occurred through sex without using condoms, also called unprotected sex. Correct and consistent condom use would likely have prevented nearly all new HIV infections in the US last year. It thus remains imperative to develop and implement strategies for increasing condom use in the US. One strategy for increasing condom use is condom distribution.

Condom distribution interventions are usually implemented on one or more of three levels: individual- and group-level, organizational level or at the level of community and society (Charania et al, 2011).

Individual-level interventions for improving condom use are conducted on a one-to-one basis, with the goal of addressing individual knowledge and attitudes about condoms, the skills and behaviors in using them and actually providing condoms to these individuals (Charania et al, 2011). They may also be addressed to couples, i.e. long-term sexual partners (Burton et al, 2010). Group-level interventions are similar in focus but are addressed to couples, or to newly-formed affinity groups (Charania et al, 2011). Organizational interventions work to make bars, clinics, community centers and other organizations (including jails and prisons) responsible for making condoms and prevention messages available and/or accessible to populations coming to those organizations (Charania et al, 2011). Community and social interventions work to change society at a high level. They directly and indirectly address knowledge, attitudes and behaviors around condoms, and often emphasize changing social norms and public attitudes (Charania et al, 2011).

To enhance the effectiveness of condom distribution programs, they can be combined with two types of interventions: 1) structural, and 2) behavioral.

1) Structural interventions attempt to change the “structural” and societal context of condom use, with the goal of reducing individual, organizational and social (including economic) constraints and improving condom availability, acceptability and accessibility (Blankenship et al, 2000). Very often, such interventions include components of free condom distribution, efforts to integrate condom use in community culture and efforts to reduce social barriers in accessing condoms (e.g. embarrassment). Their conceptual framework is grounded more in public health than in individual behavior change (Blankenship et al, 2006). Structural approaches to HIV prevention have included community mobilization interventions (e.g. to improve condom use among sex workers), integrated service delivery (e.g. by co-locating HIV prevention and reproductive health services), contingent funding (e.g. by making receipt of government funding contingent on implementing new laws or policies) and economic and educational interventions (e.g. by improving knowledge or through providing economic empowerment to improve social conditions for individuals or communities) (Blankenship et al, 2006). Social marketing

or social networking interventions addressed to communities are another structural approach to increasing condom use (Wang et al, 2011; Sweat et al, 2012).

2) Behavioral interventions, on the other hand, addressed to individuals, couples, groups and communities

With the aim of reducing sexual risk behavior. Behavioral interventions may be based on a range of theoretical models, but are characterized by measuring at least some outcomes through participant-reported behavior change. Among other possibilities, these interventions may include individual motivational interviewing, cognitive-behavioral group or individual counseling, and couples or group interventions based on social-cognitive theory (CDC, 2015b). Some behavior-change interventions also provide condoms directly to study participants, while others simply promote their use.

RATIONALE FOR SYSTEMATIC REVIEW

1. Structural interventions

Two previous reviews (Charania et al, 2011; Moreno et al, 2014) have only focused on structural interventions for improving condom distribution. The reviews were focused quite differently (see below) and despite the similarity of interventions reviewed, had no overlap among studies and came to different conclusions. While both reviews defined structural interventions in the same way, they defined "community" differently. Charania and colleagues (2011) considered the term in a non-geographic sense; Moreno and colleagues (2014) understood it strictly through a geographic lens. There were several other important differences between the reviews, including the absence of any US-based studies in the Moreno review. The searches for the Charania review (2011) are also quite old by now (performed in September 2007). Because the current review's research questions are different from those of Charania and colleagues (2011), it is necessary not only to update their review but to review again the whole literature before their search date.

In addition, two reviews have explored social networking or social marketing to increase condom use. Wang and colleagues (2011) reviewed social network interventions, and although this would seem to have bearing on changing social norms, the review only assessed individual-level behavioral outcomes.. Sweat and colleagues (2012) reviewed condom social marketing programs, and similarly reported only behavioral outcomes.

2. Behavioral interventions

Several systematic reviews of behavioral interventions for increasing condom use have been published in the past five years (Bailey et al, 2010; Carvalho et al, 2011; Free et al, 2011; Johnson et al, 2011; Lopez et al, 2013; von Sadovszky et al, 2014). Each review approaches the topic from a different standpoint. Bailey and colleagues (2010) assessed the efficacy of interactive computer-based interventions (ICBI) for sexual health promotion, including increased condom use. Carvalho and colleagues (2011) only include randomized controlled trials (RCTs) in women with HIV infection. In Free and colleagues (2011), only RCTs were eligible for inclusion, and the review also assessed pregnancy outcomes. Johnson and colleagues (2011) included only studies conducted in adolescent populations. The review by Lopez and colleagues focuses on increasing condom use for preventing HIV and other sexually transmitted infections (STIs) as well as pregnancy, and also includes only RCTs. Finally, von Szadovszky and colleagues (2014) conducted a "systematic review of systematic reviews" that addressed condom

promotion. This review identified eight systematic reviews conducted between 2002 and 2012, though several of its included reviews did not report statistics for outcomes.

3. Summary

Through this "scoping" examination of the existing literature, it becomes clear that there is a gap. In other words, there is a need for a systematic review of condom distribution interventions in form of structural intervention or combined with behavioral interventions conducted in the US, including both general and high-risk populations and with a range of study designs eligible for inclusion.

OBJECTIVES

To assess the impact of condom distribution interventions (i.e., interventions that tend to make condoms available, acceptable, and accessible and can be combined with other interventions to increase condom use through changing social norms or improve risky sexual behavior) on risk of HIV and STI acquisition and transmission among general populations and populations at high risk of transmitting or acquiring HIV infection in the US.

METHODS: Inclusion and Exclusion criteria

Study Design

The following study designs will be eligible for inclusion

- Randomized controlled trials (RCTs), with randomization at either individual or cluster level
- Non-randomized controlled trials (non-RCTs), with allocation at either individual or cluster level
- Retrospective or prospective observational cohort studies (single or double arm)
- Pre-post (before-after)
- Time series
- Case-control studies
- Serial cross-sectional studies

Criteria for exclusion

- Case reports and case series
- Studies without primary data (e.g., modeling)
- Studies reporting post intervention data only without clear baseline value

PICO framework

We use the population, intervention, comparator and outcome (PICO) schema to outline our inclusion and exclusion criteria.

Population

The following populations will be eligible for inclusion

- Population residing in the US at the time of the study including:
    - General populations
    - Populations at high risk of transmitting or acquiring HIV infection
        - Adolescents (ages 10-19) in low-income settings within the US
        - Homeless people
        - MSM

- High-risk heterosexual populations
- PWID
- Sex workers

If we do not identify US-based studies addressing primary or secondary outcomes, we will consider including studies from other high-income countries as defined by The World Bank (2015).

Interventions

The following types of interventions will be eligible for inclusion

Interventions that aim to increase the availability, accessibility, and acceptability of condoms through a "wide-scale" provision of free (or subsidized) condoms that can be integrated/supplemented with any or all of the following components:

- Social marketing/mass-media campaign to promote condom use (by increasing awareness of condom benefits and normalizing condom use within communities)
- Risk reduction intervention or other prevention interventions that directly or indirectly promote acceptance of condom usage
- Community-wide mobilization efforts to support and encourage condom use
- Change in policy or law to promote condom use (e.g. mandatory condom use in sex workers)
- Individual-, couple-, or group-level behavioral interventions (e.g. counseling, motivational interviewing or approaches based on social-cognitive theory) that aim to change sexual risk behavior and which report outcomes that include increased condom use.

Criteria for exclusion

- Structural interventions for condom promotion combined with other major HIV prevention interventions not indirectly promoting condom use (e.g., adult male circumcision, PrEP, needle exchange).
- Structural interventions for condom promotion combined with other HIV prevention interventions that directly or indirectly promote condom use but also have independent effect on HIV transmission (outside of condom promotion) with results not stratified by intervention components. For example, a structural intervention to improve uptake of HIV testing, but without assessing change in condom accessibility, availability and acceptability; or without showing specifically how condom distribution had impact on objectively measured outcomes.
- Behavioral interventions focused only on changing risky sex behavior but without explicitly providing condoms
- Condom distributions that can not be considered "wide scale," such as giving out condoms one time as "samples," or as incentives for participation in another HIV or STI prevention intervention

Note: We further refined intervention related inclusion/exclusion criteria during the screening process. See **Appendix A** for details.

Comparator

- No intervention.
- A different intervention to improve accessibility, availability or acceptability of condom use.

Outcomes

Primary:

- Change in HIV incidence or prevalence attributable to the intervention
- Clinical or lab-confirmed HIV diagnosis

Secondary:

- Self-reported condom use at last sex.
- STI incidence or prevalence
- Self-reported number of sex partners

For complete list of potential secondary outcomes, please see **Appendix B**. There are many ways in which outcomes of condom use are reported in the literature (e.g. "use at last sex," "always vs. sometimes vs. never vs. ever," "mean number of episodes" etc.). We will extract data for all such variations of self-reported condom use, knowing from experience that studies are not consistent in measuring and reporting these outcomes. We will later standardize the varied reports and analyses by transforming them to a single common "condom use" outcome.

Search methods for identifying studies

Journal and trial databases:

We will search multiple bibliographic databases for primary studies. We will examine other systematic reviews (including those described above) through which we may identify primary studies. Studies published in any language will be eligible for inclusion. We will include all eligible studies regardless of publication status (published, unpublished, in press and in progress).

We will search the following databases for the period from January 1, 1986 to the search date:

- Cochrane Central Register of Controlled Trials
- PubMed
- SCOPUS (includes EMBASE from 1996-current)
- PsycINFO

We selected 1986 as the start of the search frame because this was the year in which CDC first recommended consistent condom use for HIV prevention (CDC, 1986).

We will use appropriate Medical Subject Heading (MeSH) terms and keywords to identify relevant studies. The search strategy will be iterative, in that references of included studies will be searched for additional references. All languages will be included. Our PubMed search strategy will be modified and adapted as needed for use in the other databases. We will improve the sensitivity of our search strategies and build upon them as needed by iteratively updating them with text and key words from relevant studies that were not detected in initial searches.

Conference databases:

We will search conference abstract archives of the International AIDS Conference (AIDS), the International AIDS Society Conference on HIV Pathogenesis, Treatment and Prevention (IAS) and the Conference on Retroviruses and Opportunistic Infections (CROI) for all available abstracts of primary studies and systematic reviews presented at these conferences from their inception dates through 2015. We will also include conference abstracts identified through our search of SCOPUS.

Searching other resources:

In addition to searching electronic databases, we will contact individual researchers, experts working in the field and colleagues at CDC to learn of any relevant studies that may exist in the “grey literature,” or that may be in preparation or in press.

We will also search ClinicalTrials.gov at the National Institutes of Health to identify any ongoing trials.

Screening and data collection

The methodology for data collection and analysis will be based on the guidance of Cochrane Handbook of Systematic Reviews of Interventions (Higgins 2008). Two authors working independently will examine abstracts of all studies identified by electronic or bibliographic scanning. Where necessary, we will obtain the full text to determine the eligibility of reviews for inclusion.

Methods for selection of studies:

One author will perform a broad first cut of all downloaded material from the electronic searches to exclude citations that are plainly irrelevant. Two authors will read the titles, abstracts and descriptor terms of the remaining downloaded citations to identify potentially eligible studies. We will obtain full text copies for all citations identified as potentially eligible, and two authors will independently inspect these to establish the relevance of the study according to the pre-specified inclusion criteria. Where there is uncertainty as to the eligibility of the record, we will obtain and examine the full-text article.

Two authors will independently apply the inclusion criteria to the full-text articles, and any differences arising will be resolved by discussion with a neutral arbiter. We will examine studies for relevance based on intervention, design, types of participants and outcome measures, and will then decide which studies meet inclusion criteria.

Data extraction and management

From all studies meeting inclusion criteria, two authors will independently extract data into a standardized, pre-piloted data extraction form. The following characteristics will be extracted from each included study:

- **Study details:** Complete citation, study location, study design characteristics, funding sources and other relevant details.
- **Study context:** Whether structural components were implemented at individual, organizational, and/or environmental levels; whether interventions were implemented at the individual/group-level, organization-level or community-level; theoretical models underpinning behavioral interventions.
- **Details of participants:** Age range, sex, high-risk group if applicable; socio-cultural and economic characteristics and possible previous exposure to similar interventions.

- **Details of setting:** Contexts of study setting, including background attitudes towards and/or levels of use or condoms as well as other forms of contraception, awareness of HIV/AIDS or STIs, and prevalence of HIV/AIDS or STIs.
- **Outcome details:** Numerators and denominators associated with each outcome; definitions and descriptions of outcomes provided in papers; details of how outcomes were assessed. Duration of exposure involved in the intervention, such as measurements of total time period over which the intervention took place; cost of the intervention per individual or group.
- **Methodologic details:** Recruitment methods, method of randomization if an RCT, numbers of participants entering the study, comparability of groups, study inclusion and exclusion criteria, length of follow-up, losses to follow-up, withdrawals or drop-outs.
- **Bias assessment data:** Other details necessary to perform a bias risk assessment using the Cochrane tool described below.

Risk of bias assessment

Two review authors will independently assess risk of bias for each primary study (both RCTs and observational studies; see Appendix C for further details) using the bias assessment tool described in the Cochrane Handbook (Higgins 2008). We will resolve any disagreement by discussion or by involving a neutral third party to adjudicate. We will generate summary figures to illustrate risk of bias in each study and across all included studies.

The Cochrane approach assesses risk of bias in individual studies across six domains: sequence generation, allocation concealment, blinding, incomplete outcome data, selective outcome reporting and other potential biases.

Sequence generation (checking for selection bias):

- Low risk: investigators described a random component in the sequence generation process, such as the use of random number table, coin tossing, card or envelope shuffling.
- High risk: investigators described a non-random component in the sequence generation process, such as the use of odd or even date of birth, algorithm based on the day or date of birth, hospital or clinic record number. Or: Not randomized at all.
- Unclear risk: insufficient information to permit judgment about the sequence generation process.

Allocation concealment (checking for selection bias):

- Low risk: participants and the investigators enrolling participants cannot foresee assignment (e.g., central allocation; or sequentially numbered, opaque, sealed envelopes).
- High risk: participants and investigators enrolling participants can foresee upcoming assignment (e.g., an open random allocation schedule, a list of random numbers), or envelopes were unsealed, non-opaque or not sequentially numbered. Or: Allocation not concealed at all.
- Unclear risk: insufficient information to permit judgment of the allocation concealment or the method not described.

Blinding (checking for performance bias and detection bias):

- Low risk: blinding of the participants, key study personnel and outcome assessor and unlikely that the blinding could have been broken. Not blinding in the situation where non-blinding is unlikely to introduce bias.

- High risk: no blinding or incomplete blinding when the outcome is likely to be influenced by lack of blinding.
- Unclear risk: insufficient information to permit judgment of adequacy or otherwise of the blinding.

Incomplete outcome data (checking for possible attrition bias through withdrawals, dropouts, protocol deviations):

- Low risk: no missing outcome data, reasons for missing outcome data unlikely to be related to true outcome or missing outcome data balanced in number across groups.
- High risk: reason for missing outcome data likely to be related to true outcome, with either imbalance in number across groups or reasons for missing data.
- Unclear risk: insufficient reporting of attrition or exclusions.

Selective reporting:

- Low risk: a protocol is available, and the primary outcomes in the final trial report correspond closely to those presented in the protocol
- High risk: the primary outcomes differ between the protocol and final trial report.
- Unclear risk: no trial protocol is available or there is insufficient reporting to determine if selective reporting is present.

Other forms of bias:

- Low risk: no evidence of bias from other sources.
- High risk: potential bias from other sources (e.g., early stopping of trial for benefit, fraudulent activity, baseline imbalance between study groups, loss to follow-up ≥20%, no analyses to control for potential confounders).
- Unclear risk: insufficient information to permit judgment of other forms of bias.

For blinding and incomplete outcome data, multiple entries can be made if more than one outcome (or time point) is involved.

Observational studies:

During our bias assessment with the Cochrane instrument (and particularly during our assessment of "other forms of bias") we will make note of any additional methodological issues that would likely increase bias risk. We will look in particular for the following:

- Failure to develop and apply appropriate eligibility criteria (comparability of groups)
- Flawed measurement of both exposure and outcome
- Failure to adequately control confounding
- Incomplete or inadequately short follow-up

Quality of evidence

We will assess the quality of evidence across the literature's body of evidence using the GRADE approach (Guyatt 2011), which defines the quality of evidence for each outcome as "the extent of our confidence that the estimates of effect are correct" (Higgins 2008). The quality rating across studies has four levels: high, moderate, low or very low. Randomized trials are considered to be of high quality but can be downgraded for any of five reasons; similarly, observational studies are considered to be of low quality, but can be upgraded for any of three reasons. The five factors that can decrease the quality of evidence are as follows:

1) risk of bias
2) indirectness of evidence
3) unexplained heterogeneity or inconsistency of results
4) imprecision of results
5) high probability of publication bias

The three factors that can increase the quality level of a body of evidence are as follows:

1) large magnitude of effect
2) plausible confounding would increase confidence in an estimated effect
3) the presence of a dose-response gradient

We will assess the quality of evidence separately for the RCT-based literature and other literature (i.e. non-RCT experiments and observational studies). We will generate GRADE evidence profiles for all outcomes of interest for which data are available.

We may include data from other high-income countries if US-based studies do not address our primary outcomes of interest. In this event (but depending on the context), evidence quality for outcomes substantially informed by evidence from non-US studies may be graded down for indirectness.

Analysis

We will calculate and present summary statistics for the risk ratio (RR) for dichotomous outcomes and the weighted-mean difference for continuous outcomes, using the 95% confidence interval (CI).

We will use the Review Manager 5 software (RevMan 2015) provided by the Cochrane Collaboration for statistical analysis and GRADEpro software (GRADEpro 2008) provided by the GRADE Working Group, to produce GRADE evidence profiles.

If possible, we will calculate summary statistics using meta-analytic methods. Where meta-analysis is not possible or is inappropriate, we will perform a narrative synthesis of results. To summarize evidence quality, we will present findings in GRADE evidence profiles for all outcomes of interest.

Unit of analysis issues:

Depending on the study, the unit of analysis may be clinics, schools, community facilities, or other such venues; or it may be the individual participant. If we identify cluster randomized trials, we will calculate the intra-cluster correlation coefficient (ICC) for each study. The ICC is used to calculate the design-effect which is then used to calculate the effective sample sizes of intervention and control groups (Higgins 2008).

Dealing with missing data:

We will contact study authors if it is necessary to obtain data missing from published reports. If necessary and appropriate, we may impute data.

Assessment of heterogeneity:

We will use the I^2 and the $\boldsymbol{\tau}^2$ statistics to measure heterogeneity among included studies in each analysis. We anticipate substantial heterogeneity across studies, and thus meta-analysis of included studies will be undertaken with caution, if at all. For data points that are relatively homogenous with respect to types of populations, the interventions that are compared, and outcome measures, we will calculate pooled risk ratios (RR) and 95% CI using random effect meta-analysis model. If indeed we find substantial heterogeneity (e.g., I^2 ≥45%) in pooled data from three or more studies, we will attempt to

explore it by pre-specified subgroup analysis. If heterogeneity persists, we will perform sensitivity analyses, present results separately and propose explanations for the observed heterogeneity.

Assessment of reporting biases:

Where we suspect reporting bias we will attempt to contact study authors and ask them to provide missing outcome data. Where this is not possible, and the missing data are thought to introduce serious bias, we will explore the impact of including such studies in the overall assessment of results by a sensitivity analysis.

If any meta-analysis in our review includes 10 more studies, we will assess the potential for publication bias for the studies using a funnel plot (Egger 1997, Higgins 2008). We will attempt to minimize the potential for publication bias through rigorous review methods and by using comprehensive search strategies, including evaluating published and unpublished literature in all languages.

Data synthesis:

We will conduct meta-analysis, if appropriate, using Cochrane's Review Manager software (RevMan 2015). Choice of meta-analysis model is subject to both quantitative and qualitative assessment of heterogeneity. For example, even if heterogeneity is low in pooled data (I^2<45%), we may still use a random effects model because included interventions and populations may still be quite different. If heterogeneity is extremely high (I^2 ≥85%), we may consider not pooling data. If meta-analysis is not scientifically permissible, we will present a narrative synthesis of studies.

Subgroup analysis and investigation of heterogeneity:

In pooled results with high heterogeneity, we will explore heterogeneity through subgroup analyses of the following:

- Country
- High-risk group
- Urban or rural setting
- Region (United States studies)
- Timeframe of studies

Sensitivity analysis:

Where relevant, we will conduct sensitivity analysis to investigate the effect of excluding studies with high risk of bias, studies with arbitrary inclusion criteria and potentially other kinds of studies.

Declarations of interest:

None known.

REFERENCES

1. Adimora AA, Auerbach JD. Structural Interventions for HIV Prevention in the United States. JAIDS Journal of Acquired Immune Deficiency Syndromes 2010; Vol. 55:S132-S135
2. Atienza AA, King AC. Community-based Health Intervention Trials: An Overview of Methodological Issues. Epidemiologic Reviews 2002; Vol. 24, issue 1:72-9.
3. Auerbach J. Transforming social structures and environments to help in HIV prevention. Health Aff (Millwood). 2009/11/06 2009; Vol. 28, issue 6:1655-65.
4. Bailey JV, Murray E, Rait G, Mercer CH, Morris CH, Peacock R, et al. Interactive computer-based interventions for sexual health promotion. Cochrane Database of Systematic Reviews 2010 Sept 9: CD00648
5. Blankenship KM, Bray SJ, Merson MH. Structural interventions in public health. AIDS. 2000;14(Suppl 1):S11–21.
6. Blankenship KM, Friedman SR, Dworkin S, Mantell JE. Structural Interventions: Concepts, Challenges and Opportunities for Research. Journal of Urban Health. 2006;83(1):59-72.
7. Burton J, Darbes LA, Operario D. Couples-focused behavioral interventions for prevention of HIV: Systematic review of the state of evidence. AIDS and Behavior. 2010;14(1):1-10.
8. Carvalho FT, Goncalves TR, Faria ER, Shoveller JA, Piccinini CA, Ramos MC, et al. Behavioral interventions to promote condom use among women living with HIV. Cochrane Database of Systematic Reviews 2011 Sept (9):CD007844.
9. Centers for Disease Control (CDC). Additional recommendations to reduce sexual and drug abuse-related transmission of human T-lymphotropic virus type III/lymphadenopathy-associated virus. MMWR 1986;35:152-5.
10. Centers for Disease Control and Prevention (CDC), 2015.
    a. HIV in the United States: At A Glance. Available from: http://www.cdc.gov/hiv/statistics/basics/ataglance.html [accessed 3 April 2015]
    b. Compendium of Evidence-Based Interventions and Best Practices for HIV Prevention. Available from: http://www.cdc.gov/hiv/prevention/research/compendium/rr/characteristics.html [accessed 23 June 2015]
11. Charania MR, Crepaz N, Guenther-Gray C, Henny K, Liau A, Willis LA, Lyles CM. Efficacy of structural-level condom distribution interventions: a meta-analysis of U.S. and international studies, 1998-2007. AIDS Behav. 2011 Oct;15(7):1283-97.
12. Free C, Roberts IG, Abramsky T, Fitzgerald M, Wensley F. A systematic review of randomised controlled trials of interventions promoting effective condom use. Journal of epidemiology and community health. 2011 Feb;65(2):100-10.
13. GRADEpro [Computer program]. Brozek J, Oxman A, Schünemann. GRADE Working Group, 2008.
14. Gupta GR, Parkhurst JO, Ogden JA, Aggleton P, Mahal A. Structural approaches to HIV prevention. Lancet 2008; Vol. 372; 9640:764-75.

15. Guyatt G, Oxman AD, Akl EA, Kunz R, Vist G, Brozek J, et al. GRADE guidelines: 1. Introduction-GRADE evidence profiles and summary of findings tables. Journal of Clinical Epidemiology 2011 Apr;64(4):383-94.

16. Higgins JPT, Green S (eds). Cochrane Handbook for Systematic Reviews of Interventions. Chichester: Wiley-Blackwell, 2008.

17. Johnson BT, Scott-Sheldon LA, Huedo-Medina TB, Carey MP. Interventions to reduce sexual risk for human immunodeficiency virus in adolescents: A meta-analysis of trials, 1985–2008. Arch Pediatr Adolesc Med 2011, 165(1), 77-84

18. Lopez LM, Otterness C, Chen M, Steiner M, Gallo MF. Behavioral interventions for improving condom use for dual protection. Cochrane Database of Systematic Reviews Oct 2013;10:CD010662.

19. Moreno R, Nababan HY, Ota E, Wariki WM, Ezoe S, Gilmour S, Shibuya K. Structural and community-level interventions for increasing condom use to prevent the transmission of HIV and other sexually transmitted infections. Cochrane Database of Systematic Reviews. 2014 Jul 29;7:CD003363.

20. Review Manager (RevMan), Version 5.3 [Computer program]. Copenhagen: The Nordic Cochrane Centre, The Cochrane Collaboration, 2015.

21. Sweat MD, Denison J, Kennedy C, Tedrow V, O'Reilly K. Effects of condom social marketing on condom use in developing countries: a systematic review and metaanalysis, 1990-2010. Bulletin of the World Health Organization. 2012 Aug;90:613-22.

22. von Sadovszky V, Draudt B, Boch S. A systematic review of reviews of behavioral interventions to promote condom use. Worldviews on evidence-based nursing / Sigma Theta Tau International, Honor Society of Nursing. 2014 Apr;11(2):107-17.

23. Wang K, Brown K, Shen SY, Tucker J. Social network-based interventions to promote condom use: a systematic review. AIDS and Behavior. 2011 Oct;15(7):1298-308.

24. The World Bank. High-income countries. Available from: http://data.worldbank.org/income-level/HIC [accessed 3 April 2015

Appendix A – Refinement of the inclusion & exclusion criteria

1) Distinction between true "Condom Distribution" programs vs. other programs with condom distribution activity

Due to difficulty distinguishing between studies in which condom distribution was an integral component of the intervention, and studies in which condom distribution is a less important component, we developed the following detailed selection criteria to assist with the study screening and selection process.

Inclusion Criteria

A study qualifies for inclusion if it meets both criteria listed below:

- Condom distribution must be included in the main description of the intervention, generally located in the "methods" or "introduction "section. This is a proxy measure of how integral the condom distribution component is to the intervention. Condom distribution activities reported elsewhere (often in the discussion section) without having been mentioned in the "methods" or "introduction" are probably not considered to be important intervention activities. For an example of a study that fails this criterion, see Davey-Rothwell, 2011, where condom distribution is mentioned briefly only in the discussion section.

- Condoms must be available continuously for the entire intervention period/at all intervention sessions; OR the intervention description suggests that a substantial amount of program resources was dedicated to distributing condoms.

Exclusion Criteria

A study should be excluded if it meets any of the criteria below:

- The intervention is described as "brief." Brief interventions do not qualify as ongoing condom distribution interventions. This is a useful indicator for screening out studies at the title and abstract level.

- The main focus of the intervention is to cause behavior change ONLY through changing attitudes, perceptions, skills, or self-efficacy (condom **acceptability**), rather than overcoming barriers related to lack of condom **accessibility** or **affordability**. The main intervention may be described using the terms "skills-training," "motivational," "education" or "educational," or

"counseling." It should be noted that many interventions are multimodal (condom distribution plus behavioral and/or educational components), and we will NOT exclude a study because the intervention described a study component using these terms. For example of an evaluation of a multi factorial intervention that is eligible for inclusion under these criteria, see Fishbein, 1999.

- Condoms are given out only as an "incentive" for participation.

2) Programs with female condom distribution

Initially, this protocol made no distinction between female condoms and male condoms. However, there is a body of literature evaluating interventions that promote female condom use to the exclusion of other sexual risk reduction activities, which may not be appropriate for inclusion in this systematic review. We considered the following types of studies (assuming they meet other criteria), as acceptable for inclusion:

- Studies where both female and male condoms are distributed and promoted, and condom use outcomes measure overall protected (by either type of condom) vs. unprotected sex. We propose to include these studies.

- Studies where both female and male condoms are distributed, but additional program activities, such as education or skills training, focus mainly or exclusively on promotion of female condom use. The outcome measures include overall protected vs. unprotected sex. For an example of this type of study, see Artz, 2000.

- Studies where only female condoms are distributed/promoted, but outcome measures include overall protected v. unprotected sex. The theoretical models behind general condom distribution vs. female condom distribution programs are somewhat different; the latter are largely based on the idea that women's inability to control male condom use is a barrier to consistent condom use, and that overall condom use will increase if women have access to, awareness of, and skills to use female condoms specifically. Consequently, while we plan to include these studies in the systematic review, but not to combine them in meta-analyses with studies where male condoms are distributed.

We excluded the following type of study:

- Only female condoms are distributed/promoted, and only female condom use is measured as an outcome. We plant to exclude these studies, as they do not give a measure of overall protected vs. unprotected sex.

3) Condom distribution for pregnancy prevention

Some studies evaluate programs where condoms are distributed along with other forms of contraceptives, generally with an emphasis on preventing pregnancy, rather than sexually transmitted infections. We will exclude these studies, as they may cause a substitution effect, where persons who had been using condoms primarily as contraception substitute other methods when they are easily available. We also will exclude studies which primarily focus on pregnancy prevention, rather than STI prevention, as they may influence participants to substitute other forms of contraceptives for condoms.

APPENDIX B – Outcome table

Condom use behavior	Biological	Sexual partnership
Condom use - last sex	HIV	# of sex partners, mean
Vaginal intercourse with condoms, mean # episodes	Chlamydia	Any intercourse with non-primary / non-spousal partner / extramarital sex
Unprotected vaginal intercourse, mean # episodes	Hep B	Concurrent partner
Unprotected anal intercourse, mean # episodes	Gonorrhea	
Total unprotected intercourse, mean # episodes	HPV	
Episodes of extramarital sex by husbands not using condoms	Herpes	
Anal intercourse with condoms, mean # episodes	Syphilis	
Total protected intercourse, mean # episodes	Trichomoniasis	
Any unprotected anal sex		
Counts of unprotected anal sex		
Avoided vaginal or anal intercourse without a condom OR knew last sexual partner's HIV test result was negative		
Condom use with any partner - always		
Condom use with any partner - ever		
Condom use with any partner - never or inconsistent		
Condom use sometimes or always		
Condom use with casual partner - always		
Condom use with casual partner - never or inconsistent		
Condom use with regular partner - always		
Condom use with regular partner - never or inconsistent		
Condom use with regular partner - EVER		
Condom use with sex worker - always		
Total number of partner non-condom		
Unprotected Sex - Never		

Appendix C – Risk of Bias Assessment

General notes

- Below, see tables for discussion of criteria for high and low risk of bias for each domain by study design. "Unclear" risk of bias is essentially when there is insufficient reported information to make a judgment of high or low risk.
- Do not assess RCT risk of bias domains for observational studies, or vice versa. Enter N/A in the data extraction sheet. However, keep in mind other kinds of bias risk listed under the four observational domains recommended in the GRADE methodology (comparability of groups, inadequate follow-up, problems with assessment of exposures or outcomes, failure to adequately control for confounding) as "other" sources of biases.

Special notes on observational studies

- Observational studies are inherently at high risk of bias.
- For observational study domains, only assign "high" risk if risk of bias is beyond the normal risks associated with observational study designs. For example, most non-randomized studies have some risk of residual confounding, but they should not be assigned a "high" risk of bias for confounding unless there are additional situations which indicate an exceptionally high probability of confounding.
    - Example: A cohort study in which socioeconomic status is a risk factor for the outcome, and the intervention group had significantly higher socioeconomic status than those not receiving the intervention.
- If there is a high risk of bias **toward null**, rate risk as "high" but note in explanation that bias will likely be towards null. (Later, in GRADE-ing evidence quality, this could potentially increase the quality of evidence, if there are no other "downgrades" in evidence quality for that outcome.)
- We do not want to double-"penalize" studies for the same bias in multiple domains.
- The Cochrane Handbook recommends a four step process to assess risk of bias due to confounders in observational studies:
    1. Write a list of potential predictors of outcome (Cochrane says to do this at the protocol stage)
    2. Identify the confounding factors that the study has identified and those that have been omitted. Note how the potential confounders have been measured.
    3. Assess the balance between comparator groups at baseline with regard to potential confounders
    4. Identify adjustments made for potential confounders such as restriction, matching, stratification, multivariable regression, or propensity scores

Cochrane recommends that step one occurs at the time the protocol is written. For projects where the protocol has already been written and data collection has begun, we should still make a list of important potential confounders to be used in assessing risk of confounding for all studies across a project, and perhaps amend the protocol.

Notes about your judgments

Briefly provide a rationale for your judgments about bias risk in each domain for each study. For most items, say what was done, as the authors describe it. It is OK to copy-paste (in quotes) what they actually say.

Table 1: Risk of Bias domains for RCTs

**Domain**	**Low Risk Criteria**	**High risk criteria**
Randomization (Sequence generation)	The investigators describe a random component in the sequence generation process such as:<br>• Referring to a random number table<br>• Using a computer random number generator<br>• Coin tossing<br>• Shuffling cards or envelopes<br>• Throwing dice<br>• Drawing of lots<br>• Minimization (minimization may be implemented without a random element, and this is considered to be equivalent to being random).	The investigators describe a non-random component in the sequence generation process, for example:<br>• Sequence generated by odd or even date of birth<br>• Sequence generated by some rule based on date (or day) of admission<br>• Sequence generated by some rule based on hospital or clinic record number.
Allocation concealment	Participants and investigators enrolling participants could not foresee assignment because one of the following, or an equivalent method, was used to conceal allocation:	Participants or investigators enrolling participants could possibly foresee assignments and thus introduce selection bias, such as allocation based on:<br>• Using an open random allocation

	• Central allocation (phone, web-based, pharmacy-controlled)<br>• Sequentially numbered drug containers of identical appearance<br>• Sequentially numbered, opaque, sealed envelopes.	schedule (e.g. a list of random numbers)<br>• Assignment envelopes were used without appropriate safeguards (e.g. if envelopes were unsealed or non-opaque or not sequentially numbered)<br>• Alternation or rotation<br>• Date of birth<br>• Case record number<br>• Any other explicitly unconcealed procedure.
Blinding of participants and personnel	Any one of the following:<br>• No blinding or incomplete blinding, but the review authors judge that the outcome is not likely to be influenced by lack of blinding;<br>• Blinding of participants and key study personnel ensured, and unlikely that the blinding could have been broken.	Any one of the following:<br>• No blinding or incomplete blinding, and the outcome is likely to be influenced by lack of blinding;<br>• Blinding of key study participants and personnel attempted, but likely that the blinding could have been broken, and the outcome is likely to be influenced by lack of blinding.
Blinding of outcome assessors (the person who is in charge of outcome measurement – if self-reported outcome	Any one of the following:<br>• No blinding of outcome assessment, but the review authors judge that the outcome	Any one of the following:<br>• No blinding of outcome assessment, and the outcome measurement is likely to be

using self-administrated survey, then participant is outcome assessor)	measurement is not likely to be influenced by lack of blinding<br>• Blinding of outcome assessment ensured, and unlikely that the blinding could have been broken.	influenced by lack of blinding<br>• Blinding of outcome assessment, but likely that the blinding could have been broken, and the outcome measurement is likely to be influenced by lack of blinding.

Table 2: Risk of Bias for all studies

Domain	Low Risk Criteria	High Risk Criteria
Incomplete outcome data	Any one of the following:<br>• No missing outcome data<br>• Reasons for missing outcome data unlikely to be related to true outcome (for survival data, censoring unlikely to be introducing bias)<br>• Missing outcome data balanced in numbers across intervention groups, with similar reasons for missing data across groups<br>• For dichotomous outcome data, the proportion of missing outcomes compared with observed event risk not enough to have a clinically relevant	Any one of the following:<br>• Reason for missing outcome data likely to be related to true outcome, with either imbalance in numbers or reasons for missing data across intervention groups<br>• For dichotomous outcome data, the proportion of missing outcomes compared with observed event risk enough to induce clinically relevant bias in intervention effect estimate<br>• For continuous outcome data, plausible effect size (difference in means or standardized difference in means) among missing outcomes enough to induce

	impact on the intervention effect estimate<br>• For continuous outcome data, plausible effect size (difference in means or standardized difference in means) among missing outcomes not enough to have a clinically relevant impact on observed effect size<br>• Missing data have been imputed using appropriate methods.<br>• Appropriate non-response weighting methods have been used	clinically relevant bias in observed effect size<br>• 'As-treated' analysis done with substantial departure of the intervention received from that assigned at randomization<br>• Potentially inappropriate application of simple imputation.
Selective outcome reporting*	Any of the following:<br>• The study protocol is available and all of the study's pre-specified (primary and secondary) outcomes that are of interest in the review have been reported in the pre-specified way<br>• The study protocol is not available but it is clear that the published reports include all expected outcomes, including those that were pre-specified (convincing text of this nature may be uncommon).	Any one of the following:<br>• Not all of the study's pre-specified (in protocol or methods) primary outcomes have been reported<br>• One or more primary outcomes is reported using measurements, analysis methods or subsets of the data (e.g. subscales) that were not pre-specified<br>• One or more reported primary outcomes were not pre-specified (unless clear justification for their reporting is provided, such as an unexpected adverse effect)<br>• One or more outcomes of interest in the review are reported

		incompletely so that they cannot be entered in a meta-analysis<br>• The study report fails to include results for a key outcome that would be expected to have been reported for such a study.<br>• Reporting of proximal outcomes if primary outcomes could have been measured
Other risk of bias	No other source of bias identified	Other problems with studies, for example:<br>• Early stopping for benefit<br>• Contamination<br>• Changes to study protocol during study<br>• Number of participants in each arm not reported<br>• Randomized drop-outs replaced by non-randomized participants<br>• Industry-funded authors apparently making "too strong a case"<br>• Emphasis on significant secondary outcomes in the presence on non-significant primary outcomes<br>• Secular trends in single arm pre-post studies<br>• Inappropriate statistical techniques<br>• Bias covered in "observational"

		domains, when rating an RCT

*Will often be “unclear” if no protocol exists or is available (common with observational studies)

Table #3: Risk of Bias for observational studies

Domain	Low Risk Criteria	High Risk Criteria
Comparability of groups	Major differences between baseline intervention and control (or case and control) groups are due to differences in the groups in the source population.<br>• Same recruitment method and consenting process across groups<br>• Control series is representative of the source population for the case series<br>• Adjustment for baseline differences in groups	High probability of major differences in baseline intervention and control (or case and control) groups that are not present in the source population. May be due to:<br>• Different eligibility criteria across groups<br>• Different consenting methods across groups<br>• Different recruitment settings (cohort or two multiple cross-sectional studies)<br>• Different recruiting methods<br>• Hospital or other institution based control series (case-control studies)*<br>• Post-test only study with a single intervention or control "cluster" (city, state, school district, etc.)
Problems with assessment of exposures or outcomes	Low potential for misclassification of outcome or exposure in any group<br>• Use of highly objective exposure or outcome assessments, like objective laboratory tests<br>Or	Any of the following:<br>• Differences in measurement of exposure between outcome groups (e.g. potential for recall bias in case control studies)<br>• Differential surveillance for outcome in exposed and

	Dichotomous outcome and exposure are measured the same for all study participants and specificity is very high.	unexposed<br>• Test for outcome likely has different sensitivity and specificity in intervention and control groups<br>• Low specificity, even if non-differential (bias toward null)<br>• Low sensitivity or specificity of non-binary outcome, even if non-differential
Confounding	One of the following:<br>• Major potential confounders are evenly balanced across intervention and control groups (or unlikely to be associated with intervention)<br>• Studies include adjustment for baseline values of the outcome, and time between baseline and follow-up is short (can adjust for unmeasured confounders)<br>• Appropriate methods are used to adjust for major potential confounders (restriction, stratification, instrumental variables, matching, propensity scores, multivariable regression	High probability that at least one potential confounder is associated with the intervention status, and any of the following:<br>• Potential confounder is not measured<br>• Lack of appropriate statistical adjustment for potential confounder<br>• High potential of serious misclassification of potential confounder

Inadequate follow-up	Follow-up is adequate to observe outcomes, and follow-up is the same for all groups, including lead-time	One of the following:<br>• Differential follow-up times between intervention and control groups<br>• Inadequate follow-up time to observe outcome (bias toward null)<br>• Failure to account for lead time in screening studies

* Where institution is not true source population for cases

** See section above on Cochrane recommended procedure to assess confounding. List of potential confounders should be pre-specified.
