# Supplementary material for: Effectiveness of community-based condom distribution interventions to prevent HIV in the United States: A systematic review and meta-analysis
Source: PLoS One. 2017 Aug 3;12(8):e0180718. doi: 10.1371/journal.pone.0180718 (PMC5542551; doi:10.1371/journal.pone.0180718)
Supplement: S3 File — (PDF) [file pone.0180718.s003.pdf]

S3: SEARCH STRATEGIES

We conducted searches on 23 June 2015 from earliest records to the search date. We updated our searches on 17 April 2017 to capture all relevant studies published after the 2015 search date.

Search	PubMed Query
**#5**	**#1 AND #2 AND #3 AND #4**
#4	Search (Condom*[tiab] OR condom distribution[tiab] OR condom promotion[tiab] OR condom acceptability[tiab] OR condom availability[tiab] OR condom accessibility[tiab] OR Condoms/organization and administration[mh] OR Condoms/utilization[mh] OR Condoms/supply and distribution[mh]) OR (HIV*[ti] AND prevent*[ti])
#3	Search sexually transmitted[tiab] OR sexually transmissible[tiab] OR HIV*[tiab] OR HIV Infections/prevention and control[mh] OR AIDS[tiab] OR human immunodeficiency[tiab] OR human immune deficiency[tiab] OR Sexually Transmitted Diseases/prevention and control[mh]
#2	Search randomized controlled trial[pt] OR controlled clinical trial[pt] OR randomized controlled trials[mh] OR random allocation[mh] OR random*[tiab] OR trial*[tiab] OR prospective studies[mh] OR intervention*[tiab] OR case-control[tiab] OR cross-section*[tiab] OR observational[tiab] OR Cohort studies[mh] OR Program evaluation[mh] OR non-random*[tiab] OR nonrandom*[tiab] OR "before and after"[tiab] OR "time series"[tiab] OR cohort*[tiab] OR intervention*[tiab] OR prospective*[tiab] OR cluster*[tiab] OR longitud*[tiab] OR systematic review*[ti] OR meta-analysis[ti] OR metaanalysis[ti] OR (systematic*[tiab] AND review*[tiab]) OR systemic review[tiab] OR Meta-analysis[pt]
#1	Search (((availab*[tiab] OR accessib*[tiab] OR acceptab*[tiab]) AND (structur*[tiab] AND HIV*[tiab]))) OR (structur*[tiab] OR community[tiab] OR distribut*[tiab] OR market*[tiab] OR provid*[tiab] OR provision[tiab] OR promot*[tiab] OR dispers*[tiab] OR dispens*[tiab] OR subsidis*[tiab] OR subsidiz*[tiab] OR social marketing[tiab] OR mass media[tiab] OR condom availability[tiab] OR behav*[tiab] OR social norms[tiab] OR free[tiab] OR legislat*[tiab] OR law[tiab] OR legal[tiab] OR Health Promotion/organization and administration[mh] OR Health Promotion/supply and distribution[mh] OR Health Promotion/economics[mh] OR Health Promotion/methods[mh] OR Community Health Planning[mh] OR Social Marketing[mh])

Embase

1. 'human immunodeficiency virus' OR HIV* OR 'sexually transmitted disease' OR sexually
2. condom OR condoms
3. acceptab* OR availab* OR accessib* OR distrib* OR promot* OR dispens* OR subsidi* OR ‘social marketing’
4. #1 AND #2 AND #3

Cochrane Central Register of Controlled Trials

1. “human immunodeficiency virus” OR HIV* OR “sexually transmitted”
2. condom OR condoms
3. acceptab* OR availab* OR accessib* OR distrib* OR promot* OR dispens* OR subsidi* OR “social marketing”
4. #1 AND #2 AND #3

PsycINFO

1. “human immunodeficiency virus” OR HIV* OR “sexually transmitted”
2. condom OR condoms
3. acceptab* OR availab* OR accessib* OR distrib* OR promot* OR dispens* OR subsidi* OR “social marketing”

#1 AND #2 AND #3
