# Supplementary material for: Effectiveness of community-based condom distribution interventions to prevent HIV in the United States: A systematic review and meta-analysis
Source: PLoS One. 2017 Aug 3;12(8):e0180718. doi: 10.1371/journal.pone.0180718 (PMC5542551; doi:10.1371/journal.pone.0180718)
Supplement: S4 File — (PDF) [file pone.0180718.s004.pdf]

**S4: Articles screened at the full text level**
**Systematic Review of Community-based Condom Distribution Interventions in the US: UCSF CAPE Project**

## Contents


Included: Community-based condom distribution intervention (k=9) ........ 1

Included: Limited condom distribution intervention (k=7) ........ 2

Included: School-based condom distribution intervention (k=6) ........ 2

Excluded: Intervention other than condom distribution (k=148) ........ 4

Excluded: Condom distribution not integral to the intervention (k=38) ........ 15

Excluded: Multi-faceted CDI but outcome data not stratified (k=8) ........ 18

Excluded: Outcomes of interest not reported (k=12) ........ 19

Excluded: Insufficient quantitative information (k=2) ........ 19

Excluded: Other reasons (e.g., reviews, non-US studies) (k=39) ........ 20


## Included: Community-based condom distribution intervention (k=9)


Alstead, M., Campsmith, M., Halley, C. S., Hartfield, K., Goldbaum, G., & Wood, R. W. (1999). Developing, implementing, and evaluating a condom promotion program targeting sexually active adolescents. *AIDS Education and Prevention, 11*(6), 497-512.

Bull, S. S., Posner, S. F., Ortiz, C., Beaty, B., Benton, K., Lin, L., . . . Evans, T. (2008). POWER for reproductive health: results from a social marketing campaign promoting female and male condoms. *J Adolesc Health, 43*(1), 71-78. doi:10.1016/j.jadohealth.2007.12.009

Calsyn, D. A., Meinecke, C., Saxon, A. J., & Stanton, V. (1992). Risk reduction in sexual behavior: A condom giveaway program in a drug abuse treatment clinic. *American Journal of Public Health, 82*(11), 1536-1538.

Cohen, D. A., Dent, C., MacKinnon, D., & Hahn, G. (1992). Condoms for men, not women. Results of brief promotion programs. *Sex Transm Dis, 19*, 245-251.

Cohen, D. A., Farley, T. A., Bedimo-Etame, J. R., Scribner, R., Ward, W., Kendall, C., & Rice, J. (1999). Implementation of condom social marketing in Louisiana, 1993 to 1996. *American Journal of Public Health, 89*, 204-208. doi:http://dx.doi.org/10.2105/AJPH.89.2.204

Eisenberg, M. E., Hannan, P. J., Lust, K. A., Lechner, K. E., Garcia, C., & Frerich, E. A. (2013). Sexual health resources at Minnesota colleges: associations with students' sexual health behaviors. *Perspect Sex Reprod Health, 45*(3), 132-138. doi:10.1363/4513213

Lauby, J. L., Smith, P. J., Stark, M., Person, B., & Adams, J. (2000). A community-level HIV prevention intervention for inner-city women: Results of the women and infants demonstration projects. *American Journal of Public Health, 90*, 216-222. doi:http://dx.doi.org/10.2105/AJPH.90.2.216

Ross, M. W., Chatterjee, N. S., & Leonard, L. (2004). A community level syphilis prevention programme: outcome data from a controlled trial. *Sex Transm Infect, 80*, 100-104.

Sellers, D. E., McGraw, S. A., & McKinlay, J. B. (1994). Does the promotion and distribution of condoms increase teen sexual activity? Evidence from an HIV prevention program for Latino youth. *Am J Public Health, 84*, 1952-1959.

## Included: Limited condom distribution intervention (k=7)

Artz, L., Macaluso, M., Brill, I., Kelaghan, J., Austin, H., Fleenor, M., . . . Hook 3rd, E. (2000). Effectiveness of an intervention promoting the female condom to patients at sexually transmitted disease clinics. *American Journal of Public Health, 90*(2), 237.

Choi, K. H., Hoff, C., Gregorich, S. E., Grinstead, O., Gomez, C., & Hussey, W. (2008). The efficacy of female condom skills training in HIV risk reduction among women: a randomized controlled trial. *Am J Public Health, 98*(10), 1841-1848. doi:10.2105/ajph.2007.113050

Collins, C., Kohler, C., Diclemente, R., & Wang, M. Q. (1999). Evaluation of the exposure effects of a theory-based street outreach HIV intervention on African-American drug users. *Eval Program Plann, 22*, 279-293.

Fishbein, M., Higgins, D., Rietmeijer, C., & Wolitski, R. (1999). Community-level HIV intervention in 5 cities: final outcome data from the CDC AIDS Community Demonstration Projects. *American Journal of Public Health, 89*(3), 336.

Kegeles, S. M., Hays, R. B., & Coates, T. J. (1996). The Mpowerment Project: a community-level HIV prevention intervention for young gay men. *Amercian Journal of Public Health, 86*(8), 1129-1136. Retrieved from http://onlinelibrary.wiley.com/o/cochrane/clcentral/articles/441/CN-00635441/frame.html

Watters, J. K., Downing, M., Case, P., Lorvick, J., Cheng, Y. T., & Fergusson, B. (1990). AIDS prevention for intravenous drug users in the community: street-based education and risk behavior. *Am J Community Psychol, 18*, 587-596.

Wendell, D. A., Cohen, D. A., LeSage, D., & Farley, T. A. (2003). Street outreach for HIV prevention: effectiveness of a state-wide programme. *Int J STD AIDS, 14*, 334-340. doi:10.1258/095646203321605549

## Included: School-based condom distribution intervention (k=6)

Blake, S. M., Ledsky, R., Goodenow, C., Sawyer, R., Lohrmann, D., & Windsor, R. (2003). Condom Availability Programs in Massachusetts High Schools: Relationships with Condom Use and Sexual Behavior. *American Journal of Public Health, 93*(6), 955-962.

Furstenberg Jr, F. F., Geitz, L. M., Teitler, J. O., & Weiss, C. C. (1997). Does condom availability make a difference? An evaluation of Philadelphia's health resource centers. *Fam Plann Perspect*, 123-127.

Guttmacher, S., Lieberman, L., Ward, D., Freudenberg, N., Radosh, A., & Des Jarlais, D. (1997). Condom availability in New York City public high schools: relationships to condom use and sexual behavior. *Am J Public Health, 87*, 1427-1433.

Kirby, D., Brener, N. D., Brown, N. L., Peterfreund, N., Hillard, P., & Harrist, R. (1999). The impact of condom distribution in Seattle schools on sexual behavior and condom use. *American Journal of Public Health, 89*(2), 182-187.

Schuster, M. A., Bell, R. M., Berry, S. H., & Kanouse, D. E. (1998). Impact of a high school condom availability program on sexual attitudes and behaviors. *Fam Plann Perspect, 30*, 67-72, 88.

Wretzel, S. R., Visintainer, P. F., & Pinkston Koenigs, L. M. (2011). Condom availability program in an inner city public school: effect on the rates of gonorrhea and chlamydia infection. *J Adolesc Health, 49*(3), 324-326. doi:10.1016/j.jadohealth.2010.12.011

**Excluded: Intervention other than condom distribution (k=148)**

Anonymous. (2005). A gender and culture-specific HIV prevention programme significantly reduces risky sexual behaviours in African American adolescent girls. *Evidence-Based Healthcare and Public Health, 9*(1), 67-68. doi:10.1016/j.ehbc.2004.11.010

Aronson, R. E., Rulison, K. L., Graham, L. F., Pulliam, R. M., McGee, W. L., Labban, J. D., . . . Rhodes, S. D. (2013). Brothers Leading Healthy Lives: Outcomes from the pilot testing of a culturally and contextually congruent HIV prevention intervention for black male college students. *AIDS Educ Prev, 25*(5), 376-393. doi:10.1521/aeap.2013.25.5.376

Bachmann, L. H., Grimley, D. M., Chen, H., Aban, I., Hu, J., Zhang, S., . . . Hook, E. W., 3rd. (2009). Risk behaviours in HIV-positive men who have sex with men participating in an intervention in a primary care setting. *Int J STD AIDS, 20*(9), 607-612. doi:10.1258/ijsa.2009.009030

Baker, A., Heather, N., Wodak, A., Dixon, J., & Holt, P. (1993). Evaluation of a cognitive-behavioural intervention for HIV prevention among injecting drug users. *AIDS (London, England), 7*(2), 247-256.

Baker, S. A., Beadnell, B., Stoner, S., Morrison, D. M., Gordon, J., Collier, C., . . . Stielstra, S. (2003). Skills training versus health education to prevent STDs/HIV in heterosexual women: a randomized controlled trial utilizing biological outcomes. *AIDS Education and Prevention, 15*(1), 1-14.

Basen-Engquist, K., Coyle, K. K., Parcel, G. S., Kirby, D., Banspach, S. W., Carvajal, S. C., & Baumler, E. (2001). Schoolwide effects of a multicomponent HIV, STD, and pregnancy prevention program for high school students. *Health Education & Behavior, 28*(2), 166-185.

Berkman, A., Pilowsky, D. J., Zybert, P. A., Herman, D. B., Conover, S., Lemelle, S., . . . Susser, E. (2007). HIV prevention with severely mentally ill men: a randomised controlled trial. *AIDS Care, 19*(5), 579-588. doi:10.1080/09540120701213989

Bolu, O. O., Lindsey, C., Kamb, M. L., Kent, C., Zenilman, J., Douglas, J. M., . . . Peterman, T. A. (2004). Is HIV/sexually transmitted disease prevention counseling effective among vulnerable populations?: a subset analysis of data collected for a randomized, controlled trial evaluating counseling efficacy (Project RESPECT). *Sex Transm Dis, 31*(8), 469-474.

Branson, B. M., Peterman, T. A., Cannon, R. O., Ransom, R., & Zaidi, A. A. (1998). Group counseling to prevent sexually transmitted disease and HIV: a randomized controlled trial. *Sex Transm Dis, 25*(10), 553-560.

Brodie, D. A., & Gant, L. M. (2006). Learning the Makeda Mindset: An afrocentric-inspired HIV prevention intervention for African American heterosexual women. *American Public Health Association 134th Annual Meeting & Exposition; Nov 4 2006; Boston,MA*.

Brown, L. K., Schultz, J. R., Parsons, J. T., Butler, R. B., Forsberg, A. D., Kocik, S. M., . . . Aledort, L. (2000). Sexual behavior change among human immunodeficiency virus-infected adolescents with hemophilia. Adolescent Hemophilia Behavioral Intervention Evaluation Project Study Group. *Pediatrics, 106*, E22.

Bryan, A. D., Schmiege, S. J., & Broaddus, M. R. (2009). HIV risk reduction among detained adolescents: a randomized, controlled trial. *Pediatrics, 124*(6), e1180-1188. doi:10.1542/peds.2009-0679

Butler, R. B., Schultz, J. R., Forsberg, A. D., Brown, L. K., Parsons, J. T., King, G., . . . Manco-Johnson, M. (2003). Promoting safer sex among HIV-positive youth with haemophilia: theory, intervention, and outcome. *Haemophilia, 9*, 214-222.

Butts, J. B., & Hartman, S. (2002). Project BART: Effectiveness of a behavioral intervention to reduce HIV risk in adolescents. *MCN: The American Journal of Maternal/Child Nursing, 27*, 163-169. doi:http://dx.doi.org/10.1097/00005721-200205000-00007

Calsyn, D. A., Burlew, A. K., Hatch-Maillette, M. A., Beadnell, B., Wright, L., & Wilson, J. (2013). An HIV prevention intervention for ethnically diverse men in substance abuse treatment: pilot study findings. *Am J Public Health, 103*(5), 896-902. doi:10.2105/ajph.2012.300970

Calsyn, D. A., Saxon, A. J., Wells, E. A., & Greenberg, D. M. (1992). Longitudinal sexual behavior changes in injecting drug users. *AIDS, 6*, 1207-1211.

Carey, M. P., Braaten, L. S., Maisto, S. A., Gleason, J. R., Forsyth, A. D., Durant, L. E., & Jaworski, B. C. (2000). Using information, motivational enhancement, and skills training to reduce the risk of HIV infection for low-income urban women: A second randomized clinical trial. *Health Psychology, 19*, 3-11. doi:http://dx.doi.org/10.1037/0278-6133.19.1.3

Carey, M. P., Carey, K. B., Maisto, S. A., Gordon, C. M., Schroder, K. E. E., & Vanable, P. A. (2004). Reducing HIV-Risk Behavior Among Adults Receiving Outpatient Psychiatric Treatment: Results From a Randomized Controlled Trial. *Journal of Consulting and Clinical Psychology, 72*, 252-268. doi:http://dx.doi.org/10.1037/0022-006X.72.2.252

Carmona, J., Slesnick, N., Guo, X., & Letcher, A. (2014). Reducing High Risk Behaviors among Street Living Youth: Outcomes of an Integrated Prevention Intervention. *Child Youth Serv Rev, 43*, 118-123. doi:10.1016/j.childyouth.2014.05.015

Caron, F., Godin, G., Otis, J., & Lambert, L. D. (2004). Evaluation of a theoretically based AIDS/STD peer education program on postponing sexual intercourse and on condom use among adolescents attending high school. *Health education research, 19*(2), 185-197. doi:10.1093/her/cyg017

Chesniak-Phipps, L. M. (2002). *Examining the factors that influence sexual activity and condom use among African American youth.* (AAI3049651).

Chesson, H. W., Greenberg, J. B., & Hennessy, M. (2002). The cost-effectiveness of the WINGS intervention: a program to prevent HIV and sexually transmitted diseases among high-risk urban women (Structured abstract). *BMC Infectious Diseases, 2*(1), 24.

Cohn, D., Rietmeijer, C., Kane, M., Cooper, S., Martindale, C., & Judson, F. (1992). Changes in sexual behavior and condom use associated with a risk-reduction program--Denver 1988-1991. *MMWR Morb Mortal Wkly Rep, 41*(23), 412-415.

Collins, P., Susser, E., Valencia, E., Miller, S., Saez, H., & Geller, P. (1996). HIV Prevention for Mentally Ill Men and Women. *Xth World Congress of Psychiatry, Madrid, Spain. 23rd-28th August, 1996.*

Compton, W. M., Cottler, L. B., Ben-Abdallah, A., Cunningham-Williams, R., & Spitznagel, E. L. (2000). The effects of psychiatric comorbidity on response to an HIV prevention intervention. *Drug and Alcohol Dependence, 58*(3), 247-257.

Cottler, L. B., Compton, W. M., Ben Abdallah, A., Cunningham-Williams, R., Abram, F., Fichtenbaum, C., & Dotson, W. (1998). Peer-delivered interventions reduce HIV risk behaviors among out-of-treatment drug abusers. *Public Health Rep, 113 Suppl 1*, 31-41.

Cottler, L. B., Leukefeld, C., Hoffman, J., Desmond, D., Wechsberg, W., Inciardi, J. A., . . . Woodson, S. (1998). Effectiveness of HIV risk reduction initiatives among out-of-treatment non-injection drug users. *J Psychoactive Drugs, 30*, 279-290. doi:http://dx.doi.org/10.1080/02791072.1998.10399703

Coyle, K., Basen-Engquist, K., Kirby, D., Parcel, G., Banspach, S., Collins, J., . . . Harrist, R. (2002). Safer choices: reducing teen pregnancy, HIV, and STDs. *Public Health Rep, 116 Suppl 1*, 82-93.

Coyle, K., Basen-Engquist, K., Kirby, D., Parcel, G., Banspach, S., Harrist, R., . . . Weil, M. (1999). Short-term impact of safer choices: a multicomponent, school-based HIV, other STD, and pregnancy prevention program. *Journal of school health, 69*(5), 181-188.

Coyle, K. K., Kirby, D. B., Robin, L. E., Banspach, S. W., Baumler, E., & Glassman, J. R. (2006). All4You! A randomized trial of an HIV, other STDs, and pregnancy prevention intervention for alternative school students. *AIDS Education and Prevention, 18*(3), 187-203. doi:10.1521/aeap.2006.18.3.187

Dancy, B. L., Marcantonio, R., & Norr, K. (2000). The long-term effectiveness of an HIV prevention intervention for low-income African American women. *AIDS Education and Prevention, 12*(2), 113-125.

Darrow, W. W., & Biersteker, S. (2008). Short-term impact evaluation of a social marketing campaign to prevent syphilis among men who have sex with men. *American Journal of Public Health, 98*(2), 337-343. doi:10.2105/AJPH.2006.109413

DiClemente, R. J., Brown, J. L., Sales, J. M., & Rose, E. S. (2013). Rate of decay in proportion of condom-protected sex acts among adolescents after participation in an HIV risk-reduction intervention. *J Acquir Immune Defic Syndr, 63 Suppl 1*, S85-89. doi:10.1097/QAI.0b013e3182920173

DiClemente, R. J., & Wingood, G. M. (1995). A randomized controlled trial of an HIV sexual risk-reduction intervention for young African-American women. *JAMA, 274*, 1271-1276.

DiClemente, R. J., Wingood, G. M., Harrington, K. F., Lang, D. L., Davies, S. L., Hook III, E. W., . . . Gordon, A. B. (2004). Efficacy of an HIV prevention intervention for African American adolescent girls: a randomized controlled trial. *JAMA, 292*(2), 171-179.

DiIorio, C., McCarty, F., Resnicow, K., Lehr, S., & Denzmore, P. (2007). REAL men: a group-randomized trial of an HIV prevention intervention for adolescent boys. *American Journal of Public Health, 97*(6), 1084-1089. doi:10.2105/AJPH.2005.073411

Dolcini, M. M., Harper, G. W., Boyer, C. B., & Pollack, L. M. (2010). Project ORE: A friendship-based intervention to prevent HIV/STI in urban African American adolescent females. *Health Educ Behav, 37*(1), 115-132. doi:10.1177/1090198109333280

Echenique, M., Illa, L., Saint-Jean, G., Avellaneda, V. B., Sanchez-Martinez, M., & Eisdorfer, C. (2013). Impact of a secondary prevention intervention among HIV-positive older women. *AIDS Care - Psychological and Socio-Medical Aspects of AIDS/HIV, 25*(4), 443-446. doi:http://dx.doi.org/10.1080/09540121.2012.712666

El-Bassel, N., Gilbert, L., Wu, E., Witte, S. S., Chang, M., Hill, J., & Remien, R. H. (2011). Couple-based HIV prevention for low-income drug users from New York City: a randomized controlled trial to reduce dual risks. *J Acquir Immune Defic Syndr, 58*(2), 198-206. doi:10.1097/QAI.0b013e318229eab1

El-Bassel, N., Jemmott, J. B., Landis, J. R., Pequegnat, W., Wingood, G. M., Wyatt, G. E., & Bellamy, S. L. (2010). National Institute of Mental Health Multisite Eban HIV/STD Prevention Intervention for African American HIV Serodiscordant Couples: a cluster randomized trial. *Archives of internal medicine, 170*(17), 1594-1601. doi:10.1001/archinternmed.2010.261

el-Bassel, N., & Schilling, R. F. (1992). 15-month followup of women methadone patients taught skills to reduce heterosexual HIV transmission. *Public Health Rep, 107*, 500-504.

Eldridge, G. D., St Lawrence, J. S., Little, C. E., Shelby, M. C., Brasfield, T. L., Service, J. W., & Sly, K. (1997). Evaluation of the HIV risk reduction intervention for women entering inpatient substance abuse treatment. *AIDS Educ Prev, 9*, 62-76.

Ellen, J. M., Greenberg, L., Willard, N., Korelitz, J., Kapogiannis, B. G., Monte, D., . . . Gonin, R. (2015). Evaluation of the effect of human immunodeficiency virus-related structural interventions: the connect to protect project. *JAMA Pediatr, 169*(3), 256-263. doi:10.1001/jamapediatrics.2014.3010

Exner, T. M., Mantell, J. E., Hoffman, S., Adams-Skinner, J., Stein, Z. A., & Leu, C. S. (2011). Project REACH: a provider-delivered dual protection intervention for women using family planning services in New York City. *AIDS Care, 23*(4), 467-475. doi:10.1080/09540121.2010.516335

Fishbein, M., Hennessy, M., Kamb, M., Bolan, G. A., Hoxworth, T., Iatesta, M., . . . Zenilman, J. M. (2001). Using intervention theory to model factors influencing behavior change: Project RESPECT. *Evaluation & the Health Professions, 24*, 363-384. doi:http://dx.doi.org/10.1177/01632780122034966

Fisher, J. D., Fisher, W. A., Misovich, S. J., Kimble, D. L., & Malloy, T. E. (1996). Changing AIDS risk behavior: Effects of an intervention emphasizing AIDS risk reduction information, motivation, and behavioral skills in a college student population. *Health Psychology, 15*, 114-123. doi:http://dx.doi.org/10.1037/0278-6133.15.2.114

Fogarty, L. A., Heilig, C. M., Armstrong, K., Cabral, R., Galavotti, C., Gielen, A. C., & Green, B. M. (2001). Long-term effectiveness of a peer-based intervention to promote condom and contraceptive use among HIV-positive and at-risk women. *Public Health Rep, 116 Suppl 1*, 103-119.

Gaydos, C. A., Hsieh, Y. H., Galbraith, J. S., Barnes, M., Waterfield, G., & Stanton, B. (2008). Focus-on-Teens, sexual risk-reduction intervention for high-school adolescents: Impact on knowledge, change of risk-behaviours, and prevalence of sexually transmitted diseases. *International Journal of STD & AIDS, 19*(10), 704-710. doi:http://dx.doi.org/10.1258/ijsa.2008.007291

Gielen, A. C. (2001). Promoting condom use with main partners: A behavioral intervention trial for women. *AIDS and Behavior, 5*(3), 193-204. doi:10.1023/A:1011391424690

Greenberg, J., Hennessy, M., MacGowan, R., Celentano, D., Gonzales, V., Van Devanter, N., & Lifshay, J. (2000). Modeling intervention efficacy for high-risk women: The WINGS Project. *Evaluation & the Health Professions, 23*, 123-148. doi:http://dx.doi.org/10.1177/016327870002300201

Grinstead Reznick, O., Comfort, M., McCartney, K., & Neilands, T. B. (2011). Effectiveness of an HIV prevention program for women visiting their incarcerated partners: the HOME Project. *AIDS Behav, 15*(2), 365-375. doi:10.1007/s10461-010-9770-4

Group, T. N. I. o. M. H. N. M. H. P. T. (1998). The NIMH Multisite HIV Prevention Trial: reducing HIV sexual risk behavior. The National Institute of Mental Health (NIMH) Multisite HIV Prevention Trial Group. *Science (New York, N.Y.), 280*(5371), 1889-1894.

Gruchow, H. W., & Brown, R. K. (2011). Evaluation of the Wise Guys Male Responsibility Curriculum: participant-control comparisons. *J Sch Health, 81*(3), 152-158. doi:10.1111/j.1746-1561.2010.00574.x

Harkabus, L. C., Harman, J. J., & Puntenney, J. M. (2013). Condom accessibility: The moderating effects of alcohol use and erotophobia in the information–motivation–behavioral skills model. *Health Promotion Practice, 14*(5), 751-758. doi:http://dx.doi.org/10.1177/1524839912465420

Harper, G. W., Bangi, A. K., Sanchez, B., Doll, M., & Pedraza, A. (2009). A Quasi-Experimental Evaluation of a Community Based HIV Prevention Intervention for Mexican American Female Adolescents: The Shero's Program. *AIDS Education and Prevention, 21*(5), 109-123.

Herbst, J. H., Painter, T. M., Tomlinson, H. L., & Alvarez, M. E. (2014). Evidence-based HIV/STD prevention intervention for black men who have sex with men. *Morbidity and mortality weekly report. Surveillance summaries (Washington, D.C. : 2002), 63 Suppl 1*, 21-27.

Hershberger, S. L., Wood, M. M., & Fisher, D. G. (2003). A Cognitive-Behavioral Intervention to Reduce HIV Risk Behaviors in Crack and Injection Drug Users. *AIDS and Behavior, 7*, 229-243. doi:http://dx.doi.org/10.1023/A:1025487501743

Hidalgo, M. A., Kuhns, L. M., Hotton, A. L., Johnson, A. K., Mustanski, B., & Garofalo, R. (2015). The MyPEEPS randomized controlled trial: A pilot of preliminary efficacy, feasibility, and acceptability of a group-level, HIV risk reduction intervention for young men who have sex with men. *Archives of Sexual Behavior, 44*(2), 475-485. doi:http://dx.doi.org/10.1007/s10508-014-0347-6

Hlaing, W. M., & Darrow, W. W. (2006). HIV risk reduction among young minority adults in Broward County. *J Health Care Poor Underserved, 17*, 159-173. doi:10.1353/hpu.2006.0087

Holtgrave, D. R., & Kelly, J. A. (1996). Preventing HIV/AIDS among high-risk urban women: the cost-effectiveness of a behavioral group intervention. *American Journal of Public Health, 86*(10), 1442-1445.

Holtgrave, D. R., & Kelly, J. A. (1997). Cost-effectiveness of an HIV/AIDS prevention intervention for gay men (Structured abstract). *AIDS and Behavior, 1*(3), 173-180.

Hosek, S. G., Lemos, D., Hotton, A. L., Fernandez, M. I., Telander, K., Footer, D., & Bell, M. (2015). An HIV intervention tailored for black young men who have sex with men in the House Ball Community. *AIDS Care, 27*(3), 355-362. doi:10.1080/09540121.2014.963016

Jemmott Iii, J. B., Jemmott, L. S., O'Leary, A., Icard, L. D., Rutledge, S. E., Stevens, R., . . . Stephens, A. J. (2014). On the Efficacy and Mediation of a One-on-One HIV Risk-Reduction Intervention for African American Men Who Have Sex with Men: A Randomized Controlled Trial. *AIDS Behav*. doi:10.1007/s10461-014-0961-2

Jemmott, J. B., 3rd, Jemmott, L. S., Braverman, P. K., & Fong, G. T. (2005). HIV/STD risk reduction interventions for African American and Latino adolescent girls at an adolescent medicine clinic: a randomized controlled trial. *Arch Pediatr Adolesc Med, 159*, 440-449. doi:10.1001/archpedi.159.5.440

Jemmott, J. B., 3rd, Jemmott, L. S., & Fong, G. T. (1998). Abstinence and safer sex HIV risk-reduction interventions for African American adolescents: a randomized controlled trial. *JAMA, 279*, 1529-1536.

Jemmott, J. B., III, Jemmott, L. S., Fong, G. T., & McCaffree, K. (1999). Reducing HIV risk-associated sexual behavior among African American adolescents: Testing the generality of intervention effects. *American Journal of Community Psychology, 27*, 161-187. doi:http://dx.doi.org/10.1007/BF02503158

Kalichman, S. C., Rompa, D., Cage, M., DiFonzo, K., Simpson, D., Austin, J., . . . Graham, J. (2001). Effectiveness of an intervention to reduce HIV transmission risks in HIV-positive people. *American Journal of Preventive Medicine, 21*, 84-92. doi:http://dx.doi.org/10.1016/S0749-3797(01)00324-5

Kalichman, S. C., Rompa, D., & Coley, B. (1996). Experimental component analysis of a behavioral HIV-AIDS prevention intervention for inner-city women. *Journal of Consulting and Clinical Psychology, 64*(4), 687-693.

Kalichman, S. C., Rompa, D., & Coley, B. (1997). Lack of positive outcomes from a cognitive-behavioral HIV and AIDS prevention intervention for inner-city men: lessons from a controlled pilot study. *AIDS Education and Prevention, 9*(4), 299-313.

Kalichman, S. C., Sikkema, K. J., Kelly, J. A., & Bulto, M. (1995). Use of a brief behavioral skills intervention to prevent HIV infection among chronic mentally ill adults. *Psychiatric services (Washington, D.C.), 46*(3), 275-280.

Kaufman, C. E., Whitesell, N. R., Keane, E. M., Desserich, J. A., Giago, C., Sam, A., & Mitchell, C. M. (2014). Effectiveness of Circle of Life, an HIV-preventive intervention for American Indian middle school youths: a group randomized trial in a Northern Plains tribe. *American Journal of Public Health, 104*(6), e106-112. doi:10.2105/AJPH.2013.301822

Kelly, J. A., McAuliffe, T. L., Sikkema, K. J., Murphy, D. A., Somlai, A. M., Mulry, G., . . . Fernandez, M. I. (1997). Reduction in risk behavior among adults with severe mental illness who learned to advocate for HIV prevention. *Psychiatric services (Washington, D.C.), 48*(10), 1283-1288.

Kelly, J. A., Murphy, D. A., Washington, C. D., Wilson, T. S., Koob, J. J., Davis, D. R., . . . Davantes, B. (1994). The effects of HIV/AIDS intervention groups for high-risk women in urban clinics. *Am J Public Health, 84*, 1918-1922.

Kelly, J. A., St Lawrence, J. S., Betts, R., Brasfield, T. L., & Hood, H. V. (1990). A skills-training group intervention model to assist persons in reducing risk behaviors for HIV infection. *AIDS Educ Prev, 2*, 24-35.

Kershaw, T. S., Magriples, U., Westdahl, C., Rising, S. S., & Ickovics, J. (2009). Pregnancy as a window of opportunity for HIV prevention: effects of an HIV intervention delivered within prenatal care. *Am J Public Health, 99*(11), 2079-2086. doi:10.2105/ajph.2008.154476

Kirby, D. B., Baumler, E., Coyle, K. K., Basen-Engquist, K., Parcel, G. S., Harrist, R., & Banspach, S. W. (2004). The "Safer Choices" Intervention: Its Impact on the Sexual Behaviors of Different Subgroups of High School Students. *Journal of Adolescent Health, 35*, 442-452. doi:http://dx.doi.org/10.1016/j.jadohealth.2004.02.006

Koblin, B. A., Bonner, S., Powell, B., Metralexis, P., Egan, J. E., Patterson, J., . . . Spikes, P. (2012). A randomized trial of a behavioral intervention for black MSM: The DiSH study. *AIDS, 26*(4), 483-488. doi:http://dx.doi.org/10.1097/QAD.0b013e32834f9833

Koniak-Griffin, D., & Stein, J. A. (2006). Predictors of sexual risk behaviors among adolescent mothers in a human immunodeficiency virus prevention program. *Journal of Adolescent Health, 38*. doi:10.1111/j.1559-1816.1998.tb01725.x http://dx.doi.org/10.1016/j.jadohealth.2004.12.008

Latham, T. P., Sales, J. M., Boyce, L. S., Renfro, T. L., Wingood, G. M., DiClemente, R. J., & Rose, E. (2010). Application of ADAPT-ITT: adapting an evidence-based HIV prevention intervention for

incarcerated African American adolescent females. *Health Promot Pract, 11*(3 Suppl), 53S-60S. doi:10.1177/1524839910361433

Linn, J. G., Neff, J. A., Theriot, R., Harris, J. L., Interrante, J., & Graham, M. E. (2003). Reaching impaired populations with HIV prevention programs: a clinical trial for homeless mentally ill African-American men. *Cellular and molecular biology (Noisy-le-Grand, France), 49*(7), 1167-1175.

MacGowan, R. J., Lifshay, J., Mizuno, Y., Johnson, W. D., McCormick, L., & Zack, B. (2014). Positive Transitions (POST): Evaluation of an HIV Prevention Intervention for HIV-Positive Persons Releasing from Correctional Facilities. *AIDS Behav*. doi:10.1007/s10461-014-0879-8

Mackesy-Amiti, M. E., Ouellet, L. J., Finnegan, L., Hagan, H., Golub, E., Latka, M., . . . Garfein, R. S. (2014). Transitions in latent classes of sexual risk behavior among young injection drug users following HIV prevention intervention. *AIDS and Behavior, 18*(3), 464-472. doi:10.1007/s10461-013-0601-2

Mahoney, M., Bien, M., & Comfort, M. (2013). Adaptation of an evidence-based HIV prevention intervention for women with incarcerated partners: expanding to community settings. *AIDS Educ Prev, 25*(1), 1-13. doi:10.1521/aeap.2013.25.1.1

Malotte, C. K., Jarvis, B., Fishbein, M., Kamb, M., Iatesta, M., Hoxworth, T., . . . Bolan, G. (2000). Stage of change versus an integrated psychosocial theory as a basis for developing effective behaviour change interventions. *AIDS Care, 12*, 357-364. doi:http://dx.doi.org/10.1080/09540120050043016

Malow, R. M., Devieux, J. G., Rosenberg, R., Dyer, J. G., & St Lawrence, J. S. (2006). Integrated HIV care: HIV risk outcomes of pregnant substance abusers. *Subst Use Misuse, 41*, 1745-1767. doi:10.1080/10826080601006458

Malow, R. M., McMahon, R. C., Devieux, J., Rosenberg, R., Frankel, A., Bryant, V., . . . Miguez, M. J. (2012). Cognitive behavioral HIV risk reduction in those receiving psychiatric treatment: a clinical trial. *AIDS Behav, 16*(5), 1192-1202. doi:10.1007/s10461-011-0104-y

Malow, R. M., West, J. A., Corrigan, S. A., Pena, J. M., & Cunningham, S. C. (1994). Outcome of psychoeducation for HIV risk reduction. *AIDS Educ Prev, 6*, 113-125.

Manlove, J., Ikramullah, E., & Terry-Humen, E. (2008). Condom use and consistency among male adolescents in the United States. *Journal of Adolescent Health, 43*(4), 325-333.

Mausbach, B. T., Semple, S. J., Strathdee, S. A., Zians, J., & Patterson, T. L. (2007). Efficacy of a behavioral intervention for increasing safer sex behaviors in HIV-positive MSM methamphetamine users: Results from the EDGE study. *Drug and Alcohol Dependence, 87*(2-3), 249-257. doi:http://dx.doi.org/10.1016/j.drugalcdep.2006.08.026

McCoy, C. B., De Gruttola, V., Metsch, L., & Comerford, M. (2011). A comparison of the efficacy of two interventions to reduce HIV risk behaviors among drug users. *AIDS Behav, 15*(8), 1707-1714. doi:10.1007/s10461-011-9975-1

Melendez, R. M., Zepeda, J., Samaniego, R., Chakravarty, D., & Alaniz, G. (2013). "La Familia" HIV prevention program: a focus on disclosure and family acceptance for Latino immigrant MSM to the USA. *Salud Publica Mex, 55 Suppl 4*, S491-497.

Miller, R. L. (1995). Assisting gay men to maintain safer sex: an evaluation of an AIDS service organization's safer sex maintenance program. *AIDS Educ Prev, 7*, 48-63.

Morrison-Beedy, D., Carey, M. P., Kowalski, J., & Tu, X. (2004). Group-based HIV risk reduction intervention for adolescent girls: evidence of feasibility and efficacy. *Res Nurs Health, 28*, 3-15. doi:10.1002/nur.20056

Mouttapa, M., Watson, D. W., McCuller, W. J., Reiber, C., Tsai, W., & Plug, M. (2010). HIV prevention among incarcerated male adolescents in an alternative school setting. *J Correct Health Care, 16*(1), 27-38. doi:10.1177/1078345809348202

Nelson, K., & Tom, N. (2011). Evaluation of a substance abuse, HIV and hepatitis prevention initiative for urban Native Americans: the Native Voices program. *J Psychoactive Drugs, 43*(4), 349-354. doi:10.1080/02791072.2011.629158

Nobles, W. W., Goddard, L. L., & Gilbert, D. J. (2009). Culturecology, Women, and African-Centered HIV Prevention. *Journal of Black Psychology, 35*(2), 228-246.

O'Hara, P., Messick, B. J., Fichtner, R. R., & Parris, D. (1996). A peer-led AIDS prevention program for students in an alternative school. *J Sch Health, 66*, 176-182.

O'Leary, A., Ambrose, T. K., Raffaelli, M., Maibach, E., Jemmott, L. S., Jemmott, J. B., 3rd, . . . Celentano, D. (1999). Effects of an HIV risk reduction project on sexual risk behavior of low-income STD patients. *AIDS Educ Prev, 10*, 483-492.

O'Donnell, L., Stueve, A., Joseph, H. A., & Flores, S. (2014). Adapting the VOICES HIV behavioral intervention for Latino men who have sex with men. *AIDS and Behavior, 18*(4), 767-775. doi:http://dx.doi.org/10.1007/s10461-013-0653-3

Otto-Salaj, L. L., Kelly, J. A., Stevenson, L. Y., Hoffmann, R., & Kalichman, S. C. (2001). Outcomes of a randomized small-group HIV prevention intervention trial for people with serious mental illness. *Community mental health journal, 37*(2), 123-144.

Patterson, T. L., Shaw, W. S., & Semple, S. J. (2003). Reducing the sexual risk behaviors of HIV+ individuals: Outcome of a randomized controlled trial. *Annals of Behavioral Medicine, 25*, 137-145. doi:http://dx.doi.org/10.1207/S15324796ABM2502_10

Peragallo, N., Deforge, B., O'Campo, P., Lee, S. M., Kim, Y. J., Cianelli, R., & Ferrer, L. (2005). A randomized clinical trial of an HIV-risk-reduction intervention among low-income Latina women. *Nurs Res, 54*, 108-118.

Pérez-Jiménez, D., Seal, D. W., & Ronis, D. L. (2014). A pilot intervention to promote safer sex in heterosexual Puerto Rican couples. *Couple and Family Psychology: Research and Practice, 3*(3), 193-206. doi:http://dx.doi.org/10.1037/cfp0000022

Pinkerton, S. D., Holtgrave, D. R., Johnson-Masotti, A. P., Turk, M. E., Hackl, K. L., & DiFranceisco, W. (2002). Cost-effectiveness of the NIMH multisite HIV prevention intervention (Structured abstract). *AIDS and Behavior, 6*(1), 83-96.

Pinkerton, S. D., Johnson-Masotti, A. P., Otto-Salaj, L. L., Stevenson, L. Y., & Hoffmann, R. G. (2001). Cost-effectiveness of an HIV prevention intervention for mentally ill adults. *Ment Health Serv Res, 3*, 45-55.

Ploem, C., & Byers, E. S. (1997). The effects of two AIDS risk-reduction interventions on heterosexual college women's AIDS-related knowledge, attitudes and condom use. *Journal of psychology & human sexuality, 9*(1), 1-24.

Popejoy, P. V. (1996). *Human immunodeficiency virus prevention education: An evaluation of "Slipping and Sliding". a small group intervention for gay men.* (AAM9624075).

Porter, R., Downey, R. A., McDougle, J., & Foley, L. (2013). Exploring the Impacts of a Community-Based HIV/AIDS Prevention Intervention in South Mississippi: The HOPE Project. *Journal of HIV/AIDS and Social Services, 12*(2), 224-235. doi:10.1080/15381501.2013.792206

Prado, G., Pantin, H., Huang, S., Cordova, D., Tapia, M. I., Velazquez, M. R., . . . Estrada, Y. (2012). Effects of a family intervention in reducing HIV risk behaviors among high-risk Hispanic adolescents: a randomized controlled trial. *Arch Pediatr Adolesc Med, 166*(2), 127-133. doi:10.1001/archpediatrics.2011.189

Raj, A., Amaro, H., Cranston, K., Martin, B., Cabral, H., Navarro, A., & Conron, K. (2002). Is a general women's health promotion program as effective as an HIV-intensive prevention program in reducing HIV risk among Hispanic women? *Public Health Rep, 116*, 599-607.

Reikowski, D. J. (1995). *A behavioral and cognitive intervention for AIDS prevention.* (AAM9429999).

Rhodes, S. D., McCoy, T. P., Vissman, A. T., DiClemente, R. J., Duck, S., Hergenrather, K. C., . . . Eng, E. (2011). A randomized controlled trial of a culturally congruent intervention to increase condom use and HIV testing among heterosexually active immigrant Latino men. *AIDS Behav, 15*(8), 1764-1775. doi:10.1007/s10461-011-9903-4

Ricketts, S. A., & Guernsey, B. P. (2006). School-based health centers and the decline in Black teen fertility during the 1990s in Denver, Colorado. *American Journal of Public Health, 96*(9), 1588-1592. doi:10.2105/AJPH.2004.059816

Roffman, R. A., Downey, L., Beadnell, B., Gordon, J. R., Craver, J. N., & Stephens, R. S. (1997). Cognitive-behavioral group counseling to prevent HIV transmission in gay and bisexual men: factors contributing to successful risk reduction. *Research on social work practice, 7*(2), 165-186.

Roffman, R. A., Stephens, R. S., Curtin, L., Gordon, J. R., Craver, J. N., Stern, M., . . . Downey, L. (1998). Relapse prevention as an interventive model for HIV risk reduction in gay and bisexual men. *AIDS Education and Prevention, 10*(1), 1-18.

Rotheram-Borus, M. J. (2006). HIV prevention with persons with mental health problems. *Psychology, health & medicine, 11*(2), 142-154. doi:10.1080/13548500500445094

Rotheram-Borus, M. J., Koopman, C., Haignere, C., & Davies, M. (1991). Reducing HIV sexual risk behaviors among runaway adolescents. *JAMA, 266*, 1237-1241.

Rotheram-Borus, M. J., Lee, M. B., Murphy, D. A., Futterman, D., Duan, N., Birnbaum, J. M., & Lightfoot, M. (2001). Efficacy of a preventive intervention for youths living with HIV. *American Journal of Public Health, 91*(3), 400-405.

Ruger, J. P., Abdallah, A. B., Ng, N. Y., Luekens, C., & Cottler, L. (2014). Cost-effectiveness of interventions to prevent HIV and STDs among women: a randomized controlled trial (Structured abstract). *AIDS and Behavior,* (2), epub.

Safren, S. A., O'Cleirigh, C., Skeer, M. R., Driskell, J., Goshe, B. M., Covahey, C., & Mayer, K. H. (2011). Demonstration and evaluation of a peer-delivered, individually-tailored, HIV prevention intervention for HIV-infected MSM in their primary care setting. *AIDS Behav, 15*(5), 949-958. doi:10.1007/s10461-010-9807-8

Safren, S. A., O'Cleirigh, C. M., Skeer, M., Elsesser, S. A., & Mayer, K. H. (2013). Project enhance: A randomized controlled trial of an individualized HIV prevention intervention for HIV-infected men who have sex with men conducted in a primary care setting. *Health Psychology, 32*(2), 171-179. doi:http://dx.doi.org/10.1037/a0028581

Sales, J. M., Lang, D. L., DiClemente, R. J., Latham, T. P., Wingood, G. M., Hardin, J. W., & Rose, E. S. (2012). The mediating role of partner communication frequency on condom use among African American adolescent females participating in an HIV prevention intervention. *Health psychology : official journal of the Division of Health Psychology, American Psychological Association, 31*(1), 63-69. doi:10.1037/a0025073

Sales, J. M., Lang, D. L., Hardin, J. W., Diclemente, R. J., & Wingood, G. M. (2010). Efficacy of an HIV prevention program among African American female adolescents reporting high depressive symptomatology. *Journal of women's health (2002), 19*(2), 219-227. doi:10.1089/jwh.2008.1326

Sánchez, J., Rosa, M., & Serna, C. A. (2013). Project Salud: Efficacy of a community-based HIV prevention intervention for Hispanic migrant workers in south Florida. *AIDS education and prevention : official publication of the International Society for AIDS Education, 25*(5), 363-375. doi:10.1521/aeap.2013.25.5.363

Sanchez, J., Silva-Suarez, G., Serna, C. A., & De La Rosa, M. (2012). The Latino Migrant Worker HIV Prevention Program Building a Community Partnership Through a Community Health Worker Training Program. *Family & Community Health, 35*(2), 139-146.

Schumann, A., Nyamathi, A., & Stein, J. A. (2007). HIV risk reduction in a nurse case-managed TB and HIV intervention among homeless adults. *J Health Psychol, 12*(5), 833-843. doi:10.1177/1359105307080618

Siegel, D., DiClemente, R. J., Durbin, M., Krasnovsky, F., & Saliba, P. (1995). Change in junior high school students' AIDS-related knowledge, misconceptions, attitudes, and HIV-preventive behaviors: Effects of a school-based intervention. *AIDS Education and Prevention, 7*(6), 534-543.

Sieving, R. E., Bernat, D. H., Resnick, M. D., Oliphant, J., Pettingell, S., Plowman, S., & Skay, C. (2012). A clinic-based youth development program to reduce sexual risk behaviors among adolescent girls: prime time pilot study. *Health Promot Pract, 13*(4), 462-471. doi:10.1177/1524839910386011

Smith, M. U., Dane, F. C., Archer, M. E., Devereaux, R. S., & Katner, H. P. (2000). Students together against negative decisions (STAND): evaluation of a school-based sexual risk reduction intervention in the rural south. *AIDS Educ Prev, 12*, 49-70.

Smith, M. U., & DiClemente, R. J. (2000). STAND: a peer educator training curriculum for sexual risk reduction in the rural South. Students Together Against Negative Decisions. *Prev Med, 30*, 441-449.

St Lawrence, J. S., Brasfield, T. L., Jefferson, K. W., Alleyne, E., O'Bannon, R. E., 3rd, & Shirley, A. (1995). Cognitive-behavioral intervention to reduce African American adolescents' risk for HIV infection. *J Consult Clin Psychol, 63*, 221-237.

St Lawrence, J. S., Crosby, R. A., Brasfield, T. L., & O'Bannon, R. E., 3rd. (2002). Reducing STD and HIV risk behavior of substance-dependent adolescents: a randomized controlled trial. *J Consult Clin Psychol, 70*, 1010-1021.

St Lawrence, J. S., Jefferson, K. W., Alleyne, E., & Brasfield, T. L. (1995). Comparison of education versus behavioral skills training interventions in lowering sexual HIV-risk behavior of substance-dependent adolescents. *J Consult Clin Psychol, 63*, 154-157.

St Lawrence, J. S., Jefferson, K. W., Banks, P. G., Cline, T. R., Alleyne, E., & Brasfield, T. L. (1994). Cognitive-behavioral group intervention to assist substance-dependent adolescents in lowering HIV infection risk. *AIDS Educ Prev, 6*, 425-435.

St Lawrence, J. S., Wilson, T. E., Eldridge, G. D., Brasfield, T. L., & O'Bannon, R. E., 3rd. (2002). Community-based interventions to reduce low income, African American women's risk of sexually transmitted diseases: a randomized controlled trial of three theoretical models. *Am J Community Psychol, 29*, 937-964.

Stanton, B., Harris, C., Cottrell, L., Li, X., Gibson, C., Guo, J., . . . Marshall, S. (2006). Trial of an urban adolescent sexual risk-reduction intervention for rural youth: A promising but imperfect fit. *Journal of Adolescent Health, 38*, e25-e36. doi:http://dx.doi.org/10.1016/j.jadohealth.2004.09.023

Susser, E., Valencia, E., Berkman, A., Sohler, N., Conover, S., Torres, J., . . . Miller, S. (1998). Human immunodeficiency virus sexual risk reduction in homeless men with mental illness. *Archives of General Psychiatry, 55*, 266-272. doi:http://dx.doi.org/10.1001/archpsyc.55.3.266

Sznitman, S., Vanable, P. A., Carey, M. P., Hennessy, M., Brown, L. K., Valois, R. F., . . . Romer, D. (2011). Using culturally sensitive media messages to reduce HIV-associated sexual behavior in high-risk African American adolescents: results from a randomized trial. *J Adolesc Health, 49*(3), 244-251. doi:10.1016/j.jadohealth.2010.12.007

Taylor, M., Montoya, J. A., Cantrell, R., Mitchell, S. J., Williams, M., Jordahl, L., . . . Roland, E. (2005). Interventions in the commercial sex industry during the rise in syphilis rates among men who have sex with men (MSM). *Sex Transm Dis, 32*, S53-59.

Tesar, C. M. (1996). *Peer education intervention and change in HIV-related knowledge, attitudes, intentions and behaviors in college students.* (AAM9633750).

Teti, M., Bowleg, L., Cole, R., Lloyd, L., Rubinstein, S., Spencer, S., . . . Gold, M. (2010). A mixed methods evaluation of the effect of the protect and respect intervention on the condom use and disclosure practices of women living with HIV/AIDS. *AIDS and Behavior, 14*(3), 567-579. doi:10.1007/s10461-009-9562-x

Tobin, K. E., Kuramoto, S. J., Davey-Rothwell, M. A., & Latkin, C. A. (2011). The STEP into Action study: a peer-based, personal risk network-focused HIV prevention intervention with injection drug users in Baltimore, Maryland. *Addiction, 106*(2), 366-375. doi:10.1111/j.1360-0443.2010.03146.x

Tolou-Shams, M., Houck, C., Conrad, S. M., Tarantino, N., Stein, L. A., & Brown, L. K. (2011). HIV prevention for juvenile drug court offenders: a randomized controlled trial focusing on affect management. *J Correct Health Care, 17*(3), 226-232. doi:10.1177/1078345811401357

Tross, S., Campbell, A. N. C., Cohen, L. R., Calsyn, D., Pavlicova, M., Miele, G. M., . . . Nunes, E. V. (2008). Effectiveness of HIV/STD sexual risk reduction groups for women in substance abuse treatment programs: Results of NIDA clinical trials network trial. *JAIDS Journal of Acquired Immune Deficiency Syndromes, 48*(5), 581-589. doi:http://dx.doi.org/10.1097/QAI.0b013e31817efb6e

Villarruel, A. M., Jemmott, J. B., & Jemmott, L. S. (2006). A randomized controlled trial testing an HIV prevention intervention for Latino youth. *Arch Pediatr Adolesc Med, 160*(8), 772-777. doi:10.1001/archpedi.160.8.772

Wilton, L., Herbst, J. H., Coury-Doniger, P., Painter, T. M., English, G., Alvarez, M. E., . . . Carey, J. W. (2009). Efficacy of an HIV/STI prevention intervention for black men who have sex with men:

findings from the Many Men, Many Voices (3MV) project. *AIDS Behav, 13*(3), 532-544. doi:10.1007/s10461-009-9529-y

Wingood, G. M., DiClemente, R. J., Villamizar, K., Er, D. L., DeVarona, M., Taveras, J., . . . Jean, R. (2011). Efficacy of a health educator-delivered HIV prevention intervention for Latina women: a randomized controlled trial. *American Journal of Public Health, 101*(12), 2245-2252. doi:10.2105/AJPH.2011.300340

Wu, E., El-Bassel, N., McVinney, L. D., Hess, L., Remien, R. H., Charania, M., & Mansergh, G. (2011). Feasibility and promise of a couple-based HIV/STI preventive intervention for methamphetamine-using, Black men who have sex with men. *AIDS and Behavior, 15*(8), 1745-1754. doi:http://dx.doi.org/10.1007/s10461-011-9997-8

**Excluded: Condom distribution not integral to the intervention (k=38)**

Anderson, E. S., Wagstaff, D. A., Heckman, T. G., Winett, R. A., Roffman, R. A., Solomon, L. J., . . . Sikkema, K. J. (2006). Information-Motivation-Behavioral Skills (IMB) Model: testing direct and mediated treatment effects on condom use among women in low-income housing. *Annals of Behavioral Medicine, 31*(1), 70-79. doi:10.1207/s15324796abm3101_11

Bearss, N., Santelli, J. S., & Papa, P. (1995). A pilot program of contraceptive continuation in six school-based clinics. *J Adolesc Health, 17*, 178-183. doi:10.1016/1054-139x(94)00189-l

Boise, R., Petersen, R., Curtis, K. M., Aalborg, A., Yoshida, C. K., Cabral, R., & Ballentine, J. M. (2003). Reproductive health counseling at pregnancy testing: a pilot study. *Contraception, 68*, 377-383.

Boyer, C. B., Barrett, D. C., Peterman, T. A., & Bolan, G. (1997). Sexually transmitted disease (STD) and HIV risk in heterosexual adults attending a public STD clinic: Evaluation of a randomized controlled behavioral risk-reduction intervention trial. *AIDS, 11*, 359-367. doi:http://dx.doi.org/10.1097/00002030-199703110-00014

Cohen, D., Scribner, R., Bedimo, R., & Farley, T. A. (1999). Cost as a barrier to condom use: the evidence for condom subsidies in the United States. *Am J Public Health, 89*, 567-568.

Collins, P. Y., Unger, H., Putnins, S., Crawford, N., Dutt, R., & Hoffer, M. (2011). Adding the female condom to HIV prevention interventions for women with severe mental illness: a pilot test. *Community mental health journal, 47*(2), 143-155. doi:10.1007/s10597-010-9302-8

Davey-Rothwell, M. A., Tobin, K., Yang, C., Sun, C. J., & Latkin, C. A. (2011). Results of a randomized controlled trial of a peer mentor HIV/STI prevention intervention for women over an 18 month follow-up. *AIDS and Behavior, 15*(8), 1654-1663. doi:10.1007/s10461-011-9943-9

Devanter, N., Gonzales, V., Merzel, C., Parikh, N. S., Celantano, D., & Greenberg, J. (2002). Effect of an STD/HIV behavioral intervention on women's use of the female condom. *American Journal of Public Health, 92*(1), 109-115.

Eaton, L. A., Kalichman, S. C., Kenny, D. A., & Harel, O. (2013). A reanalysis of a behavioral intervention to prevent incident HIV infections: including indirect effects in modeling outcomes of Project EXPLORE. *AIDS Care, 25*(7), 805-811. doi:10.1080/09540121.2012.748870

Emetu, R. E., Marshall, A., Sanders, S. A., Yarber, W. L., Milhausen, R. R., Crosby, R. A., & Graham, C. A. (2014). A novel, self-guided, home-based intervention to improve condom use among young

men who have sex with men. *J Am Coll Health, 62*(2), 118-124. doi:10.1080/07448481.2013.856914

Fang, X., Stanton, B., Li, X., Feigelman, S., & Baldwin, R. (1998). Similarities in sexual activity and condom use among friends within groups before and after a risk-reduction intervention. *Youth Soc, 29*, 431-450.

Fisher, H. H., Patel-Larson, A., Green, K., Shapatava, E., Uhl, G., Kalayil, E. J., . . . Chen, B. (2011). Evaluation of an HIV prevention intervention for African Americans and Hispanics: findings from the VOICES/VOCES Community-based Organization Behavioral Outcomes Project. *AIDS Behav, 15*(8), 1691-1706. doi:10.1007/s10461-011-9961-7

Fogel, C. I., Crandell, J. L., Neevel, A. M., Parker, S. D., Carry, M., White, B. L., . . . Gelaude, D. J. (2015). Efficacy of an adapted HIV and sexually transmitted infection prevention intervention for incarcerated women: a randomized controlled trial. *Am J Public Health, 105*(4), 802-809. doi:10.2105/ajph.2014.302105

Frye, V., Henny, K., Bonner, S., Williams, K., Bond, K. T., Hoover, D. R., . . . Koblin, B. A. (2013). "Straight Talk" for African-American heterosexual men: results of a single-arm behavioral intervention trial. *AIDS Care, 25*(5), 627-631. doi:10.1080/09540121.2012.722605

Gillmore, M. R., Morrison, D. M., Richey, C. A., Balassone, M. L., Gutierrez, L., & Farris, M. (1997). Effects of a skill-based intervention to encourage condom use among high risk heterosexually active adolescents. *AIDS Educ Prev, 9*, 22-43.

Harvey, S. M., Kraft, J. M., West, S. G., Taylor, A. B., Pappas-Deluca, K. A., & Beckman, L. J. (2009). Effects of a health behavior change model--based HIV/STI prevention intervention on condom use among heterosexual couples: a randomized trial. *Health Educ Behav, 36*(5), 878-894. doi:10.1177/1090198108322821

Hobfoll, S. E., Jackson, A. P., Lavin, J., Johnson, R. J., & Schröder, K. E. (2002). Effects and generalizability of communally oriented HIV-AIDS prevention versus general health promotion groups for single, inner-city women in urban clinics. *Journal of Consulting and Clinical Psychology, 70*(4), 950-960.

Kelly, J. A., Murphy, D. A., Sikkema, K. J., McAuliffe, T. L., Roffman, R. A., Solomon, L. J., . . . Kalichman, S. C. (1997). Randomised, controlled, community-level HIV-prevention intervention for sexual-risk behaviour among homosexual men in US cities. Community HIV Prevention Research Collaborative. *Lancet, 350*(9090), 1500-1505.

Kirby, D., Harvey, P. D., Claussenius, D., & Novar, M. (1989). A direct mailing to teenage males about condom use: its impact on knowledge, attitudes and sexual behavior. *Fam Plann Perspect, 21*, 12-18.

Koniak-Griffin, D., Lesser, J., Nyamathi, A., Uman, G., Stein, J. A., & Cumberland, W. G. (2003). Project CHARM: an HIV prevention program for adolescent mothers. *Family & Community Health, 26*(2), 94-107.

Latkin, C. A., Sherman, S., & Knowlton, A. (2003). HIV prevention among drug users: outcome of a network-oriented peer outreach intervention. *Health Psychology, 22*(4), 332-339.

Legardy, J. K., Macaluso, M., Artz, L., & Brill, I. (2005). Do participant characteristics influence the effectiveness of behavioral interventions? Promoting condom use to women. *Sex Transm Dis, 32*(11), 665-671.

Leukefeld, C., Roberto, H., Hiller, M., Webster, M., Logan, T. K., & Staton-Tindall, M. (2003). HIV prevention among high-risk and hard-to-reach rural residents. *J Psychoactive Drugs, 35*(4), 427-434.

Mallory, C., & Hesson-McInnis, M. (2013). Pilot test results of an HIV prevention intervention for high-risk women. *Western journal of nursing research, 35*(3), 313-329. doi:10.1177/0193945911416134

McCoy, H. V., McCoy, C. B., & Lai, S. (1998). Effectiveness of HIV interventions among women drug users. *Women & Health, 27*, 49-66. doi:http://dx.doi.org/10.1300/J013v27n01_04

Miller, R. L., Klotz, D., & Eckholdt, H. M. (1998). HIV prevention with male prostitutes and patrons of hustler bars: replication of an HIV preventive intervention. *American Journal of Community Psychology, 26*(1), 97-131.

Neumann, M. S., O'Donnell, L., Doval, A. S., Schillinger, J., Blank, S., Ortiz-Rios, E., . . . O'Donnell, C. R. (2011). Effectiveness of the VOICES/VOCES sexually transmitted disease/human immunodeficiency virus prevention intervention when administered by health department staff: does it work in the "real world"? *Sex Transm Dis, 38*(2), 133-139. doi:10.1097/OLQ.0b013e3181f0c051

Rickert, V. I., Gottlieb, A., & Jay, M. S. (1990). A comparison of three clinic-based AIDS education programs on female adolescents' knowledge, attitudes, and behavior. *J Adolesc Health Care, 11*, 298-303.

Rotheram-Borus, M. J., Song, J., Gwadz, M., Lee, M., Van Rossem, R., & Koopman, C. (2003). Reductions in HIV risk among runaway youth. *Prevention Science, 4*, 173-187. doi:http://dx.doi.org/10.1023/A:1024697706033

Sapiano, T. N., Moore, A., Kalayil, E. J., Zhang, X., Chen, B., Uhl, G., . . . Williams, W. (2013). Evaluation of an HIV prevention intervention designed for African American Women: results from the SISTA Community-Based Organization Behavioral Outcomes Project. *AIDS Behav, 17*(3), 1052-1067. doi:10.1007/s10461-012-0292-0

Shrier, L. A., Ancheta, R., Goodman, E., Chiou, V. M., Lyden, M. R., & Emans, S. J. (2001). Randomized controlled trial of a safer sex intervention for high-risk adolescent girls. *Arch Pediatr Adolesc Med, 155*, 73-79.

Sikkema, K. J., Anderson, E. S., Kelly, J. A., Winett, R. A., Gore-Felton, C., Roffman, R. A., . . . Brondino, M. J. (2005). Outcomes of a randomized, controlled community-level HIV prevention intervention for adolescents in low-income housing developments. *AIDS (London, England), 19*(14), 1509-1516.

Sikkema, K. J., Kelly, J. A., Winett, R. A., Solomon, L. J., Cargill, V. A., Roffman, R. A., . . . Mercer, M. B. (2000). Outcomes of a randomized community-level HIV prevention intervention for women living in 18 low-income housing developments. *American Journal of Public Health, 90*(1), 57-63.

Stanton, B. F., Li, X., Ricardo, I., Galbraith, J., Feigelman, S., & Kaljee, L. (1996). A randomized, controlled effectiveness trial of an AIDS prevention program for low-income African-American youths. *Arch Pediatr Adolesc Med, 150*, 363-372.

Tobin, K., Kuramoto, S. J., German, D., Fields, E., Spikes, P. S., Patterson, J., & Latkin, C. (2013). Unity in Diversity: Results of a Randomized Clinical Culturally Tailored Pilot HIV Prevention Intervention

Trial in Baltimore, Maryland, for African American Men Who Have Sex With Men. *Health Education & Behavior, 40*(3), 286-295.

Wechsberg, W. M., Lam, W. K., Zule, W. A., & Bobashev, G. (2004). Efficacy of a woman-focused intervention to reduce HIV risk and increase self-sufficiency among African American crack abusers. *American Journal of Public Health, 94*(7), 1165-1173.

Weeks, M. R., Coman, E., Hilario, H., Li, J., & Abbott, M. (2013). Initial and sustained female condom use among low-income urban U.S. women. *J Womens Health (Larchmt), 22*(1), 26-36. doi:10.1089/jwh.2011.3430

Wolitski, R. J. (2006). Relative Efficacy of a Multisession Sexual Risk–Reduction Intervention for Young Men Released From Prisons in 4 States. *American Journal of Public Health, 96*(10), 1854.

**Excluded: Multi-faceted CDI but outcome data not stratified (k=8)**

Anderko, L., & Uscian, M. (2002). Academic-community partnerships as a strategy for positive change in the sexual behavior of rural college-aged students. *Nurs Clin North Am, 37*, 341-349.

Cohen, D. A., Scribner, R., & Cory, D. (1992). Controlling a syphilis epidemic. *West J Med, 157*, 430-432.

Des Jarlais, D. C., Arasteh, K., McKnight, C., Feelemyer, J., Hagan, H., Cooper, H. L., & Perlman, D. C. (2014). Combined HIV prevention, the New York City condom distribution program, and the evolution of safer sex behavior among persons who inject drugs in New York City. *AIDS Behav, 18*(3), 443-451. doi:10.1007/s10461-013-0664-0

Jones, D. L., Weiss, S. M., Malow, R., Ishii, M., Devieux, J., Stanley, H., . . . Schneiderman, N. (2002). A brief sexual barrier intervention for women living with AIDS: acceptability, use, and ethnicity. *J Urban Health, 78*, 593-604. doi:10.1093/jurban/78.4.593

Marion, L. N., Finnegan, L., Campbell, R. T., & Szalacha, L. A. (2009). The Well Woman Program: a community-based randomized trial to prevent sexually transmitted infections in low-income African American women. *Res Nurs Health, 32*(3), 274-285. doi:10.1002/nur.20326

Martinez-Donate, A. P., Zellner, J. A., Fernandez-Cerdeno, A., Sanudo, F., Hovell, M. F., Sipan, C. L., . . . Ji, M. (2009). Hombres Sanos: exposure and response to a social marketing HIV prevention campaign targeting heterosexually identified Latino men who have sex with men and women. *AIDS Educ Prev, 21*(5 Suppl), 124-136. doi:10.1521/aeap.2009.21.5_supp.124

Martinez-Donate, A. P., Zellner, J. A., Sanudo, F., Fernandez-Cerdeno, A., Hovell, M. F., Sipan, C. L., . . . Carrillo, H. (2010). Hombres Sanos: evaluation of a social marketing campaign for heterosexually identified Latino men who have sex with men and women. *Am J Public Health, 100*(12), 2532-2540. doi:10.2105/ajph.2009.179648

Rhodes, S. D., Hergenrather, K. C., Bloom, F. R., Leichliter, J. S., & Montano, J. (2009). Outcomes from a community-based, participatory lay health adviser HIV/STD prevention intervention for recently arrived immigrant Latino men in rural North Carolina. *AIDS Educ Prev, 21*(5 Suppl), 103-108. doi:10.1521/aeap.2009.21.5_supp.103

**Excluded: Outcomes of interest not reported (k=12)**

Bedimo, A. L., Pinkerton, S. D., Cohen, D. A., Gray, B., & Farley, T. A. (2002). Condom distribution: a cost-utility analysis. *Int J STD AIDS, 13*, 384-392.

Cabral, R. J., Posner, S. F., Macaluso, M., Artz, L. M., Johnson, C., & Pulley, L. (2003). Do main partner conflict, power dynamics, and control over use of male condoms predict subsequent use of the female condom? *Women Health, 38*, 37-52. doi:10.1300/J013v38n01_03

Corby, N. H., & Wolitski, R. J. (1996). Condom use with main and other sex partners among high-risk women: Intervention outcomes and correlates of reduced risk. *Drugs & society (New York, N.Y.), 9*(1-2), 75-96. doi:10.1300/J023v09n01_05

Dahl, D. W., Gorn, G. J., & Weinberg, C. B. (1999). Encouraging use of coupons to stimulate condom purchase. *American Journal of Public Health, 89*(12), 1866-1869.

Exner, T. M., Tesoriero, J. M., Battles, H. B., Hoffman, S., Mantell, J. E., Correale, J., . . . Klein, S. J. (2012). A randomized controlled trial to evaluate a structural intervention to promote the female condom in New York state. *AIDS Behav, 16*(5), 1121-1132. doi:10.1007/s10461-012-0176-3

Feldman, C. (2014). *California Condom Access Project (CAP): Using the Internet to Improve Access to Condoms for Youth Across California.* Paper presented at the 2014 National STD Prevention Conference.

Jamner, M. S., Wolitski, R. J., & Corby, N. H. (1997). Impact of a longitudinal community HIV intervention targeting injecting drug users' stage of change for condom and bleach use. *American Journal of Health Promotion, 12*, 15-24. doi:http://dx.doi.org/10.4278/0890-1171-12.1.15

Khosropour, C., & Sullivan, P. S. (2013). Receipt and use of free condoms among US men who have sex with men. *Public Health Rep, 128*(5), 385-392.

Kissinger, P., Clark, R., Dumestre, J., & Bessinger, R. (1996). Incidence of three sexually transmitted diseases during a safer sex promotion program for HIV-infected women. *J Gen Intern Med, 11*, 750-752.

Penman-Aguilar, A., Macaluso, M., Peacock, N., Snead, M. C., & Posner, S. F. (2014). A novel approach to mixing qualitative and quantitative methods in HIV and STI prevention research. *AIDS Educ Prev, 26*(2), 95-108. doi:10.1521/aeap.2014.26.2.95

Van Devanter, N., Gonzales, V., Merzel, C., Parikh, N. S., Celantano, D., & Greenberg, J. (2002). Effects of an STD/HIV behavioral intervention on women's use of the female condom. *American Journal of Public Health, 92*, 109-115. doi:http://dx.doi.org/10.2105/AJPH.92.1.109

Witte, S. S., El-Bassel, N., Gilbert, L., Wu, E., Chang, M., & Hill, J. (2006). Promoting female condom use to heterosexual couples: findings from a randomized clinical trial. *Perspect Sex Reprod Health, 38*(3), 148-154. doi:10.1363/psrh.38.148.06

**Excluded: Insufficient quantitative information (k=2)**

Kennedy, M. G., Mizuno, Y., Seals, B. F., Myllyluoma, J., & Weeks-Norton, K. (2000). Increasing condom use among adolescents with coalition-based social marketing. *AIDS, 14*, 1809-1818.

Polen, M. R., & Freeborn, D. K. (1995). Outcome evaluation of project action. *Portland, OR: Oregon Health Division*.

### Excluded: Other reasons (e.g., reviews, non-US studies) (k=39)

Biasiolli, A., Vaughn, M., Brown, C., & Scott, A. (2012). Condom access in san antonio, texas: The Three A's and HIV/STI incidence. *International Quarterly of Community Health Education, 33*(4), 363-373. doi:10.2190/IQ.33.4.d

Brook, D. W., Brook, J. S., Whiteman, M., Win, P. T., Masci, J. R., Roberto, J., . . . Amundsen, F. (1998). Psychosocial risk and protective factors for condom use among female injection drug users. *Am J Addict, 7*, 115-127.

Brown, N. L., Pennylegion, M. T., & Hillard, P. (1997). A Process Evaluation of Condom Availability in the Seattle, Washington Public Schools. *Journal of school health, 67*(8), 336-340.

Burgos, J. L., Gaebler, J. A., Strathdee, S. A., Lozada, R., Staines, H., & Patterson, T. L. (2010). Cost-effectiveness of an intervention to reduce HIV/STI incidence and promote condom use among female sex workers in the Mexico-US border region (Provisional abstract). *Plos One, 5*(6), 1-10.

Control, C. f. D. (1991). Patterns of sexual behavior change among homosexual/bisexual men--selected US sites, 1987-1990. *MMWR Morb Mortal Wkly Rep, 40*(46), 792.

Control, C. f. D., & Prevention. (1993). Distribution of STD clinic patients along a stages-of-behavioral-change continuum--selected sites, 1993. *MMWR Morb Mortal Wkly Rep, 42*(45), 880.

De Rosa, C. J., Jeffries, R. A., Afifi, A. A., Cumberland, W. G., Chung, E. Q., Kerndt, P. R., . . . Dittus, P. J. (2012). Improving the implementation of a condom availability program in urban high schools. *J Adolesc Health, 51*(6), 572-579. doi:10.1016/j.jadohealth.2012.03.010

Edwards, K. E., Gibson, N., Martin, J., Mitchell, S., & Andersson, N. (2011). Impact of community-based interventions on condom use in the Tlicho region of Northwest Territories, Canada. *BMC Health Serv Res, 11 Suppl 2*, S9. doi:10.1186/1472-6963-11-s2-s9

English, A. (1993). Condom distribution in the schools. *Journal of Adolescent Health, 14*(7), 562-564. doi:10.1016/1054-139X(93)90141-B

Ethier, K. A., Dittus, P. J., DeRosa, C. J., Chung, E. Q., Martinez, E., & Kerndt, P. R. (2011). School-based health center access, reproductive health care, and contraceptive use among sexually experienced high school students. *Journal of Adolescent Health, 48*(6), 562-565.

Haley, T., Puskar, K., Terhorst, L., Terry, M. A., & Charron-Prochownik, D. (2013). Condom use among sexually active rural high school adolescents personal, environmental, and behavioral predictors. *J Sch Nurs, 29*(3), 212-224. doi:10.1177/1059840512461282

Holtgrave, D. R., Maulsby, C., Kharfen, M., Jia, Y., Wu, C., Opoku, J., . . . Pappas, G. (2012). Cost-utility analysis of a female condom promotion program in Washington, DC. *AIDS Behav, 16*(5), 1115-1120. doi:10.1007/s10461-012-0174-5

Jones, K. T., Gray, P., Whiteside, Y. O., Wang, T., Bost, D., Dunbar, E., . . . Johnson, W. D. (2008). Evaluation of an HIV prevention intervention adapted for Black men who have sex with men. *American Journal of Public Health, 98*(6), 1043-1050. doi:http://dx.doi.org/10.2105/AJPH.2007.120337

Kerr, D. (1994). Condom availability in New York City schools. *J Sch Health, 61*, 279-280.

Kerr, D. L. (1991). AIDS update. Condom availability in New York City schools. *J Sch Health, 61*, 279-280.

Khan, S. I. (2013). Structural Interventions to Work with Sexuality, Sexual Health Including HIV Preventions for Key-Populations at Risk: A Paradigm Shift Is Required for Sustainability. *Journal of Sexual Medicine, 10*, 280-280.

Kirby, D. (2002). The impact of schools and school programs upon adolescent sexual behavior. *Journal of Sex Research, 39*(1), 27-33.

Kirby, D. (2005). An HIV-prevention intervention for African American adolescent girls significantly increased condom use. *Evidence-based Obstetrics and Gynecology, 7*(2), 74-75.

Kirby, D., Waszak, C., & Ziegler, J. (1991). Six school-based clinics: Their reproductive health services and impact on sexual behavior. *Fam Plann Perspect, 23*(1), 6-16.

Kirby, D. B., & Brown, N. L. (1996). Condom Availability Programs in U.S. Schools. *Fam Plann Perspect, 28*(5), 196-202.

Ko, N. Y., Lee, H. C., Hung, C. C., Chang, J. L., Lee, N. Y., Chang, C. M., . . . Ko, W. C. (2009). Effects of structural intervention on increasing condom availability and reducing risky sexual behaviours in gay bathhouse attendees. *AIDS Care, 21*(12), 1499-1507. doi:10.1080/09540120902923022

Koniak-Griffin, D., Lesser, J., Henneman, T., Rong, H., Xin, H., Tello, J., . . . Cumberland, W. G. (2008). HIV prevention for Latino adolescent mothers and their partners. *West J Nurs Res, 30*(6), 724-742. doi:10.1177/0193945907310490

Leibowitz, A. A., Harawa, N., Sylla, M., Hallstrom, C. C., & Kerndt, P. R. (2013). Condom distribution in jail to prevent HIV infection. *AIDS Behav, 17*(8), 2695-2702. doi:10.1007/s10461-012-0190-5

Parkes, A., Henderson, M., & Wight, D. (2005). Do sexual health services encourage teenagers to use condoms? A longitudinal study. *J Fam Plann Reprod Health Care, 31*, 271-280. doi:10.1783/1471189054

Raab, M. (1998). Condom availability in high school does not increase teenage sexual activity but does increase condom use. *Perspect Sex Reprod Health, 30*(1), 48.

Renaud, T. C., Bocour, A., Irvine, M. K., Bernstein, K. T., Begier, E. M., Sepkowitz, K. A., . . . Weglein, D. (2009). The free condom initiative: promoting condom availability and use in New York City. *Public Health Rep, 124*(4), 481-489.

Rietmeijer, C. A. (2013). Structural interventions for sexually transmitted infection prevention and sexual health. *Sex Transm Dis, 40*(8), 655-656. doi:10.1097/olq.0000000000000018

Rietmeijer, C. A., Kane, M. S., Simons, P. Z., Corby, N. H., Wolitski, R. J., Higgins, D. L., . . . Cohn, D. L. (1996). Increasing the use of bleach and condoms among injecting drug users in Denver: Outcomes of a targeted, community-level HIV prevention program. *AIDS (London, England), 10*(3), 291-298.

Sales, J. M., Brown, J. L., Diclemente, R. J., & Rose, E. (2012). Exploring Factors Associated with Nonchange in Condom Use Behavior following Participation in an STI/HIV Prevention Intervention for African-American Adolescent Females. *AIDS Res Treat, 2012*, 231417. doi:10.1155/2012/231417

Sayegh, A., Rose, S., & Schapiro, N. A. (2012). Condom Availability in Middle Schools: Evidence and Recommendations. *Journal of Pediatric Health Care, 26*(6), 471-475. doi:10.1016/j.pedhc.2012.07.018

Schuster, M. A., Bell, R. M., Berry, S. H., & Kanouse, D. E. (1997). Students' acquisition and use of school condoms in a high school condom availability program. *Pediatrics, 100*(4), 689-694. doi:10.1542/peds.100.4.689

Soleimanpour, S., Geierstanger, S. P., Kaller, S., McCarter, V., & Brindis, C. D. (2010). The role of school health centers in health care access and client outcomes. *Am J Public Health, 100*(9), 1597-1603.

Song, Y. S., Calsyn, D. A., Doyle, S. R., Dierst-Davies, R., Chen, T., & Sorensen, J. L. (2009). Predictors of condom use among men enrolled in drug treatment programs. *AIDS Educ Prev, 21*(5), 460-473. doi:10.1521/aeap.2009.21.5.460

Taylor, P. (1991, March 10, 1991). High School Clinic: Case Study in Sexual Risk; In Rural Florida Town, Impact of Offering Free Condoms to Teenagers Remains Unclear. *The Washington Post*.

Walls, C. T., Lauby, J., Lavelle, K., Derby, T., & Bond, L. (1998). Exposure to a community-level HIV prevention intervention: who gets the message. *J Community Health, 23*, 281-299.

Warner, L., & Steiner, M. J. (2002). Condom access does not ensure condom use: You've got to be putting me on [4]. *Sex Transm Infect, 78*(3), 225. doi:10.1136/sti.78.3.225

Wechsberg, W. M., Dennis, M. L., & Stevens, S. J. (1998). Cluster analysis of HIV intervention outcomes among substance-abusing women. *Am J Drug Alcohol Abuse, 24*, 239-257.

Wechsberg, W. M., Novak, S. P., Zule, W. A., Browne, F. A., Kral, A. H., Ellerson, R. M., & Kline, T. (2010). Sustainability of intervention effects of an evidence-based HIV prevention intervention for African American women who smoke crack cocaine. *Drug and Alcohol Dependence, 109*(1-3), 205-212. doi:10.1016/j.drugalcdep.2010.01.014

Wolk, L. I., & Rosenbaum, R. (1995). The benefits of school-based condom availability: cross-sectional analysis of a comprehensive high school-based program. *J Adolesc Health, 17*, 184-188. doi:10.1016/1054-139x(95)00031-m
