# Supplementary material for: Effectiveness of community-based condom distribution interventions to prevent HIV in the United States: A systematic review and meta-analysis
Source: PLoS One. 2017 Aug 3;12(8):e0180718. doi: 10.1371/journal.pone.0180718 (PMC5542551; doi:10.1371/journal.pone.0180718)
Supplement: S5 File — (PDF) [file pone.0180718.s005.pdf]

S5: Limited CDI studies

Systematic Review of Community-based Condom Distribution Interventions in the US: UCSF CAPE Project

Table of Contents

TABLE 1. CHARACTERISTICS OF "LIMITED" COMMUNITY-BASED CONDOM DISTRIBUTION PROGRAMS IN THE UNITED STATES.. 1

FIG 1. POOLED EFFECT MEASURES AND RISK OF BIAS FOR THE EFFECT OF "LIMITED" COMMUNITY-BASED CONDOM DISTRIBUTION INTERVENTIONS (COMPARED TO NO CONDOM DISTRIBUTION) ON SEXUAL RISK BEHAVIORS IN THE U.S. 3

TABLE 2: GRADE EVIDENCE PROFILES OF "LIMITED" COMMUNITY-BASED CONDOM DISTRIBUTION INTERVENTIONS (COMPARED TO NO CONDOM DISTRIBUTION) ON SEXUAL RISK BEHAVIORS IN THE U.S. 6

REFERENCES 8

Table 1. Characteristics of “Limited” community-based condom distribution programs in the United States.

Author & Year	Study location (Setting)	Data Collection Year	Target population (inclusion/exclusion criteria)	Demographic information	Co-interventions (Provider / delivery modality)[a]	Study design[b] (Number of participants)	Reported outcomes	Follow-up period
**Artz 2000 [1]**	Birmingham and Huntsville, AL (Urban)	Jul 1995 - Sept 1997	High risk women (age 18-35 yrs., approached in a public health STD clinic)	Age: median 23 yrs.<br>Sex (% F): 100%<br>Race/Ethnicity: 84% Afr. Amer.	Formal CUT + Behavioral & skill building (Professional staff + Peers + Media)	Experimental[c] Single-Arm Pre-Post Individual (1,159)	Condom use for 100% of vaginal intercourse acts	~6 mo. (analysis based on 6th visit mean/ median 25/40 days apart)
**Choi 2008 [2]**	Concord, Mountain View, Santa Cruz, and San Francisco, CA (Urban)	2003-2005	Women (18-39 yrs., attending a family planning clinic, 2 or more male sexual partners in the prior year, HIV negative, not a commercial sex worker)	Age: mean 22 yrs.<br>Sex (% F): 100%<br>Race/Ethnicity: 11% Afr. Amer.; 6% Asian; 17% Latina; 64% White	Gen. health promotion arm: Gen. HIV & sex health edu + Formal CUT (Professional staff)<br><br>Skills training arm: Gen. HIV & sex health edu + Formal CUT + Behavioral & skill building (Professional staff)	Experimental Pre-Post Single-Arm Individual (196 Gen. health promotion arm, and 213 Skills training arm)	Percentage of vaginal or anal intercourse protected by any condom	~7 mo.
**Collins 1999 [3]**	Birmingham, AL (Urban)	1996-1998	Adult drug users (approached in areas near drug marketplaces, not in drug abuse treatment)	Age: range of means 32.8-35.9 yrs. across 3 waves<br>Sex (% F): range 34.5%-48.9% across 3 wavesRace/Ethnicity: "Cauc. (less than 1% of respondents) were excluded from further analysis because the sample was too small. >50% of respondents were Afr. Amer. males"	Behavioral & skill building + Street outreach (Peers + Media)	Experimental Double-Arm Pre-Post Cross-Sectional[d] (2,059 across 3 cross-sect. waves)	% currently using condoms every time; Number of sex partners	12 mo.; 22 mo.
**Fishbein 1999 [4]**	Dallas, TX; Denver, CO; Long Beach, CA; New York, NY; Seattle, WA (Urban)	1991-1994	High risk adults[e] (sexually active in the past 30 days or shared needles for drug injection in past 60 days)	Age: 35.4% 11-29 yrs.; 39.7% 30-39; 24.9% 40-87<br>Sex (% F): 54.5%<br>Race/Ethnicity: 54.3% Afr. Amer.; 22.0% White; 18.6% Hisp; 5% other	Behavioral & skill building + Street outreach + Bleach kit distribution (Peers + Media)	Experimental Dbl-Arm Pre-Post Cross-Sectional (15,205 across 10 cross-sect. waves)	% currently using condoms every time	40 mo.
**Kegeles 1996 [5]**	Eugene, OR and Santa Barbara,	NR	Young MSM (age 18-29 yrs.)	Age: mean 23.4 yrs.<br>Sex (% F): 0%	Gen. HIV & sex health edu + Formal	Experimental Non-RCT Double-Arm	Any unprotected	~1 year

	CA (Urban)			Race/Ethnicity: 81% White; 6% Latino; 4% Afr. Amer.; 7% Asian/Pacific Islander; 2% Other	CUT + Behavioral & skill building (Peers + Media)	Cluster (300)	anal intercourse	
**Watters 1990 [6]**	San Francisco, CA (Urban)	1986 - 1987	PWID (no other criteria specified)	Age: (1986; 1987 cross-section) 64.8%; 53.5% under age 36<br>Sex (% F): "1/3"<br>Race/Ethnicity: (1986, 1987 cross-section) 50.7%, 39.6% White; 27.4%, 40.8% Black; 10.5%, 11.7% Hisp; 11.4%, 7.9% other	Street outreach + Distribution of bleach kits and injective risk reduction messages (Professional staff)	Experimental Single-Arm Pre-Post Cross-Sectional (1,061)	% used condoms at least half the time	9 mo.
**Wendell 2003 [7]**	Statewide/LA (Urban/rural)	Apr-Sept 1998 - Apr-Sept 1999	Gen. pop. (age 12-65, approached in a high risk neighborhood, 1+ partner in the last 12 mo.)	Age: 1.6% 12-14 yrs.; 26.1% 15-19; 23.8% 20-24; 27.3% 25-34; 21.2% 35+<br>Sex (% F): 45.9%<br>Race/Ethnicity: 83.7% Afr. Amer.; 6.6% White; 3.4% Other	Street outreach + Bleach kit and needle coupon distribution (Peers)	Double-Arm Retrospective Cohort Cluster (6,547)	Used condom at last sex; Two or more sexual partners	NR

CD, Condom distribution; CUT, Condom use training; MSM, Men who have sex with men; N/A, Not applicable; NR, Not reported; PWID, People who inject drugs; STD, Sexually transmitted disease.

Footnotes:

[a] Intervention category and co-interventions listed are those that comprise the unique elements tested in the study (i.e., common elements provided to both the intervention and control group are not be listed).

[b] Study design reflects the way reported data were analyzed in this review in order to extract an effect of condom distribution. It does not always match the design of the study as originally implemented.

[c] Studies are considered experimental if investigators control the intervention allocation.

[d] Outcome "risk of not always using condoms" analyzed using Experimental Double-arm Post-Test Only Cross-Sectional design.

[e] Active injection drug users, female sex partners of male injection drug users, female commercial sex workers and other women who trade sex for money or drugs, youth in high risk situations, non-gay-identified MSM, residents of census tracts where rates of STIs are high.

Fig 1. Pooled effect measures and risk of bias for the effect of “Limited” community-based condom distribution interventions (compared to no condom distribution) on sexual risk behaviors in the U.S.

Outcome and study	Risk Ratio (95% CI)	Weight (%)	Hetero geneity (I^2)	Forest plot<br>Random Effects Model<br><-Favors Intervention Favors comparator->	Risk of bias						
					A	B	C	D	E	F	G
**Condomless sex likelihood, all studies**				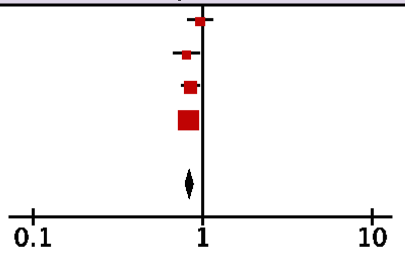							
Choi 2008	0.97 (0.82 to 1.15)[a,b]	14.06			?	+	?	+	-	+	+
Choi 2008	0.8 (0.67 to 0.96)[b,c]	12.63			?	+	?	+	-	+	+
Watters 1990	0.85 (0.75 to 0.96)	24.88			?	?	-	+	-	+	+
Wendell 2003	0.83 (0.76 to 0.91)[d]	48.43			?	?	-	?	-	-	+
**TOTAL**	0.85 (0.8 to 0.91)	100	3.2								
**Condomless sex likelihood, females**				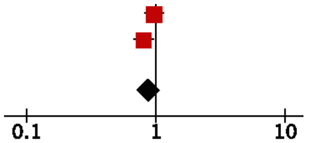							
Choi 2008	0.97 (0.82 to 1.15)[a,b]	51.14			?	+	?	+	-	+	+
Choi 2008	0.8 (0.67 to 0.96)[b,c]	48.86			?	+	?	+	-	+	+
**TOTAL**	0.88 (0.73 to 1.07)	100	58.12								
**Condomless sex likelihood, drug users**											
Watters 1990	0.85 (0.75 to 0.96)	100			?	?	-	+	-	+	+
**Condomless sex likelihood, follow-up ≤1 year**				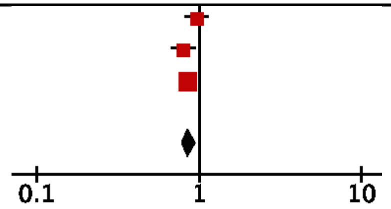							
Choi 2008	0.97 (0.82 to 1.15)[a,b]	28.88			?	+	?	+	-	+	+
Choi 2008	0.8 (0.67 to 0.96)[b,c]	26.46			?	+	?	+	-	+	+
Watters 1990	0.85 (0.75 to 0.96)	44.66			?	?	-	+	-	+	+
**TOTAL**	0.87 (0.79 to 0.96)	100	23.03								
**Not always using condoms, all studies**				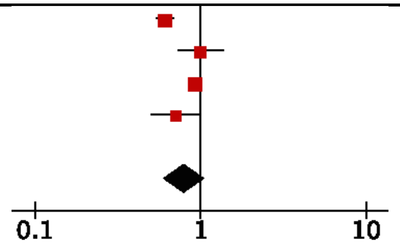							
Artz 2000	0.61 (0.54 to 0.69)[e]	28.39			-	?	-	+	-	+	+
Collins 1999	1 (0.73 to 1.37)[f,g]	21.73			?	?	-	?	-	?	?
Fishbein 1999	0.93 (0.87 to 0.99)[h]	29.5			?	?	+	?	-	+	+
Kegeles 1996	0.71 (0.5 to 1.01)[i]	20.38			?	?	+	+	-	+	+
**TOTAL**	0.79 (0.6 to 1.05)	100	91.99								
**Not always using condoms, males**											
Kegeles 1996	0.71 (0.5 to 1.01)[i]	100			?	?	+	+	-	+	+

					A	B	C	D	E	F	G
**Not always using condoms, females**											
Artz 2000	0.61 (0.54 to 0.69)[e]	100			-	?	-	+	-	+	+
**Not always using condoms, drug users**											
Collins 1999	1 (0.73 to 1.37)[f,g]	100			?	?	-	?	-	?	?
**Not always using condoms, follow-up ≤1 year**				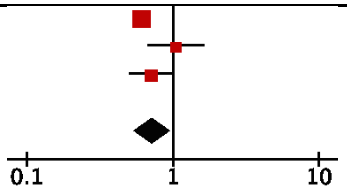							
Artz 2000	0.61 (0.54 to 0.69)[e]	47.78			-	?	-	+	-	+	+
Collins 1999	1.05 (0.67 to 1.65)[g]	22.89			?	?	-	?	-	?	?
Kegeles 1996	0.71 (0.50 to 1.01)[i]	29.33			?	?	+	+	-	+	+
**TOTAL**	0.72 (0.54 to 0.96)	100	64.27								
**Not always using condoms, follow-up >1 year**				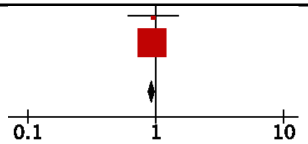							
Collins 1999	0.95 (0.6 to 1.5)[g]	2.06			?	?	-	?	-	?	?
Fishbein 1999	0.93 (0.87 to 0.99)[h]	97.94			?	?	+	?	-	+	+
**TOTAL**	0.93 (0.87 to 0.99)	100	0								
**Multiple sexual partners, all studies**				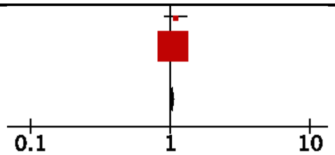							
Collins 1999	1.09 (0.90 to 1.32)[f,j]	4.02			?	?	-	?	-	+	?
Wendell 2003	1.04 (1 to 1.08)	95.98			?	?	-	?	-	-	?
**TOTAL**	1.04 (1 to 1.08)	100	0								
**Multiple sexual partners, follow-up ≤1 year, drug users**											
Collins 1999	1 (0.86 to 1.16)[j]	100			?	?	-	?	-	+	?
**Multiple sexual partners, follow-up >1 year, drug users**											
Collins 1999	1.22 (0.98 to 1.52)[j]	100			?	?	-	?	-	+	?

Risk of bias legend:
(A) Incomplete outcome data (attrition bias)
(B) Selective reporting (reporting bias)
(C) Other bias
(D) Failure to develop and apply appropriate eligibility criteria
(E) Flawed measurement of exposure and/or outcome
(F) Failure to control for confounders
(G) Too-short or incomplete length of follow-up
- = high risk of bias
+ = low risk of bias
? = unclear risk of bias

Footnotes:
[a] General Health Promotion group only, treated as a pre-post design.
[b] Assumed group retention rate equal to reported overall retention (85%), proportion sexually active equal to baseline (97%), and no item non-

response. Used proportions reported in publication to back calculate 2x2 table to calculate RR and CI.
[c] Skills Training group only, treated as a pre-post design.
[d] Since crude odds ratio is very similar to reported adjusted odds ratio, used proportions in Table 3 to calculate crude RR. Used reported CI to calculate Z score, and used the Z score to calculated CI for the RR.
[e] Paired results not available, effect calculated as a two sample test (a more conservative test).
[f] Combined effect across 12-month and 22-month cross-sectional samples.
[g] Used inverse of bivariate odds ratio and CI (as reported in publication) for the effect of residence neighborhood on “action or higher” stage of change (always using a condom for any length of time). Since odds ratio is close to 1, we treated the OR as an RR.
[h] Combined effect across main and non-main partners. Calculated RR using actual probability at follow-up in intervention group and using a counterfactual denominator equal to the baseline rate in the intervention group plus the change from baseline to follow-up in the control group. Calculated p-value based on CI difference in differences estimate, and used that to calculate CI for RR.
[i] Assumed counterfactual rate equal to baseline risk in intervention group plus the change from baseline to follow-up in the control group. The reported p-value (<0.03) does not correspond with z-value (1.75). To calculate CI for RR, we used a p-value equal to the mean of the given p-value and the appropriate 2-sided p-value for the given z-score (i.e., used p=.08).
[j] Effect reported as “Number of sex partners.” Converted to dichotomous variable (2 or more partners) by assuming a Poisson distribution and no item non-response (CI calculated using 10,000 iterations of simulated samples).

Table 2: GRADE evidence profiles of "Limited" community-based condom distribution interventions (compared to no condom distribution) on sexual risk behaviors in the U.S.

Question: Should Limited condom distribution with co-interventions (compared to no condom distribution) be used for preventing HIV infection in the US?
Date: December 10, 2015
Settings: United States
Bibliography: Artz 2000, Choi 2008[a], Collins 1999[b], Fishbein 1999[b], Kegeles 1996, Watters 1990[b], Wendell 2003[b]

Outcome: Change in HIV incidence (reported surrogate outcomes)	Design	Number of studies (number of participants)	Relative effect (95% CI)	Quality of the evidence (GRADE)	Comments
Condomless sex likelihood, all studies	Observational	4 (7,956)	**RR 0.85** (0.80 to 0.91)	⊕⊖⊖⊖ **VERY LOW**	Graded down for very serious risk of bias and very serious indirectness.[c,d,e]
Condomless sex likelihood, females	Observational	2 (348)	**RR 0.88** (0.73 to 1.07)	⊕⊖⊖⊖ **VERY LOW**	Graded down for very serious risk of bias and serious indirectness.[c,d]
Condomless sex likelihood, drug users	Observational	1 (1,061)	**RR 0.85** (0.75 to 0.96)	⊕⊖⊖⊖ **VERY LOW**	Graded down for very serious risk of bias and very serious indirectness[c,d,e]
Condomless sex likelihood, follow-up ≤1 year [7-9 months]	Observational	3 (1,409)	**RR 0.87** (0.79 to 0.96)	⊕⊖⊖⊖ **VERY LOW**	Graded down for very serious risk of bias and very serious indirectness.[c,d,e]
Not always using condoms, all studies	Observational	4 (17,859)	**RR 0.79** (0.60 to 1.05)	⊕⊖⊖⊖ **VERY LOW**	Graded down for very serious risk of bias and serious indirectness.[c,d]
Not always using condoms, males	Observational	1 (188)	**RR 0.71** (0.50 to 1.01)	⊕⊖⊖⊖ **VERY LOW**	Graded down for very serious risk of bias and serious indirectness.[c,d]
Not always using condoms, females	Observational	1 (407)	**RR 0.61** (0.54 to 0.69)	⊕⊖⊖⊖ **VERY LOW**	Graded down for very serious risk of bias and serious indirectness.[c,d]
Not always using condoms, drug users	Observational	1 (2,059)	**RR 1.00** (0.73 to 1.37)	⊕⊖⊖⊖ **VERY LOW**	Graded down for very serious risk of bias and very serious indirectness.[c,d]
Not always using condoms, follow-up ≤1 year [6-12 months]	Observational	3 (1,889)	**RR 0.72** (0.54 to 0.96)	⊕⊖⊖⊖ **VERY LOW**	Graded down for very serious risk of bias and serious indirectness.[c,d]

Not always using condoms, follow-up >1 year [22-40 months], males and females	Observational	2 (16,492)	**RR 0.93** (0.87 to 0.99)	⊕⊖⊖⊖ **VERY LOW**	Graded down for very serious risk of bias and very serious indirectness.[c,d,e]
Multiple sexual partners, all studies	Observational	2 (8,606)	**RR 1.04** (1.00 to 1.08)	⊕⊖⊖⊖ **VERY LOW**	Graded down for very serious risk of bias and very serious indirectness.[c,d,e]
Multiple sexual partners, follow-up ≤1 year [12 months], drug users	Observational	1 (1,249)	**RR 1.00** (0.86 to 1.16)	⊕⊖⊖⊖ **VERY LOW**	Graded down for very serious risk of bias and very serious indirectness.[c,d,e]
Multiple sexual partners, follow-up >1 year [22 months], drug users	Observational	1 (1,287)	**RR 1.22** (0.98 to 1.52)	⊕⊖⊖⊖ **VERY LOW**	Graded down for very serious risk of bias and very serious indirectness.[c,d,e]

Abbreviations:
RR, risk ratio; CI, confidence interval

GRADE Working Group grades of evidence:

⊕⊕⊕⊕	**HIGH**	We are very confident that the true effect lies close to that of the estimate of the effect. Further research is unlikely to substantially change the estimate.
⊕⊕⊕⊖	**MODERATE**	We are moderately confident in the effect estimate: The true effect is likely to be close to the estimate of the effect, but there is a possibility that it is substantially different.
⊕⊕⊖⊖	**LOW**	Our confidence in the effect estimate is limited: The true effect may be substantially different from the estimate of the effect.
⊕⊖⊖⊖	**VERY LOW**	We have very little confidence in the effect estimate: The true effect is likely to be substantially different from the estimate of effect.

Footnotes:
[a] Both randomized interventions in this trial included condom distribution, so trial was treated as two-arm pre-post study, with each randomized arm contributing an independent pre-post effect size. The exact number of participants included in analyses was not reported, so we used an estimated number based on and overall retention rate of 85% in each group.
[b] Our sample size includes all study participants because item-level response rate and/or actual number analyzed was not reported.
[c] Flawed measurement of exposure and outcome. All studies single-arm pre/post studies. Graded down by 2 for high risk of bias.
[d] Studies did not assess incident HIV. Graded down by 1 for indirectness.
[e] Participants in Collins and Watters studies were all drug users. Graded down by 1 for indirectness.

References

1. Artz, L., et al., *Effectiveness of an intervention promoting the female condom to patients at sexually transmitted disease clinics.* American Journal of Public Health, 2000. **90**(2): p. 237.
2. Choi, K.H., et al., *The efficacy of female condom skills training in HIV risk reduction among women: a randomized controlled trial.* Am J Public Health, 2008. **98**(10): p. 1841-8.
3. Collins, C., et al., *Evaluation of the exposure effects of a theory-based street outreach HIV intervention on African-American drug users.* Eval Program Plann, 1999. **22**: p. 279-93.
4. Fishbein, M., et al., *Community-level HIV intervention in 5 cities: final outcome data from the CDC AIDS Community Demonstration Projects.* American Journal of Public Health, 1999. **89**(3): p. 336.
5. Kegeles, S.M., R.B. Hays, and T.J. Coates, *The Mpowerment Project: a community-level HIV prevention intervention for young gay men.* Amercian Journal of Public Health, 1996. **86**(8): p. 1129-36.
6. Watters, J.K., et al., *AIDS prevention for intravenous drug users in the community: street-based education and risk behavior.* Am J Community Psychol, 1990. **18**: p. 587-96.
7. Wendell, D.A., et al., *Street outreach for HIV prevention: effectiveness of a state-wide programme.* Int J STD AIDS, 2003. **14**: p. 334-40.
