# Supplementary material for: Effectiveness of community-based condom distribution interventions to prevent HIV in the United States: A systematic review and meta-analysis
Source: PLoS One. 2017 Aug 3;12(8):e0180718. doi: 10.1371/journal.pone.0180718 (PMC5542551; doi:10.1371/journal.pone.0180718)
Supplement: S6 File — (PDF) [file pone.0180718.s006.pdf]

S6: Pooled effect measures and risk of bias tables

Systematic Review of Community-based Condom Distribution Interventions in the US: UCSF CAPE Project

Contents

Fig 1. Pooled effect measures and risk of bias for the effect of "Ongoing" community-based condom distribution interventions (compared to no condom distribution) on sexual risk behaviors in the U.S. 1

Fig 2. Pooled effect measures and risk of bias for the effect of "Ongoing-plus" community-based condom distribution interventions (compared to no condom distribution) on sexual risk behaviors in the U.S. 3

Fig 3. Pooled effect measures and risk of bias for the effect of "Coupon-based" community-based condom distribution interventions (compared to no condom distribution) on sexual risk behaviors in the U.S. 5

Fig 1. Pooled effect measures and risk of bias for the effect of "Ongoing" community-based condom distribution interventions (compared to no condom distribution) on sexual risk behaviors in the U.S.

Outcome and study	Risk Ratio (95% CI)	Weight (%)	Heterogeneity (I^2)	Forest plot Random Effects Model <-Favors Intervention Favors comparator->	Risk of bias						
					A	B	C	D	E	F	G
**Condomless sex likelihood, all studies**				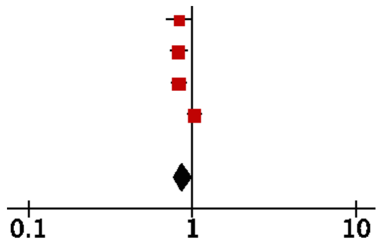							
Calsyn 1992	0.83 (0.69 to 1.0)	19			+	?	-	+	-	+	+
Cohen 1999	0.83 (0.73 to 0.93)[a,b]	25.71			?	?	-	+	-	+	+
Eisenberg 2013	0.83 (0.75 to 0.92)[c]	27.69			?	?	?	+	-	-	+
Ross 2004	1.03 (0.93 to 1.14)[d]	27.6			?	?	-	+	-	+	+
**TOTAL**	0.88 (0.78 to 0.99)	100	74.53								
**Condomless sex likelihood, males**				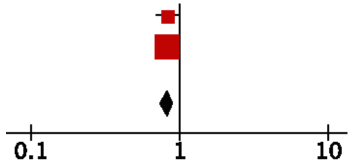							
Calsyn 1992	0.83 (0.69 to 1.0)	29.5			+	?	-	+	-	+	+
Cohen 1999	0.83 (0.73 to 0.93)[a,b]	70.5			?	?	-	+	-	+	+
**TOTAL**	0.83 (0.75 to 0.91)	100	0								
**Condomless sex likelihood, drug users**											
Calsyn 1992	0.83 (0.69 to 1.00)	100			+	?	-	+	-	+	+
**Condomless sex likelihood, follow-up ≤1 year**				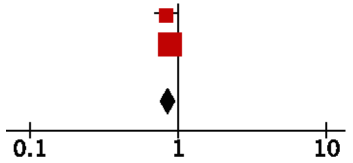							
Calsyn 1992	0.83 (0.69 to 1)	34.28			+	?	-	+	-	+	+
Cohen 1999	0.88 (0.77 to 1.01)[a]	65.72			?	?	-	+	-	+	+
**TOTAL**	0.86 (0.77 to 0.96)	100	0								
**Condomless sex likelihood, follow-up >1 year**				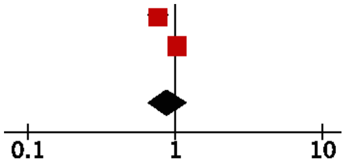							
Cohen 1999	0.76 (0.65 to 0.89)[a]	48.02			?	?	-	+	-	+	+
Ross 2004	1.03 (0.93 to 1.14)[d]	51.98			?	?	-	+	-	+	+
**TOTAL**	0.89 (0.66 to 1.2)	100	90.17								

**Multiple sexual partnership, all studies**				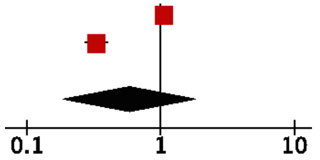							
Cohen 1999	1.06 (0.98 to 1.15)[a,b]	50.32			?	?	-	+	-	+	+
Ross 2004	0.33 (0.27 to 0.4)[d,e]	49.68			?	?	-	+	-	+	+
**TOTAL**	0.59 (0.19 to 1.86)	100	99.11								
**Multiple sexual partnership, males**											
Cohen 1999	1.06 (0.98 to 1.15)[a,b]	100			?	?	-	+	-	+	+
**Multiple sexual partnership, follow-up ≤1 year**											
Cohen 1999[1]	1.07 (0.98 to 1.17)[a]	100			?	?	-	+	-	+	+
**Multiple sexual partnership, follow-up >1 year**				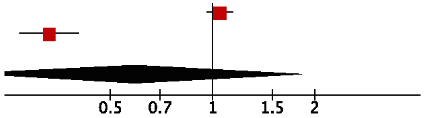							
Cohen 1999	1.05 (0.96 to 1.15)[a]	50.31			?	?	-	+	-	+	+
Ross 2004	0.33 (0.27 to 0.4)[d,e]	49.69			?	?	-	+	-	+	+
**TOTAL**	0.59 (0.19 to 1.84)	100	99.06								

Risk of bias legend:
(A) Incomplete outcome data (attrition bias)
(B) Selective reporting (reporting bias)
(C) Other bias
(D) Failure to develop and apply appropriate eligibility criteria
(E) Flawed measurement of exposure and/or outcome
(F) Failure to control for confounders
(G) Too-short or incomplete length of follow-up
- = high risk of bias
+ = low risk of bias
? = unclear risk of bias

Footnotes:
[a] Effect based on Area B only using a pre-post test design to focus on the effect of basic condom distribution only, and calculated using reported counts.
[b] Combined effect across 1995 (1 year) and 1996 (2 year) follow-up cross-sectional samples.
[c] Effect calculated using regression-coefficient from reported multilevel regression Model 1. P-value used to calculate CI was provided in personal communication with the author (Marla E. Eisenberg).
[d] Effect based on control group only, using a pre-post design.
[e] Effect reported as “Number of sex partners.” Converted to dichotomous variable (two or more partners) by assuming a Poisson distribution and no item non-response (CI calculated using 10,000 iterations of simulated samples).

Fig 2. Pooled effect measures and risk of bias for the effect of "Ongoing-plus" community-based condom distribution interventions (compared to no condom distribution) on sexual risk behaviors in the U.S.

Outcome and study	Risk Ratio (95% CI)	Weight (%)	Heterogeneity (I^2)	Forest plot<br>Random Effects Model<br><-Favors Intervention Favors comparator->	Risk of bias						
					A	B	C	D	E	F	G
**Condomless sex likelihood, all studies**				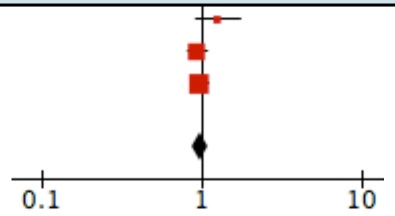							
Alstead 1999	1.25 (0.91 to 1.72)[c]	10.87			?	?	-	+	-	+	+
Lauby 2000	0.93 (0.81 to 1.07)[d]	40.33			?	?	-	+	-	+	+
Ross 2004	0.96 (0.86 to 1.08)	48.8			?	?	-	+	-	+	+
**TOTAL**	0.98 (0.88 to 1.09)[e]	100	34.67								
**Condomless sex likelihood, females**											
Lauby 2000	0.93 (0.81 to 1.07)[d]	100			?	?	-	+	-	+	+
**Condomless sex likelihood, follow-up ≤1 year**											
Alstead 1999	1.25 (0.91 to 1.72)[c]	100			?	?	-	+	-	+	+
**Condomless sex likelihood, follow-up >1 year**				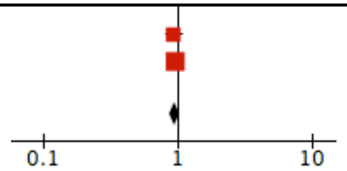							
Lauby 2000	0.93 (0.81 to 1.07)[d]	42.01			?	?	-	+	-	+	+
Ross 2004	0.96 (0.86 to 1.08)[f]	57.99			?	?	-	+	-	+	+
**TOTAL**	0.95 (0.87 to 1.04)[g]	100	0								
**Not always using condoms, all studies, follow-up >1 year, females**											
Lauby 2000	0.91 (0.71 to 1.17)[d]	100			?	?	-	+	-	+	+
**Multiple sexual partnership, all studies, follow-up >1 year**				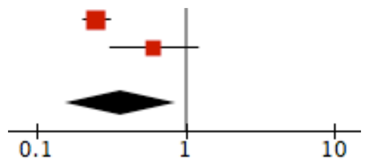							
Ross 2004	0.25 (0.20 to 0.31)[f,h]	56.89			?	?	-	+	-	+	+
Sellers 1994	0.61 (0.31 to 1.20)[b]	43.11			+	?	+	+	-	+	+
**TOTAL**	0.37 (0.16 to 0.87)[i]	100	83.40								
**Multiple sexual partnership, males**											
Sellers 1994	0.9 (0.43 to 1.88)[j]	100			+	?	+	+	-	+	+
**Multiple sexual partnership, females**											
Sellers 1994	0.06 (0.01 to 0.36)[a]	100			+	?	+	+	-	+	+

Risk of bias legend:
(A) Incomplete outcome data (attrition bias)
(B) Selective reporting (reporting bias)

(C) Other bias
(D) Failure to develop and apply appropriate eligibility criteria
(E) Flawed measurement of exposure and/or outcome
(F) Failure to control for confounders
(G) Too-short or incomplete length of follow-up
- = high risk of bias
+ = low risk of bias
? = unclear risk of bias

Footnotes:
[a] Outcome is rare, so reported odds ratio can be used as RR.
[b] Effect is combined across reported measures for males and females using fixed effect model. Random effect model generated the same values.
[c] Combined effect across interviewed youth who reported being exposed and those not exposed to the campaign. Assumed proportion of interviewed youth who were sexually active did not change over interview waves, and assumed no item non-response, given admission of sexual activity.
[d] Combined effect across main and other partners. For each effect, we assumed a counterfactual rate equal to baseline in intervention plus the difference between control follow-up and control baseline. Used CI for DID to generate a p-value, then used p-value to calculate a CI for the RR. RR is unadjusted, but, per publication, adjustment had little effect on DID estimates. Samples included in each estimate were almost entirely distinct (only 9% of sample had both a main and other partner), so we combined into a single effect using fixed effect model.
[e] This estimate was fairly stable (RR:0.98, 95% CI: 0.86, 1.11; I^2=34.67%, p=.20) when we recalculated it by directly entering main and other partner estimates from Lauby 2000 into the random effect model.
[f] Effect based on intervention group only, using a pre-post design.
[g] This estimate was stable (exact same RR and 95% CI, I^2=0%, p=.38) when we recalculated it by directly entering main and other partner estimates from Lauby 2000 into the random effect model.
[h] Effect reported as “Number of sex partners.” Converted to dichotomous variable by assuming a Poisson distribution and no item non-response (CI calculated using 10,000 iterations of simulated samples).
[i] This estimate was fairly stable (RR: 0.30, 95% CI: 0.10, 0.91; I^2 = 85.05%, p<.01) when we recalculated it by directly entering male and female estimates from Sellers 1994 into the random effect model.
[j] Reported odds ratio used as a reasonable approximation of RR because it is close to 1.

Fig 3. Pooled effect measures and risk of bias for the effect of "Coupon-based" community-based condom distribution interventions (compared to no condom distribution) on sexual risk behaviors in the U.S.

Outcome and study	Risk Ratio (95% CI) (Odds ratio where noted)	Weight (%)	Heterogeneity (I^2)	Risk of bias										
				A	B	C	D	E	F	G	H	I	J	K
**Incident STI, all studies, follow-up ≤1 year, males and females**														
Cohen 1992	0.91 (0.63 to 1.31)[a]	100	-	?	?	+	+	?	+	-	■	■	■	■
**Incident STI, follow-up ≤1 year, males**														
Cohen 1992	0.85 (0.56 to 1.29)	100	-	?	?	+	+	?	+	-	■	■	■	■
**Incident STI, follow-up ≤1 year, females**														
Cohen 1992	1.18 (0.52 to 2.68)	100	-	?	?	+	+	?	+	-	■	■	■	■
**Condomless sex likelihood, all studies, follow-up ≤1 year, females**														
Bull 2008	0.67 (0.47 to 0.96)[b]	100	-	■	■	■	■	?	+	+	+	-	+	+

Risk of bias legend:
(A) Allocation concealment (selection bias)
(B) Random sequence generation (selection bias)
(C) Blinding of participants and personnel (performance bias)
(D) Blinding of outcome assessment (detection bias)
(E) Incomplete outcome data (attrition bias)
(F) Selective reporting (reporting bias)
(G) Other bias
(H) Failure to develop and apply appropriate eligibility criteria
(I) Flawed measurement of exposure and/or outcome
(J) Failure to control for confounders
(K) Too-short or incomplete length of follow-up
- = high risk of bias
\+ = low risk of bias
? = unclear risk of bias
■ = Domain not rated. Risk of bias domains A-G are rated for randomized studies, and E-K are rated for observational studies.

Footnotes:
[a] Effect is combined across reported measures for males and females using fixed effect model. Random effect model generated the same values.
[b] Estimate is odds ratio from reported logistic regression model predicting condom use at last sex based on whether respondents reporting seeing one of the campaign posters.
