# Supplementary material for: Effectiveness of community-based condom distribution interventions to prevent HIV in the United States: A systematic review and meta-analysis
Source: PLoS One. 2017 Aug 3;12(8):e0180718. doi: 10.1371/journal.pone.0180718 (PMC5542551; doi:10.1371/journal.pone.0180718)
Supplement: S7 File — (PDF) [file pone.0180718.s007.pdf]

S7: GRADE tables

Systematic Review of Community-based Condom Distribution Interventions in the US: UCSF CAPE Project

Contents

TABLE 1: GRADE EVIDENCE PROFILES OF "ONGOING" COMMUNITY-BASED CONDOM DISTRIBUTION INTERVENTIONS (COMPARED TO NO CONDOM DISTRIBUTION) ON SEXUAL RISK BEHAVIORS IN THE U.S. 1

TABLE 2: GRADE EVIDENCE PROFILES OF "ONGOING-PLUS" COMMUNITY-BASED CONDOM DISTRIBUTION INTERVENTIONS (COMPARED TO NO CONDOM DISTRIBUTION) ON SEXUAL RISK BEHAVIORS IN THE U.S. 3

TABLE 3: GRADE EVIDENCE PROFILES OF "COUPON-BASED" COMMUNITY-BASED CONDOM DISTRIBUTION INTERVENTIONS (COMPARED TO NO CONDOM DISTRIBUTION) ON SEXUAL RISK BEHAVIORS IN THE U.S. 5

Table 1: GRADE evidence profiles of "Ongoing" community-based condom distribution interventions (compared to no condom distribution) on sexual risk behaviors in the U.S.

Question: Should Ongoing condom distribution without co-interventions (compared to no condom distribution) be used for preventing HIV infection in the US?
Date: December 10, 2015
Settings: United States
Bibliography: Calsyn 1992, Cohen 1999[a], Eisenberg 2013, Ross 2004[b,c]

Outcome: Change in HIV incidence (reported surrogate outcomes)	Design	Number of studies (number of participants)	Relative effect (95% CI)	Quality of the evidence (GRADE)	Comments
Condomless sex likelihood, all studies	Observational	4 (8,091)	**RR 0.88** (0.78 to 0.99)	⊕⊖⊖⊖ **VERY LOW**	Graded down for very serious risk of bias and serious indirectness.[d,e,f]
Condomless sex likelihood, males	Observational	2 (984)	**RR 0.83** (0.75 to 0.91)	⊕⊖⊖⊖ **VERY LOW**	Graded down for very serious risk of bias and serious indirectness.[d,e]
Condomless sex likelihood, drug users	Observational	1 (51)	**RR 0.83** (0.69 to 1.00)	⊕⊖⊖⊖ **VERY LOW**	Graded down for very serious risk of bias and serious indirectness.[d,e,g]
Condomless sex likelihood, follow-up ≤1 year [5 months-1 year]	Observational	2 (739)	**RR 0.86** (0.77 to 0.96)	⊕⊖⊖⊖ **VERY LOW**	Graded down for very serious risk of bias and serious indirectness.[d,e]
Condomless sex likelihood, follow-up >1 year [2 years]	Observational	2 (1,427)	**RR 0.89** (0.66 to 1.20)	⊕⊖⊖⊖ **VERY LOW**	Graded down for very serious risk of bias, serious inconsistency and serious indirectness.[d,e,h]

Multiple sexual partnership, all studies	Observational	2 (1,696)	**RR 0.59** (0.19 to 1.86)	⊕⊖⊖⊖ **VERY LOW**	Graded down for very serious risk of bias, serious inconsistency and serious indirectness.[d,e,h]
Multiple sexual partnership, males	Observational	1 (907)	**RR 1.06** (0.98 to 1.15)	⊕⊖⊖⊖ **VERY LOW**	Graded down for very serious risk of bias and serious indirectness.[d,e]
Multiple sexual partnership, follow-up ≤1 year [1 year]	Observational	1 (661)	**RR 1.07** (0.98 to 1.17)	⊕⊖⊖⊖ **VERY LOW**	Graded down for very serious risk of bias and serious indirectness.[d,e]
Multiple sexual partnership, follow-up >1 year [2 years]	Observational	2 (1,421)	**RR 0.59** (0.19 to 1.84)	⊕⊖⊖⊖ **VERY LOW**	Graded down for very serious risk of bias and serious indirectness.[d,e]

Abbreviations:
Risk ratio, RR; Confidence interval, CI

GRADE Working Group grades of evidence:

⊕⊕⊕⊕	**HIGH**	We are very confident that the true effect lies close to that of the estimate of the effect. Further research is unlikely to substantially change the estimate.
⊕⊕⊕⊖	**MODERATE**	We are moderately confident in the effect estimate: The true effect is likely to be close to the estimate of the effect, but there is a possibility that it is substantially different.
⊕⊕⊖⊖	**LOW**	Our confidence in the effect estimate is limited: The true effect may be substantially different from the estimate of the effect.
⊕⊖⊖⊖	**VERY LOW**	We have very little confidence in the effect estimate: The true effect is likely to be substantially different from the estimate of effect.

Footnotes:

[a] Analyzed using data from "Area B" only, treating it as a pre-post design.
[b] Analyzed using data from comparison neighborhood only, treating as a pre-post design.
[c] Our sample size includes all study participants because item-level response rate and/or actual number analyzed was not reported.
[d] Flawed measurement of exposure and outcome. All studies single-arm pre/post studies. Graded down by 2 for high risk of bias.
[e] Studies did not assess incident HIV. Graded down by 1 for indirectness.
[f] Participants in Calsyn study were all drug users. Relatively low weighting in pooled data; not graded down.
[g] Participants in Calsyn study were all drug users (only study providing data for this outcome). Graded down by 1 for indirectness.
[h] High heterogeneity, wide confidence interval. Graded down by 1 for inconsistency.

Table 2: GRADE evidence profiles of "Ongoing-plus" community-based condom distribution interventions (compared to no condom distribution) on sexual risk behaviors in the U.S.

Question: Should Ongoing-plus condom distribution with co-interventions (compared to no condom distribution) be used for preventing HIV infection in the US?
Date: December 10, 2015
Settings: United States
Bibliography: Alstead 1999[a], Lauby 2000[b], Ross 2004[c], Sellers 1994[d]

Outcome: Change in HIV incidence (reported surrogate outcomes)	Design	Number of studies (number of participants)	Relative effect (95% CI)	Quality of the evidence (GRADE)	Comments
Condomless sex likelihood, all studies	Observational	3 (>4,494)	**RR 0.98** (0.88 to 1.09)	⊕⊖⊖⊖ **VERY LOW**	Graded down for very serious risk of bias and serious indirectness.[e,f]
Condomless sex likelihood, females	Observational	1 (>3,229)	**RR 0.93** (0.81 to 1.07)	⊕⊖⊖⊖ **VERY LOW**	Graded down for very serious risk of bias and serious indirectness.[e,f]
Condomless sex likelihood, follow-up ≤1 year [1-7 months]	Observational	1 (424)	**RR 1.25** (0.91 to 1.72)	⊕⊖⊖⊖ **VERY LOW**	Graded down for very serious risk of bias and serious indirectness.[e,f]
Condomless sex likelihood, follow-up >1 year [2-3 years]	Observational	2 (3,653)	**RR 0.95** (0.87 to 1.04)	⊕⊖⊖⊖ **VERY LOW**	Graded down for very serious risk of bias and serious indirectness.[e,f]
Not always using condoms, all studies, follow-up >1 year [3 years], females	Observational	1 (>3,229)	**RR 0.91** (0.71 to 1.17)	⊕⊖⊖⊖ **VERY LOW**	Graded down for very serious risk of bias and serious indirectness. [e,f]
Multiple sexual partnership, all studies, follow-up >1 year [1.5-2 years]	Observational	2 (1,243)	**RR 0.37** (0.16 to 0.87)	⊕⊖⊖⊖ **VERY LOW**	Graded down for very serious risk of bias and serious indirectness. Not graded up for strong association. [e,f]

Multiple sexual partnership, males	Observational	1 (NR)	**RR 0.90** (0.43 to 1.88)	⊕⊖⊖⊖ **VERY LOW**	Graded down for very serious risk of bias and serious indirectness. [e,f]
Multiple sexual partnership, females	Observational	1 (NR)	**RR 0.06** (0.01 to 0.36)	⊕⊖⊖⊖ **VERY LOW**	Graded down for very serious risk of bias and serious indirectness. Not graded up for strong association. [e,f,g]

Abbreviations:
RR, risk ratio; CI, confidence interval; not reported, NR

GRADE Working Group grades of evidence:

⊕⊕⊕⊕	**HIGH**	We are very confident that the true effect lies close to that of the estimate of the effect. Further research is unlikely to substantially change the estimate.
⊕⊕⊕⊖	**MODERATE**	We are moderately confident in the effect estimate: The true effect is likely to be close to the estimate of the effect, but there is a possibility that it is substantially different.
⊕⊕⊖⊖	**LOW**	Our confidence in the effect estimate is limited: The true effect may be substantially different from the estimate of the effect.
⊕⊖⊖⊖	**VERY LOW**	We have very little confidence in the effect estimate: The true effect is likely to be substantially different from the estimate of effect.

Footnotes:

[a] Number analyzed not reported. We estimated the number in analysis by assuming that the proportion of youth who were sexually active did not change over the interview waves, and that there was no item non-response, given admission of sexual activity.
[b] Effect was combined across effects for ”main” and ”other” partners, 3,229 respondents had a main partners and 795 had an other partner. It was unclear how much the groups overlapped, so we’ve included the number with a main partner as a conservative sample size estimate for this study.
[c] Analyzed using data from the intervention neighborhood only, treating it as a pre-post design.
[d] Number analyzed reported for sample combined across males and females only, no subgroup sample size reported.
[e] Flawed measurement of exposure and outcome. All studies single-arm pre/post studies. Graded down by 2 for high risk of bias.
[f] Studies did not assess incident HIV. Graded down by 1 for indirectness.
[g] Not graded up for strong association due to very serious risk of bias and serious indirectness.

Table 3: GRADE evidence profiles of "Coupon-based" community-based condom distribution interventions (compared to no condom distribution) on sexual risk behaviors in the U.S.

Question: Should Coupon-based condom distribution with co-interventions (compared to no condom distribution) be used for preventing HIV infection in the US?
Date: December 10, 2015
Settings: United States
Bibliography: Cohen 1992, Bull 2008

Outcome: Change in HIV incidence (reported surrogate outcomes)	Design	Number of studies (number of participants)	Relative effect (95% CI)	Quality of the evidence (GRADE)	Comments
Incident STI, all studies, follow-up ≤1 year [6-9 months], males and females	RCT	1 (503)	**RR 0.91** (0.63 to 1.31)	⊕⊖⊖⊖ **VERY LOW**	Graded down for serious risk of bias, serious imprecision, and indirectness.[a,b,c]
Incident STI, follow-up ≤1 year [6-9 months], males	RCT	1 (301)	**RR 0.85** (0.56 to 1.29)	⊕⊖⊖⊖ **VERY LOW**	Graded down for serious risk of bias, serious imprecision, and indirectness.[a,b,c]
Incident STI, follow-up ≤1 year [6-9 months], females	RCT	1 (202)	**RR 1.18** (0.52 to 2.68)	⊕⊖⊖⊖ **VERY LOW**	Graded down for serious risk of bias, serious imprecision, and indirectness.[a,b,c]
Condomless sex likelihood, all studies, follow-up ≤1 year [7-10 months], females	Observational	1 (2,005)	**OR 0.67** (0.47 to 0.96)	⊕⊖⊖⊖ **VERY LOW**	Graded down for very serious risk of bias and serious indirectness.[c,d]

Abbreviations:
RCT, randomized controlled trial; STI, sexually transmitted infection; RR, risk ratio; CI, confidence interval

GRADE Working Group grades of evidence:

⊕⊕⊕⊕	**HIGH**	We are very confident that the true effect lies close to that of the estimate of the effect. Further research is unlikely to substantially change the estimate.
⊕⊕⊕⊖	**MODERATE**	We are moderately confident in the effect estimate: The true effect is likely to be close to the estimate of the effect, but there is a possibility that it is substantially different.
⊕⊕⊖⊖	**LOW**	Our confidence in the effect estimate is limited: The true effect may be substantially different from the estimate of the effect.
⊕⊖⊖⊖	**VERY LOW**	We have very little confidence in the effect estimate: The true effect is likely to be substantially different from the estimate of effect.

Footnotes:

[a] Differential sensitivity of outcome. Participants randomized to control session were excluded if they had also been to one of the intervention sessions, meaning that control group systematically excluded people with more visits over the study period. Graded down by 1 for high risk of bias.

[b] Few events <200. Graded down by 1 for serious imprecision.

[c] Studies did not assess incident HIV. Graded down by 1 for indirectness.

[d] Flawed measurement of exposure and outcome. All studies single-arm pre/post studies. Graded down by 2 for high risk of bias.
